# Benchmarking clustering, alignment, and integration methods for spatial transcriptomics

**DOI:** 10.1186/s13059-024-03361-0

**Published:** 2024-08-09

**Authors:** Yunfei Hu, Manfei Xie, Yikang Li, Mingxing Rao, Wenjun Shen, Can Luo, Haoran Qin, Jihoon Baek, Xin Maizie Zhou

**Affiliations:** 1 Department of Computer Science, Vanderbilt University, 37235 Nashville, USA; 2 Department of Biomedical Engineering, Vanderbilt University, 37235 Nashville, USA; 3 Department of Bioinformatics, Shantou University Medical College, 515041 Shantou, China

**Keywords:** Spatial transcriptomics, Benchmarking, Clustering, Alignment, Integration, Batch correction, 3D reconstruction

## Abstract

**Background:**

Spatial transcriptomics (ST) is advancing our understanding of complex tissues and organisms. However, building a robust clustering algorithm to define spatially coherent regions in a single tissue slice and aligning or integrating multiple tissue slices originating from diverse sources for essential downstream analyses remains challenging. Numerous clustering, alignment, and integration methods have been specifically designed for ST data by leveraging its spatial information. The absence of comprehensive benchmark studies complicates the selection of methods and future method development.

**Results:**

In this study, we systematically benchmark a variety of state-of-the-art algorithms with a wide range of real and simulated datasets of varying sizes, technologies, species, and complexity. We analyze the strengths and weaknesses of each method using diverse quantitative and qualitative metrics and analyses, including eight metrics for spatial clustering accuracy and contiguity, uniform manifold approximation and projection visualization, layer-wise and spot-to-spot alignment accuracy, and 3D reconstruction, which are designed to assess method performance as well as data quality. The code used for evaluation is available on our GitHub. Additionally, we provide online notebook tutorials and documentation to facilitate the reproduction of all benchmarking results and to support the study of new methods and new datasets.

**Conclusions:**

Our analyses lead to comprehensive recommendations that cover multiple aspects, helping users to select optimal tools for their specific needs and guide future method development.

**Supplementary Information:**

The online version contains supplementary material available at 10.1186/s13059-024-03361-0.

## Background

Spatial transcriptomics (ST) technology, emerging as a complementary approach to scRNA-seq, facilitates comprehensive gene expression profiling in tissue samples while preserving the spatial information of every cell or spot analyzed [1, 2]. ST techniques have significantly enhanced our understanding of cellular heterogeneity and tissue organization, offering insights into developmental processes, disease mechanisms, and potential therapeutic strategies [3–6]. ST technologies are commonly categorized into two groups: imaging-based and sequencing-based methods [7–13]. Advancements in spatial resolution, capture capabilities, and computational methods are continuously enhancing their potential applications and capabilities.

An essential initial step in ST research is to cluster the spots and define spatially coherent regions in terms of expression data and location adjacency [14, 15]. This process essentially entails classical unsupervised clustering of spots into groups according to the similarity of their gene expression profiles and spatial locations, subsequently assigning labels to each cluster. To date, existing clustering methods in ST can be broadly categorized into two groups: statistical methods and graph-based deep learning methods [16].

Representative methods for statistical models are BayesSpace [17], BASS [18], SpatialPCA [19], DR.SC [20], and BANSKY [21]. BayesSpace performs spatial clustering at the spot level, utilizing a t-distributed error model to identify clusters, along with employing Markov chain Monte Carlo (MCMC) for estimating model parameters. BASS detects spatial domains and clusters cell types within a tissue section simultaneously by utilizing a hierarchical Bayesian model framework. BASS can also be applied to perform multi-slice clustering. SpatialPCA is a dimension reduction method aimed at extracting a low-dimensional representation of ST data using spatial correlation information. DR.SC employs a two-layer hierarchical model that simultaneously performs dimension reduction and spatial clustering, optimizing the extraction of low-dimensional features as well as the identification of spatial clusters. The BANSKY algorithm clusters cells using an azimuthal Gabor filter (AGF)-inspired kernel to capture gene expression variations. It constructs a neighborhood graph, computes z-scaled average neighborhood expression and AGF matrices, and combines these with the original gene expression data. This is followed by dimension reduction and graph-based clustering to determine cell types and domains.

Recent trends indicate a growing momentum toward utilizing graph-based deep learning backbones, attributed to their ability for graphing cell relations and capturing representative features. Representative methods are SpaGCN [22], SEDR [23], CCST [24], STAGATE [3], conST [25], ConGI [26], SpaceFlow [27], GraphST [4], and ADEPT [28]. These methods predominantly employ graph neural network models to extract latent spot features prior to clustering, albeit with variations in network architectures and design strategies. SpaGCN has a unique design of building an adjacency matrix while considering histology image pixel values. SEDR employs multiple variation autoencoders to handle data from different modalities. CCST is based on a graph convolutional network to improve cell clustering and discover novel cell types. STAGATE learns low-dimensional latent embeddings with both spatial information and gene expressions via a graph attention auto-encoder. conST, ConGI, and GraphST all rely on a contrastive learning strategy [29]. conST adopts a two-phase training strategy incorporating self-supervised contrastive learning at three levels: local-local, local-global, and local-context. ConGI utilizes three different contrastive learning losses to integrate information from both the histology images as well as the gene expression profiles. GraphST utilizes representations of both normal graphs and corrupted graphs to construct positive and negative spot pairs for contrastive training. SpaceFlow uses spatially regularized deep graph networks to create spatially-consistent low-dimensional embeddings. This framework introduces a pseudo-spatiotemporal map to integrate pseudotime with spatial locations. ADEPT employs differentially expressed gene selection and imputation procedures to minimize the variations in prediction.

In contrast to merely identifying spatial domains or cell types within a single slice, there is an increasing acknowledgment of the importance of integrative and comparative analyses of multiple ST slices [30]. Thus, ST analysis tools might integrate samples originating from diverse sources, encompassing various individual samples, biological conditions, technological platforms, and developmental stages. Nonetheless, ST slices may exhibit significant “batch effects” [15], which refer to technical biases such as uneven amplification during PCR [31], variations in cell lysis [32], or differences in reverse transcriptase enzyme efficiency during sequencing. These factors have the potential to obscure genuine biological signals, thereby complicating data interpretation and integration.

To analyze multiple ST slices by minimizing batch effects, different alignment and integration methods have been introduced. Alignment methods are designed to align or match spots or cells from different ST sections or datasets to a common spatial or anatomical reference. These methods are critical for correcting distortions or differences in tissue sections, ensuring consistency across samples. Integration methods primarily merge data from various sources or conditions to create a comprehensive dataset, enhancing data robustness and revealing broader patterns not apparent in individual datasets. These techniques excel at adjusting for batch effects and normalizing data. Some tools can perform both alignment and integration tasks. Representative alignment methods include PASTE [33], PASTE2 [34], SPACEL [35], STalign [36], and GPSA [37]. PASTE utilizes the Gromov-Wasserstein optimal transport (OT) algorithm [38] for aligning adjacent consecutive ST data. PASTE2, an extension of PASTE, allows partial alignment, accommodating partial overlap between aligned slices and/or slice-specific cell types. Both PASTE and PASTE2 output a mapping matrix for every pair of consecutive ST slices, facilitating the reconstruction of the tissue’s 3D architecture through multi-slice alignment. SPACEL combines a multi-layer perceptron and a probabilistic model for deconvolution. It subsequently employs a graph convolutional network with adversarial learning to identify spatial domains across multiple ST slices and finally constructs the 3D tissue architecture by transforming and stacking the spatial coordinate systems of consecutive slices. STalign aligns ST datasets across sections, samples, and technologies by using diffeomorphic metric mapping to account for partially matched tissue sections and local non-linear distortions. GPSA is a probabilistic model that employs a two-layer Gaussian process where the first layer maps observed spatial locations to a common coordinate system (CCS), and the second layer maps from the CCS to the observed phenotypic readouts, such as gene expression.

Several integration methods have also been introduced. Notable examples include STAligner [39], DeepST [40], PRECAST [41], and SPIRAL [42]. These tools do not directly align slices; instead, they learn shared latent spot embeddings after jointly training on multiple slices. STAligner, built on the STAGATE model, introduces triplet loss by utilizing mutual nearest neighbors between spots from consecutive slices to exploit the contrastive learning strategy for enhancing inter-slice connection. DeepST consists of a graph neural network autoencoder and a denoising autoencoder to generate a representation of the augmented ST data as well as domain adversarial neural networks to integrate ST data. DeepST is also applicable to individual slices for spatial clustering. PRECAST leverages a unified model including a hidden Markov random field model and a Gaussian mixture model to simultaneously tackle low-dimensional embedding estimation, spatial clustering, and alignment embedding across multiple ST datasets. SPIRAL employs a graph autoencoder backbone with an OT-based discriminator and a classifier to remove the batch effect, align coordinates, and enhance gene expression. BASS applies a hierarchical Bayesian model framework for multi-slice clustering and outputs clustering labels.

The dichotomization of alignment and integration methods is not absolute. PASTE also outputs an integrated center slice, so it can also be classified as an integration tool. STAligner and SPIRAL are also capable of aligning multiple adjacent slices to construct a 3D architecture. For simplicity, we classified each tool into either the alignment or integration category.

**Fig. 1:**
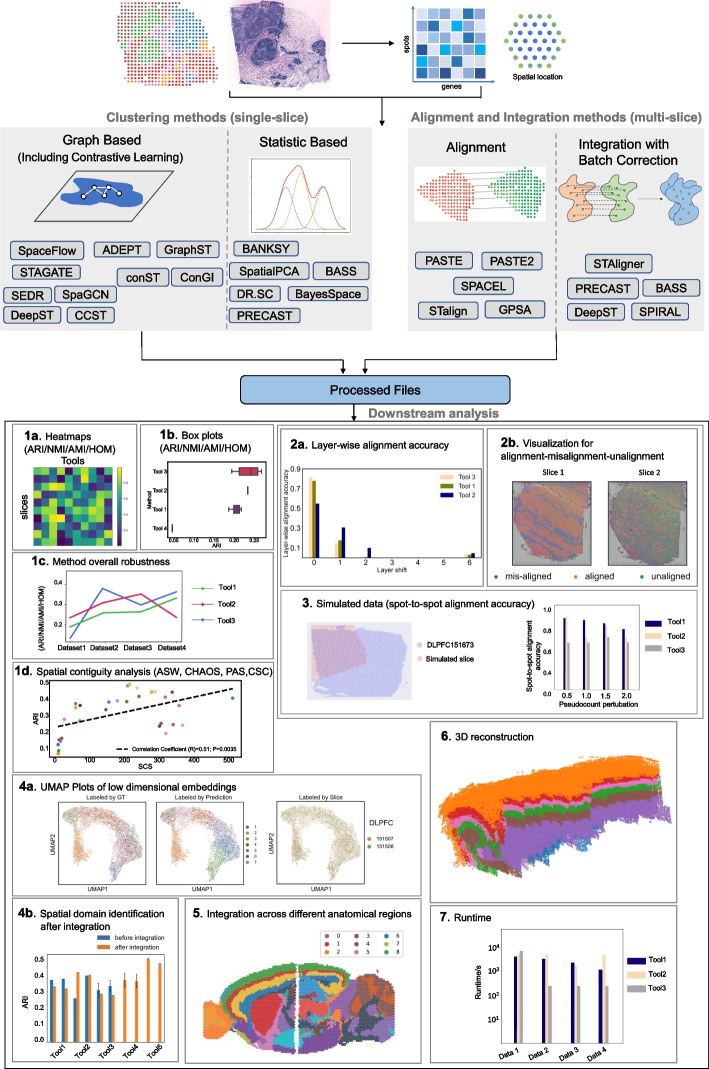
Benchmarking framework for clustering, alignment, and integration methods on different real and simulated datasets. Top, illustration of the set of methods benchmarked, which includes 16 clustering methods, five alignment methods, and five integration methods. Bottom, overview of the benchmarking analysis, in terms of different metrics (1–7). Different experimental metrics and analyses, Adjusted Rand Index (ARI), Normalized Mutual Information (NMI), Adjusted Mutual Information (AMI), Homogeneity (HOM), Average Silhouette Width (ASW), CHAOS, Percentage of Abnormal Spots (PAS), Spatial Coherence Score (SCS), uniform manifold approximation and projection (UMAP) visualization, layer-wise and spot-to-spot alignment accuracy, 3D reconstruction, and runtime, are designed to quantitatively and qualitatively assess method performance as well as data quality. Additional details are provided in the “Results” section

Although clustering, alignment, and integration methods have enhanced our understanding of ST data and their practical applications, the lack of comprehensive benchmarking constrains comparison and hampers further algorithm development. It is common for a method to demonstrate excellent performance on well-studied, commonly used datasets; however, its performance may vary significantly when applied to brand-new data. In this work, we systematically analyze and evaluate the performance of 16 state-of-the-art clustering methods, five alignment methods, and five integration methods on a multitude of simulated and real ST datasets. We design a comprehensive benchmark framework in Fig. 1 and evaluate the clustering performance, overall robustness, layer-wise and spot-to-spot alignment accuracy, integration performance, 3D reconstruction, and computing time of each method. We consolidate these findings into a comprehensive recommendation spanning multiple aspects for the users, while also spotlighting potential areas in need of further research.

## Results

### ST datasets examined and data preprocessing

We collected 10 ST datasets with a total of 68 slices for benchmarking, which had corresponding manual annotations shown in Table 1. These datasets were produced by several ST protocols, including 10x Visium, ST, Slide-seq v2, Stereo-seq, STARmap, and MERFISH. We broadly categorized them into two groups based on the methodology employed-sequencing-based or imaging-based. The datasets varied in size, with the number of spots ranging from approximately 200 to over 50,000 and the number of genes from 150 to approximately 36,000.

**Table 1:** Benchmark tools and datasets

**Algorithm**	**Task**	**Language**	**Resource**	**Output**	**Method**	**Link**
BANKSY	Clustering	R	Singhal et al. 2024 [21]	Clustering Labels, Embedding	Azimuthal Gabor filter	https://github.com/prabhakarlab/Banksy
ADEPT	Clustering	Python	Hu et al. 2023 [28]	Clustering Labels, Embedding	Graph Autoencoder, Imputation, Differentially Expressed Genes	https://github.com/maiziezhoulab/ADEPT
GraphST	Clustering	Python	Long et al. 2022 [4]	Clustering Labels, Embedding	Graph Neural Network, Contrastive Learning	https://github.com/JinmiaoChenLab/GraphST
SpaceFlow	Clustering	Python	Ren et al. 2022 [27]	Clustering Labels, Embedding	Spatially Regularized Deep Graph Infomax	https://github.com/hongleir/SpaceFlow
conST	Clustering	Python	Zong et al. 2022 [25]	Clustering Labels, Embedding	Contrastive Learning, Masked Autoencoder	https://github.com/ys-zong/conST
ConGI	Clustering	Python	Zeng et al. 2022 [26]	Clustering Labels, Embedding	Contrastive Learning, Autoencoder	https://github.com/biomed-AI/ConGI
SpatialPCA	Clustering	R	Shang et al. 2022 [19]	Clustering Labels	Spatial Probabilistic PCA	https://github.com/shangll123/SpatialPCA
DR.SC	Clustering	R	Liu et al. 2022 [20]	Clustering Labels	Regression Analysis, Imputation	https://github.com/feiyoung/DR-SC.Analysis
STAGATE	Clustering	Python	Dong et al. 2022 [3]	Clustering Labels, Embedding	Graph Autoencoder, Attention Mechanism	https://github.com/QIFEIDKN/STAGATE_pyG
CCST	Clustering	Python	Li et al. 2022 [24]	Clustering Labels, Embedding	Graph Convolutional Network	https://github.com/xiaoyeye/CCST
SEDR	Clustering	Python	Fu et al. 2021 [23]	Clustering Labels, Embedding	Deep Autoencoder, Graph Variational Autoencoder	https://github.com/JinmiaoChenLab/SEDR
SpaGCN	Clustering	Python	Hu et al. 2021 [22]	Clustering Labels	Graph Convolutional Network	https://github.com/jianhuupenn/SpaGCN
BayesSpace	Clustering	R	Li et al. 2021 [17]	Clustering Labels	Markov chain Monte Carlo, Differentially Expressed Genes	https://github.com/edward130603/BayesSpace
STalign	Alignment	Python	Clifton et al. 2023 [36]	Refined Coordinates	Diffeomorphic Metric Mapping	https://github.com/JEFworks-Lab/STalign
GPSA	Alignment	Python	Jones et al. 2023 [37]	Refined Coordinates	Gaussian Process	https://github.com/andrewcharlesjones/spatial-alignment
SPIRAL	Integration	Python	Guo et al. 2023 [42]	Refined Coordinates, Embedding	GraphSAGE, Optimal Transport	https://github.com/guott15/SPIRAL
STAligner	Integration	Python	Zhou et al. 2023 [39]	Embedding	Graph Autoencoder, Attention Mechanism, Triplet Loss	https://github.com/zhanglabtools/STAligner
PASTE	Alignment Integration	Python	Zeira et al. 2022 [33]	Alignment Matrix, Integrated Slice, 3D Reconstruction	Fused Gromov-Wasserstein Optimal Transport, Generalized Procrustes Analysis	https://github.com/raphael-group/paste
PASTE2	Alignment Integration	Python	Xinhao et al. 2022 [34]	Partial Alignment Matrix, 3D Reconstruction	Partial Fused Gromov-Wasserstein Optimal Transport, Generalized Procrustes Analysis	https://github.com/raphael-group/paste2
PRECAST	Clustering Integration	R	Liu et al. 2023 [41]	Embedding	Gaussian Mixture Model, Discrete Hidden Markov Random Field	https://github.com/cran/PRECAST
SPACEL	Deconvolution Clustering Alignment	Python	Xu et al. 2023 [35]	Cell Type Composition, Refined Coordinates, 3D Reconstruction	Variational Autoencoder, Graph Convolutional Network, Adversarial Learning, Regression	https://github.com/QuKunLab/SPACEL
BASS	Clustering Integration	R	Li et al. 2022 [18]	Clustering Labels	Bayesian Analysis, Multi-sample Analysis	https://github.com/zhengli09/BASS
DeepST	Clustering Integration	Python	Xu et al. 2022 [40]	Embedding	Data Augmentation, Variational graph autoencoder	https://github.com/JiangBioLab/DeepST
**ST dataset**	**ST type**	**Abbreviations**	**ST protocol**	**Spots/genes**	**Num. of used slices**	**Source**
Dataset 1: Human Dorsal Lateral Prefrontal Cortex data [43]	Sequencing-based	DLPFC	10x Visium	3431-4788/33,538	12	http://spatial.libd.org/spatialLIBD/
Dataset 2: Human Breast Cancer Block A Section 1 [44]	Sequencing-based	HBCA1	10x Visium	3798/36,601	1	https://support.10xgenomics.com/spatial-gene-expression/datasets/1.1.0/V1_Breast_Cancer_Block_A_Section_1
Dataset 3: Mouse Brain Section 2 Sagittal Anterior [45]	Sequencing-based	MB2SA	10x Visium	2695/32,285	2	https://www.10xgenomics.com/resources/datasets/mouse-brain-serial-section-2-sagittal-anterior-1-standard
Dataset 4: HER2 Positive Breast Tumors [46]	Sequencing-based	HER2BT	Spatial Transcriptomics	177-692/ 14,861-15,701	8	https://github.com/almaan/her2st
Dataset 5: Mouse Hippocampus [47]	Sequencing-based	MHPC	Slide-seq v2	41,770/23,264	1	https://singlecell.broadinstitute.org/single_cell/study/SCP815
Dataset 6: MOSTA Embryo [48]	Sequencing-based	Embryo	Stereo-seq	30,124-51,365/26,854-27,810	2	https://db.cngb.org/stomics/mosta/resource/
Dataset 7: Mouse Visual Cortex [9]	Sequencing-based	MVC	STARmap	1207/1020	1	https://www.STARmapresources.com/data
Dataset 8: Mouse Prefrontal Cortex [9]	Sequencing-based	MPFC	STARmap	1049-1088/166	3	https://github.com/zhengli09/BASS-Analysis/blob/master/data/STARmap_mpfc.RData
Dataset 9: Mouse Hypothalamus [49]	Imaging-based	MHypo	MERFISH	5488-5926/155	5	https://datadryad.org/stash/dataset/doi:10.5061/dryad.8t8s248
Dataset 10: Mouse Brain [50]	Sequencing-based	MB	MERFISH	2033-7626/254	33	https://zenodo.org/records/8167488

Top panel: Summary of clustering, alignment, and integration methods used in this work. The tool’s task, programming language, resource, tool output, the general method by each tool, and tool links are shown in the table. Bottom panel: Overview of the datasets benchmarked in this study. The ST type, datasets’ abbreviations, ST protocol, the range of number of spots and genes, number of slices, and source link for each dataset are shown in the table

Specifically, (1) the DLPFC dataset, generated with 10x Visium, includes 12 human DLPFC sections with manual annotation, indicating cortical layers 1 to 6 and white matter (WM), taken from three individual samples [51]. Each sample contains four consecutive slices (for example, slice A, B, C, and D in order). In each sample, the initial pair of slices, AB, and the final pair, CD, are directly adjacent (10 µm apart), whereas the intermediate pair, BC, is situated 300 µm apart.

(2) The HBCA1 dataset, generated with 10x Visium, includes a single slice of human breast cancer, which is open-sourced from 10x genomics [23].

(3) The MB2SA&P dataset, generated with 10x Visium, includes two slices of the anterior and posterior mouse brain. Only the anterior section includes annotation [12, 26].

(4) The HER2BT dataset [46] by spatial transcriptomics contains HER2-positive tumors from eight individuals (patients A–H). Each slice contains between 177 and 692 spots and was examined and annotated by a pathologist based on morphology. Regions were labeled as either: cancer in situ, invasive cancer, adipose tissue, immune infiltrate, breast glands, or connective tissue.

(5) The MHPC dataset [19] by Slide-seq v2 is the largest slice used in our study with over 40,000 spots and 23,000 genes. The Allen Mouse Brain Atlas [52] was used as ground truth to identify seven key anatomical regions of the hippocampus, namely CA1, CA2, CA3, dentate gyrus (DG), third ventricle (V3), medial habenula (MH), and lateral habenula (LH). The cell-type annotations were provided by Goeva and Macosko [53].

(6) The Embryo dataset by Stereo-seq has over 50 slices, and the slices at two different time points E11.5 and E12.5 were used in our experiments. These datasets are from a large stereo-seq project called MOSTA [48]: Mouse Organogenesis Spatiotemporal Transcriptomic Atlas by BGI.

(7) The MVC dataset [9] by STARmap contains one slice and was generated from the mouse visual cortex. It extends from the hippocampus (HPC) to the corpus callosum (CC) and includes the six neocortical layers.

(8) The MPFC dataset [9] of the mouse prefrontal cortex, annotated by BASS [18], was sequenced with the STARmap protocol. This dataset includes expression values for 166 genes measured across 1049 to 1088 single cells, along with their centroid coordinates on the tissue. Spatial domains, such as cortical layers L1, L2/3, L5, and L6, have been assigned based on the spatial expression patterns of marker genes, including Bgn for L1, Cux2 for L2/3, Tcerg1l for L5, and Pcp4 for L6. Three slices in this dataset are not categorized as consecutive.

(9) The MHypo dataset by MERFISH contains five manually annotated consecutive slices [18] labeled Bregma -0.04 mm (5488 cells), Bregma -0.09 mm (5557 cells), Bregma -0.14 mm (5926 cells), Bregma -0.19 mm (5803 cells), and Bregma -0.24 mm (5543 cells). Expression measurements were taken for a common set of 155 genes. Each tissue slice includes a detailed cell annotation, identifying eight structures: third ventricle (V3), bed nuclei of the stria terminalis (BST), columns of the fornix (fx), medial preoptic area (MPA), medial preoptic nucleus (MPN), periventricular hypothalamic nucleus (PV), paraventricular hypothalamic nucleus (PVH), and paraventricular nucleus of the thalamus (PVT).

Finally, (10) the MB dataset [35, 50] by MERFISH has 33 consecutive mouse primary motor cortex tissue slices with similar shapes, which can be used for 3D reconstruction. Region annotation includes the six layers (L1-L6) and white matter (WM). Further details about the ground truth for each dataset are outlined in Additional file 1: Table S1. All except the MB dataset were used for benchmarking clustering tools. Five datasets, DLPFC, MB2SA&P, Embryo, MHypo, and MB, were used for benchmarking alignment and integration tools. Utilizing the evaluation framework illustrated in Fig. 1, we conducted benchmarking of various clustering, alignment, and integration methods across all ST datasets.

All methods employ customized and often inconsistent preprocessing strategies, which might significantly impact their performance. The preprocessing of ST data typically encompasses four essential steps: quality control, normalization, feature selection, and/or dimension reduction. Each method may employ one or more of these steps. The scanpy package is commonly used to eliminate low-quality cells that lack sufficient expressed transcripts or low-quality genes that are rarely observed across the data slice, thereby mitigating the impact of noise. Subsequently, the expression matrix is normalized within each cell and log-transformed to further suppress potential extreme values. Feature selection involves any form of expression profile dimension reduction or subsetting steps. Due to the variability in preprocessing steps across different methods, it is challenging to draw a simple conclusion. Therefore, we have summarized the parameter settings and descriptions used in the preprocessing steps when benchmarking each method in Additional file 1: Table S2. For instance, STAGATE selects only highly variable genes (HVGs), while CCST and conST calculate principal components (PCAs) to reduce the input feature dimensions. SpaceFlow and ADEPT utilize HVGs but also emphasize input feature quality control by removing noisy genes and samples. Regarding alignment and integration methods, for example, STAligner, SPIRAL, and GPSA incorporate preprocessing in their workflows. All three select HVGs, but only GPSA also controls data quality by removing low-quality genes and cells. We also provided the specific pipeline of data preprocessing for each method in our GitHub.

PCA is commonly used for dimensionality reduction in clustering methods. GLM-PCA [54] is believed to improve low-dimensional representation compared to PCA. As detailed in Additional file 2: Supplementary results and Fig. S1, we analyzed whether replacing principal components (PCs) with GLM-PCs enhances performance.

### Performance comparison of 16 clustering methods

**Fig. 2:**
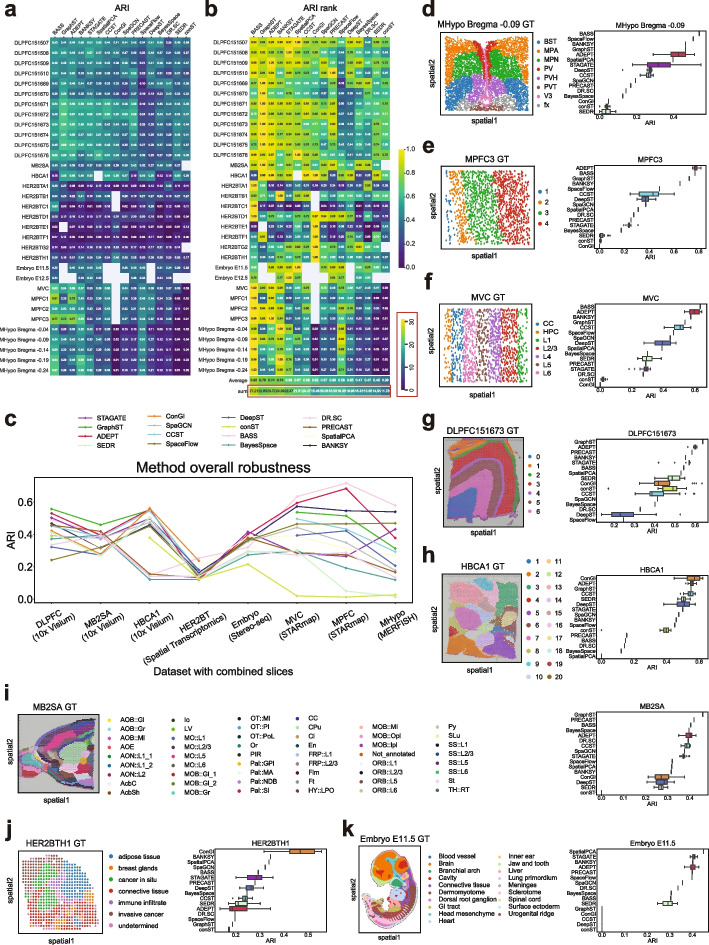
Clustering performance over 16 methods on 33 ST slices of eight datasets. **a** ARI heatmap. Each average ARI value is based on 20 runs. Empty entries for specific tools indicate either that the tool is not optimized for those use cases or that technical issues prevent the tool from completing its execution. **b** Ranking heatmap. This ranking heatmap is created by normalizing all results within the same slice by dividing them by the maximum ARI value (representing the best performance) among all methods, thus standardizing all ARI values to 1. For each method, the best ranking for the sum result is 33, and the best ranking for the average result is 1. The two heatmaps in (**a**, **b**) share a color bar ranging from 0 to 1. **c** Line plots illustrating the overall robustness of all methods across eight datasets in terms of ARI. **d**–**k** Ground truth visualization plots and box plots depicting ARI values from 20 runs of all tools on selected data slices from each dataset. The box plots illustrate the variability in the ARI on individual slices for certain tools since they do not use a fixed seed. In the box plots, the center line, box limits, and whiskers denote the median, upper and lower quartiles, and 1.5$\times$ interquartile range, respectively. Certain tools were not applicable to specific datasets, so for the purpose of ordering, their ARI values in the box plots were assigned a value of 0

We first performed a comprehensive benchmarking analysis for 16 different clustering methods aimed at assessing their performance in accurately identifying spatial domains. The two heatmaps of Fig. 2a, b illustrated the average Adjusted Rand Index (ARI) for each method across 33 slices from eight ST datasets, along with the corresponding rank scores for each tool. We ranked the tools in descending order based on their average rank of ARI. Details for computing ARI values and rank score are included in the “Methods” section. The ARI and rank results revealed that BASS, GraphST, ADEPT, BANKSY, and STAGATE emerged as top-tier tools, followed by SpatialPCA and CCST. Notably, BASS attained the highest average and sum rank, followed by GraphST, ADEPT, and BANKSY. BASS achieved a much higher ARI than other methods on the MHypo datasets. Most methods struggled to give reasonable predictions on the HER2BT datasets since the annotated regions by ground truth were less coherent and the data more noisy. This comprehensive evaluation shed light on the relative strengths of these methods in the context of spatial domain identification within each ST slice.

In Fig. 2c, we further present a holistic assessment of the overall robustness of each clustering method by aggregating the average ARI across slices within each of the eight datasets and depict the results in a line chart. Notably, lower variances were exhibited in the DLPFC, MB2SA (the anterior section of MB2SA&P), HER2BT, and Embryo datasets across all clustering methods, albeit for different reasons. BASS, in alignment with previous analyses, emerged as the best clustering tool for four datasets. Nevertheless, it exhibited comparatively poorer performance on the HBCA1 dataset. ADEPT and BANKSY consistently secured the second and third positions, respectively, across most datasets, while GraphST led in the DLPFC and MB2SA datasets. The two slices from the Embryo dataset, each containing approximately 30,000 and 50,000 cells, respectively, were used to investigate the scalability of various methods. GraphST, CCST, and DeepST were not applicable to either slice due to memory constraints. ADEPT, SpaGCN, SEDR, and conST were not applicable to one of the slices (Embryo E12.5) for the same reason. Among all the tools, STAGATE achieved the highest overall performance in terms of ARI across both Embryo slices.

Although we have highlighted top tools based on overall performance across all slices and datasets, certain tools may perform exceptionally well or experience performance degradation in datasets for specific ST protocols or tissue types. For instance, GraphST performed best in 10x Visium datasets but experienced a decline in performance with the STARmap and MERFISH datasets, which were not specialized data types for GraphST (Fig. 2c, dark green line). STAGATE (Fig. 2c, purple line) performed the best for the Stereo-seq Embryo dataset, but its accuracy ranking was not at the top for other protocol datasets. SpaceFlow ranked third for the MERFISH (imaging-based) dataset but did not perform well for other sequencing-based datasets (Fig. 2c, olive line). ConGI achieved top accuracy in both tumor slice datasets (HBCA1 and HER2BT), but did not perform well in brain slice datasets (Fig. 2c, orange line).

### Random seed analysis

Since the mean ARI does not capture the variance of each method, we also plotted box plots and ground truth visualization plots on all slices from each dataset (Fig. 2d–k and Additional file 2: Fig. S2-S3). All six statistical methods, namely BASS, BayesSpace, DR.SC, PRECAST, SpatialPCA, and BANKSY, exhibited no variance as they set fixed seed for the initialization of parameters inside their functions. The remaining methods primarily relied on graph-based deep learning techniques, leading to potential variations in their predictions owing to random seeds. However, GraphST, ConGI, SpaGCN, and SpaceFlow also fixed their seeds to be identical for each run. In contrast, some deep learning-based methods do not adhere to this practice. To investigate the impact of random seeds and the corresponding loss function or objective function values on the clustering accuracy of these methods, we selected deep learning-based methods (CCST, ADEPT, and STAGATE) and statistical methods (BayesSpace and BASS) for additional analysis. The plots of ARI versus loss value, ARI versus seed, and loss value versus seed for the three deep learning-based methods indicated that clustering performance, measured by ARI, was randomly associated with both the loss value and the selected seed for each deep learning method (Additional file 2: Fig. S4-S6), making it challenging to select a particular result. However, these findings suggested that all three tools exhibited variance in ARI across various individual DLPFC slices, consistent with previous box plots for all slices (Fig. 2d–k and Additional file 2: Fig. S2). A similar analysis on random seed, objective function value, and ARI for the statistical methods BayesSpace and BASS yielded the same result: clustering performance, in terms of ARI, was randomly associated with both the objective function value and the selected seed (Additional file 2: Fig. S7-S8). For BASS, we did not use the objective function value since it does not have one, but only the random seed and performance.

### Clustering performance comparison using NMI, AMI and HOM

**Fig. 3:**
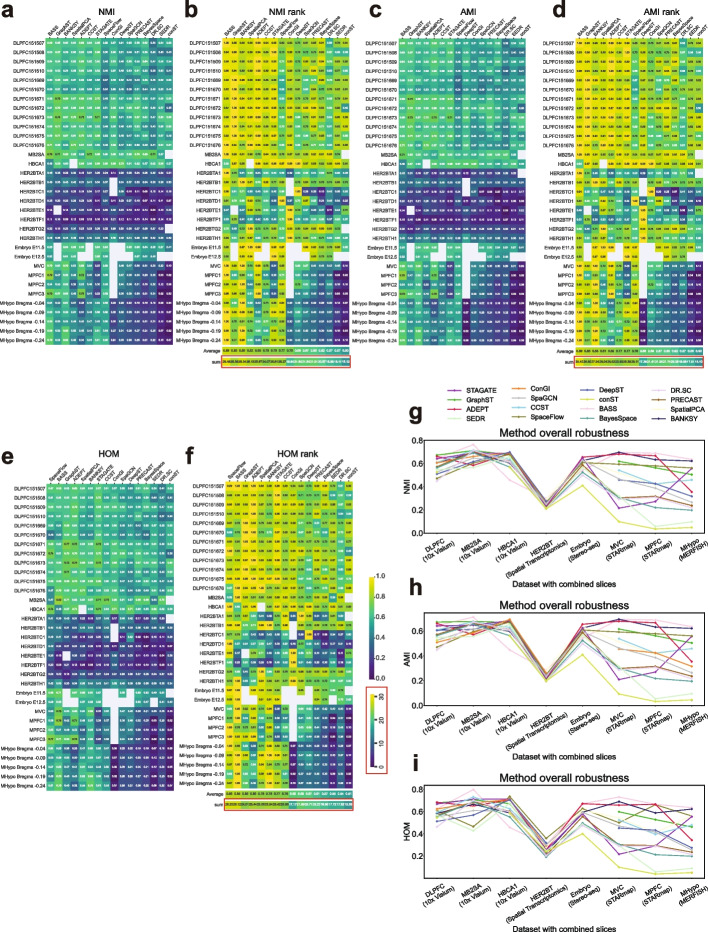
Clustering performance in terms of NMI, AMI, and HOM. **a** NMI heatmap. Each average NMI value is based on 20 runs. **b** Ranking heatmap. This ranking heatmap is created by normalizing all results within the same slice by dividing them by the maximum NMI value (representing the best performance) among all methods, thus standardizing all NMI values to 1. For each method, the best ranking for the sum result is 33, and the best ranking for the average result is 1. **c**, **d** Equivalent heatmaps as shown in (**a**, **b**) for AMI. **e**, **f** Equivalent heatmaps as shown in (**a**, **b**) for HOM. All heatmaps in (**a**–**f**) share a color bar ranging from 0 to 1. **g**–**i** Line plots illustrating the overall robustness of all methods across eight datasets in terms of NMI (**g**), AMI (**h**), and HOM (**i**)

We also utilized three additional metrics-Normalized Mutual Information (NMI), Adjusted Mutual Information (AMI), and Homogeneity (HOM)-to further evaluate the clustering performance of all 16 methods. Similar to the ARI evaluation, we plotted two heatmaps for each of these metrics. Details for computing the NMI, AMI, and HOM values, as well as each rank score, are provided in the “Methods” section. The ranking order using these metrics was highly consistent with that obtained using ARI, with only a few exceptions (Fig. 3a–f). BASS, GraphST, BANKSY, SpatialPCA, and ADEPT remained the top tools across the three metrics followed by CCST, and STAGATE, while SpaceFlow achieved the best HOM, indicating the highest cluster purity (Fig. 3e, f).

To investigate the overall robustness of each method, we aggregated the average values of these three metrics across slices within each dataset (Fig. 3g–i). The observed patterns were similar to those seen with ARI. BASS achieved the best performance in five out of eight datasets for these three metrics. STAGATE continued to perform well for the Stereo-seq Embryo dataset in terms of NMI and AMI. SpaceFlow and ConGI performed well for the MERFISH and tumor datasets, respectively.

### Qualitative and quantitative benchmarking of a Slide-seq v2 dataset

**Fig. 4:**
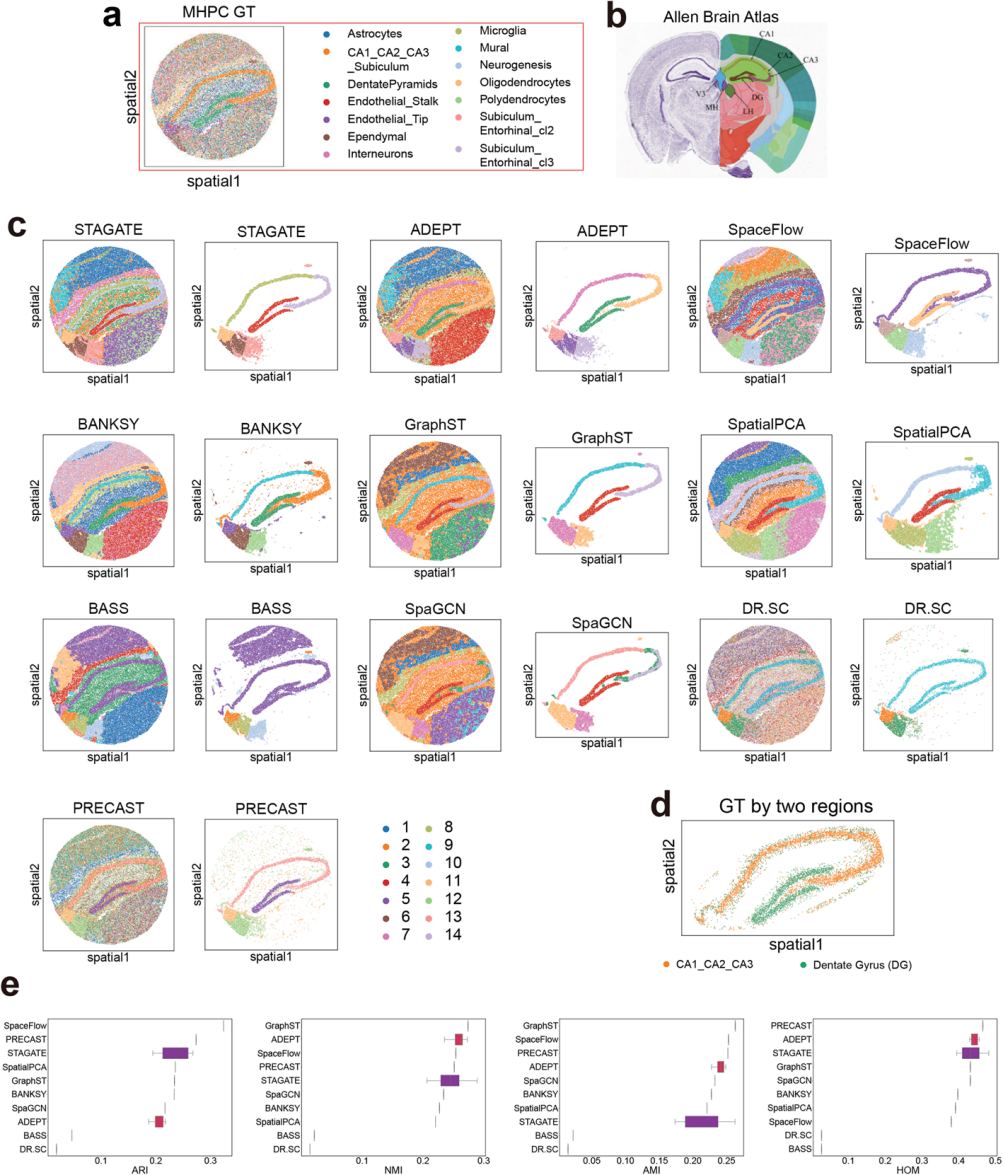
Clustering performance on the MHPC dataset. **a** Ground truth (GT) annotation for the MHPC dataset. **b** The Allen Brain Atlas. **c** Comparisons of the predicted clusters generated by different clustering methods. **d** Customized GT annotation only for CA1_CA2_CA3 and Dentate Gyrus for the MHPC dataset. **e** Box plots of ARI, NMI, AMI, and HOM for all tools based on customized GT annotation only for CA1_CA2_CA3 and Dentate Gyrus

So far, we quantitatively evaluated all clustering methods by ARI and other metrics. For the MHPC data using the Slide-seq v2 protocol (Fig. 4a), where the spots were labeled by cell types, visual comparison with the ground truth was more effective than calculating ARIs. Additionally, we employed the Allen Brain Atlas as a ground truth for the anatomical regions (Fig. 4b). The ground truth comprised four key distinguished anatomical regions, CA1, CA2, CA3, and dentate gyrus (DG), which displayed curved shapes. For better visualization, we have extracted clusters from each method to match these key distinguished anatomical regions. Our results demonstrated that all methods successfully recovered this feature; however, DR.SC and BASS failed to identify them as separate regions (Fig. 4c). Moreover, ADEPT, GraphST, STAGATE, and BANKSY could further differentiate CA1 and CA3 (Fig. 4c). Notably, no method delineated a separate CA2 region, merging it with CA3 instead. To quantitatively evaluate all methods for these regions, we conducted a manual region-based annotation of “CA1_CA2_CA3” and DG regions based on existing cell type annotations. This manual annotation (shown in Fig. 4d) served as the ground truth for calculating clustering performance, measured by ARI, NMI, AMI, and HOM. Our results indicated that PRECAST exhibited the highest overall performance across all four metrics, followed sequentially by GraphST, SpaceFlow, ADEPT, STAGATE, and BANKSY (Fig. 4e).

We further investigated three other key anatomical regions-third ventricle (V3), medial habenula (MH), and lateral habenula (LH). BASS, ADEPT, STAGATE, SpaceFlow, and BANKSY could successfully delineate these three regions. In conclusion, ADEPT, STAGATE, BANKSY, SpaceFlow, and GraphST were effective tools for delineating all seven key regions.

### Spatial continuity analysis for clustering methods

**Fig. 5:**
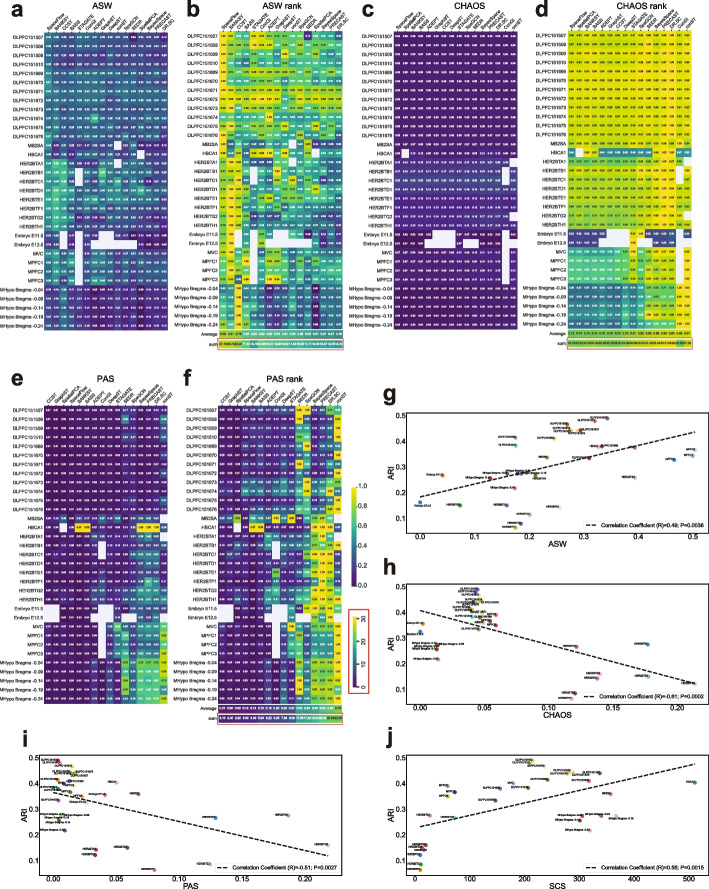
Clustering performance in terms of ASW, CHAOS, PAS, and SCS for spatial continuity. **a** ASW heatmap. Each average ASW value is based on 20 runs. **b** Ranking heatmap. This ranking heatmap is created by normalizing all results within the same slice by dividing them by the maximum ASW value (representing the best performance) among all methods, thus standardizing all ASW values to 1. A higher ASW value indicates greater spatial continuity. For each method, the best ranking for the sum result for ASW is 33, and the best ranking for the average result is 1. **c**, **d** Equivalent heatmaps as shown in **a**, **b** for CHAOS. **e**, **f** Equivalent heatmaps as shown in (**a**, **b**) for PAS. The ranking heatmaps were created by normalizing all results within the same slice by dividing them by the maximum CHAOS/PAS value (representing the worst performance) among all methods, thus standardizing all CHAOS/PAS values to 1. Lower CHAOS and PAS values indicate greater spatial continuity. For each method, the worst ranking for the sum result for CHAOS and PAS is 33, and the worst ranking for the average result is 1. All heatmaps in (**a**–**f**) share a color bar ranging from 0 to 1. **g**–**j** Average ARI values across all methods as a function of data slice complexity quantified by ASW (**g**), CHAOS (**h**), PAS (**i**), and SCS (**j**). Two Embryo slices were excluded in (**j**) for better visualization. Pearson correlation coefficients and *p*-values are indicated within the plots

Continuity is a key metric in spatial clustering, as it captures spatial coherence and well-defined interfaces between predicted spatial domains. To assess continuity by different methods, we utilized three widely recognized metrics: average silhouette width (ASW) [55], CHAOS [19], and percentage of abnormal spots (PAS) [19]. The methods are described in detail in the “Methods” section. Similar to the ARI evaluation, we plotted two heatmaps for each of these metrics. Details for computing the ASW, CHAOS, and PAS values, as well as each rank score, are provided in the “Methods” section. Unlike ASW, where a higher value indicates higher spatial continuity, lower CHAOS and PAS values indicate higher spatial continuity. Considering all three metrics together, we observed that SpaceFlow, BANKSY, and CCST achieved the best spatial continuity, followed by BASS and GraphST (Fig. 5a–f). SpatialPCA and ADEPT had similar overall rankings, with SpatialPCA demonstrating better spatial continuity in terms of CHAOS and PAS.

All clustering methods exhibited performance that varied considerably across datasets. To reveal the effect of data complexity on performance, we plotted the average ARI by all methods for each slice as a function of data complexity. (Fig. 5g–j). To quantify data complexity, we utilized ASW, CHAOS, and PAS as metrics to measure spatial continuity for each slice based on the ground truth labels. Additionally, we introduced another metric, the Spatial Coherence Score (SCS), to quantify data complexity. Details are described in the “Methods” section. The overall trend of the average ARI across all methods, represented by each regression line, indicated that clustering accuracy decreased as data complexity increased. All Pearson correlation values between ARI and each data complexity metric were significant (*p* = 0.0036, *p* = 0.0002, *p* = 0.0027, and *p* = 0.0015 for ASW, CHAOS, PAS, and SCS, respectively). Since a higher ASW and SCS value indicates higher spatial continuity and lower data complexity, their Pearson correlation coefficient was positive (*R* = 0.49 for ASW and *R* = 0.55 for SCS) in Fig. 5g and j. Conversely, higher CHAOS and PAS values indicate lower spatial continuity and higher data complexity, resulting in negative Pearson correlation coefficients (Fig. 5h, i; *R* = − 0.61 for CHAOS and *R* = − 0.51 for PAS). However, an intriguing observation emerged: the average ARIs for well-studied datasets were mostly above the regression line, whereas for less-studied datasets, average ARIs were below the regression line. This outcome indicated that the designs of most current algorithms favored the commonly used datasets and were not generally effective for all datasets. Though this phenomenon was due to the scarcity of available ST datasets with high-quality ground truth, it did exhibit a potential issue of algorithm overfitting, which should be noted and prevented in future studies.

### Runtime analysis for clustering methods

**Fig. 6:**
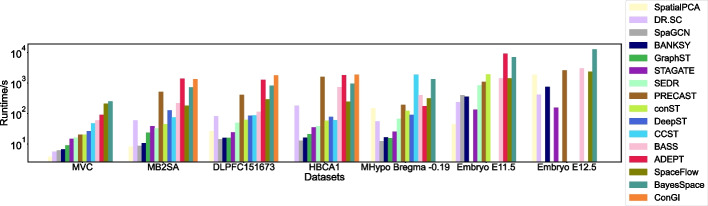
Runtime comparison of clustering methods. Runtime analysis of all 16 clustering methods on seven ST slices. The runtime is plotted by arranging the datasets in ascending order based on the number of spots and tools are sorted in ascending order based on the runtime of the first MVC dataset

Finally, we benchmarked the runtime of each method on seven selected ST slices (Fig. 6). The MVC slice has the smallest number of spots (1207). The MB2SA, DLPFC 151673, HBCA1, and MHypo Bregma -0.19 slices have 2695, 3611, 3798, and 5803 spots, respectively. The two largest Embryo slices have 30,124 and 51,365 spots, respectively. We plotted the runtime by arranging the datasets in ascending order based on the number of spots and sorted the tools in ascending order based on the runtime of the first MVC dataset. Overall, for the first five data slices, four tools-SpaGCN, BANKSY, GraphST, and STAGATE-demonstrated advantages in terms of runtime, as they could analyze each slice within a minute. Six tools, including SpatialPCA, DR.SC, SEDR, conST, DeepST, and SpaceFlow, exhibited comparably slower speeds but still completed execution within 5 mins per slice. In contrast, six tools-PRECAST, CCST, BASS, ADEPT, BayesSpace, and ConGI-lacked scalability and were significantly impacted by both the number of spots and genes, with their runtime increasing drastically as the data size grew. Regarding the two largest Embryo slices, STAGATE, BANKSY, and DR.SC demonstrated good scalability, processing both slices within 2–12 mins. SpaGCN and SEDR processed the Embryo E11.5 slice within 7–15 mins but could not process the Embryo E12.5 slice due to memory constraints on our computation platform, as described in the “Methods” section. conST, ADEPT, BASS, BayesSpace, PRECAST, SpaceFlow, and SpatialPCA could handle one or both slices, but their processing times increased significantly, ranging from 18 mins to 3.5 h. GraphST, DeepST, and CCST could not process either slice due to memory constraints. ConGI was also not applicable to either slice due to the absence of a histology image. Overall, STAGATE achieved the best runtime and scalability across all slices, followed by BANKSY and DR.SC.

### Assessing the characteristics of joint spot embedding with pairwise two-slice joint analysis

In contrast to the conventional approach of ST focusing on spatial domain distribution in a single slice, there is a growing recognition of the value of integrative and comparative analyses of ST datasets. In our pairwise two-slice joint analysis, we started by using nine pairs of DLPFC slices to explore whether integration could improve joint spot embeddings by leveraging adjacent consecutive slices. Evaluation experiments were conducted by introducing layer-wise alignment accuracy. The fundamental idea behind this analysis is based on the hypothesis that aligned spots across consecutive slices are more likely to belong to the same spatial domain or cell type. The detailed method for defining layer-wise alignment accuracy is outlined in the “Methods” section.

**Fig. 7:**
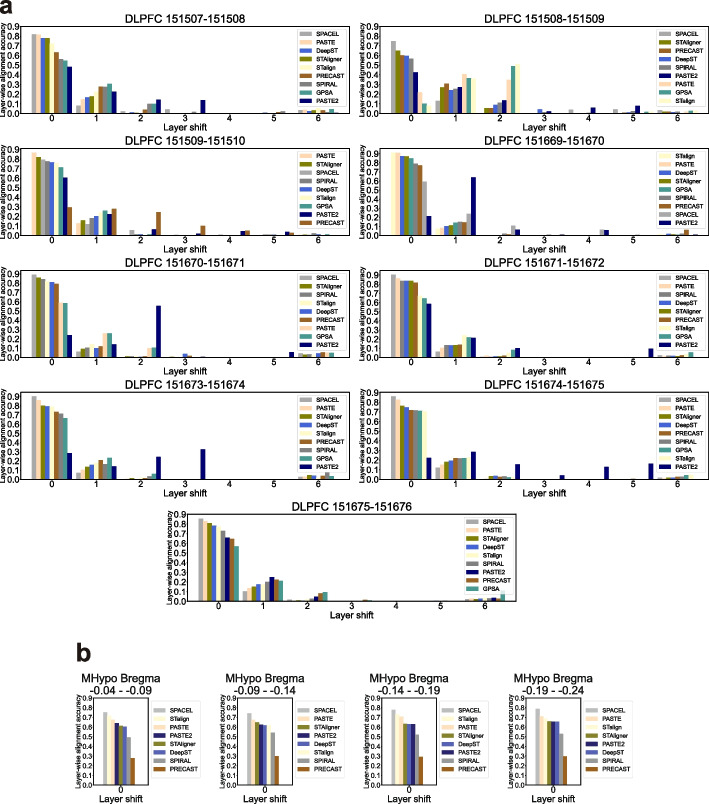
Bar plots for layer-wise alignment accuracy. **a** Bar plots depicting the layer-wise alignment accuracy for a layer shift from 0 to 6 for different methods on nine DLPFC slice pairs. **b** Bar plots depicting the layer-wise alignment accuracy for a layer shift of 0 for different methods on four MHypo slice pairs. GPSA could not be applied to the MHypo dataset. Tools are sorted in descending order based on the accuracy for layer shift of 0 in (**a**, **b**)

In Fig. 7a, we compared the layer-wise alignment accuracy of all nine methods on nine DLPFC slice pairs. Given the unique layered structure of DLPFC data, we designed this evaluation metric to assess whether “anchor” spots from the first slice and “aligned” spots from the second slice belong to the same layer (layer shift = 0) or different layers (layer shift = 1 to 6). The expectation was that a good integration or alignment tool would show high accuracy for anchor and aligned spots belonging to the same layer (layer shift = 0), and this accuracy should decrease when the number of layer shift increases. We plotted the layer-wise alignment accuracy and sorted the tools in descending order based on the accuracy for layer shift of 0. In seven out of nine DLPFC slice pairs, SPACEL demonstrated the highest layer-wise alignment accuracy, while PASTE and STalign led in the remaining two pairs (Fig. 7a). A similar experiment was conducted on four pairs drawn from the MHypo dataset (Fig. 7b), but layer-wise alignment accuracy was only plotted for a layer shift of 0 due to the nature of the data. SPACEL still exhibited the best performance, followed by PASTE and STalign in the second position. It was not surprising that the two alignment tools, SPACEL and PASTE, exhibited the highest accuracy in layer-wise alignment across most pairs, which was expected as their primary objective was the direct alignment of spots across slices, rather than relying on joint spot embeddings for integration analysis. Conversely, tools like STAligner, PRECAST, DeepST, and SPIRAL, which leverage joint spot embeddings for indirect alignment across slices, demonstrated slightly lower but still satisfactory layer-wise alignment accuracy. Among these tools, STAligner achieved the highest accuracy, followed by DeepST, while PRECAST and SPRIAL performed the least accurately. These results highlighted, to some extent, the inherent qualities of joint spot embeddings by these integration tools. PASTE2, an extension version of PASTE, exhibited poor performance in this scenario because it primarily addresses the partial overlap alignment problem, where only partial overlap occurs between two slices or slice-specific cell types. Notably, the other two alignment tools, STalign and GPSA, lacked in robust and accurate alignment performance compared to SPACEL and PASTE.

**Fig. 8:**
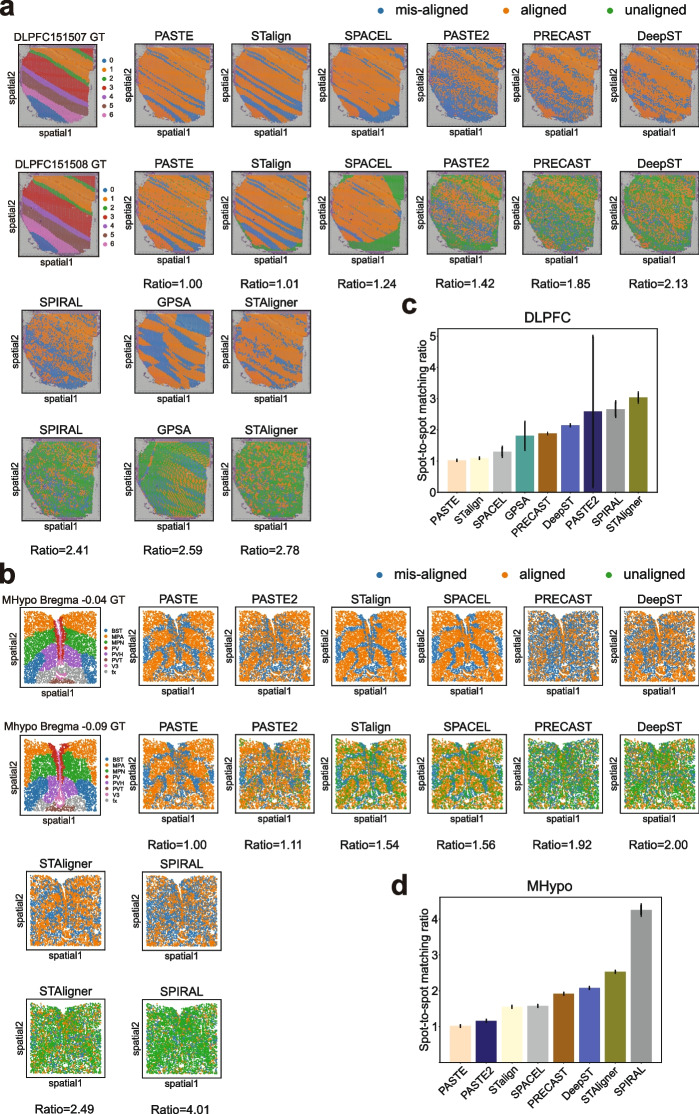
Visualization plots for alignment-misalignment-unalignment and spot-to-spot mapping ratio. **a**, **b** Visualization plots showing aligned spots, misaligned spots, and unaligned spots when aligning the anchor spot from the first (top) slice to the aligned spots on the second (bottom) slice on DLPFC 151507-151508 (**a**) and MHypo Bregma -0.04 - -0.09 pair (**b**). Values below each plot represent the spot-to-spot matching ratio. **c**, **d** Bar plots representing the average spot-to-spot mapping ratio of each tool on two datasets: DLPFC (**c**) and MHypo (**d**). GPSA could not be applied to the MHypo dataset

While layer-wise alignment accuracy provides insight into spot-to-layer alignment, it is crucial to evaluate the spot-to-spot matching ratio to further evaluate joint spot embeddings. In Fig. 8a, b, we marked “anchor” and “aligned” spots on both slices using three different colors, further classifying them into aligned (orange), misaligned (blue), and unaligned (green) spots based on ground truth layer labels, as described in the “Methods” section. Notably, for the DLPFC 151507-151508 pair, STAligner, GPSA, SPIRAL, DeepST, PASTE2, and PRECAST showed a notable proportion of unaligned spots on the second slice. This suggested a bias in these six tools, aligning multiple “anchor” spots from the first slice to the same “aligned” spot on the second slice, thereby leaving a significant number of spots unaligned on the second slice. The spot-to-spot mapping ratio further corroborated this observation, with PASTE demonstrating the lowest ratio (1.00), followed by STalign (1.01), SPACEL (1.24), PASTE2 (1.42), PRECAST (1.85), DeepST (2.13), SPIRAL (2.41), GPSA (2.59), and STAligner (2.78). Averaging this ratio across all nine pairs for each tool revealed a similar pattern (Fig. 8c), except that GPSA achieved a better overall ratio, while PASTE2 had a worse overall ratio. Moreover, across all nine pairs, it was observed that misaligned spots (Fig. 8a and Additional file 2: Fig. S9-S12) on the first slice tended to aggregate along the layer boundaries in PASTE, STalign, and SPACEL. In contrast, the other rest tools exhibited a dispersion of these misaligned spots within the layers. The high spot-to-spot mapping ratio and the dispersed pattern of misaligned spots in all integration tools suggested a shared trade-off, wherein the learned low-dimensional embeddings sacrifice certain local geometric information in the process of optimization and training. SPACEL, the alignment tool, exhibited coherent regions of unaligned spots (illustrated in green) outside the matched regions.

We further performed this evaluation analysis in four pairs of MHypo slices and observed a similar trend for spot-to-spot mapping ratio and a similar dispersed pattern of misaligned spots in all tools (Fig. 8b and Additional file 2: Fig. S13-S14). Specifically, SPIRAL had the worst average spot-to-spot mapping ratio, followed by STAligner, DeepST, and PRECAST (Fig. 8d). PASTE2 and PASTE achieved a ratio of approximately 1. STalign and SPACEL demonstrated a less favorable average ratio (1.55 for STalign; 1.58 for SPACEL) for the MHypo data in comparison to the DLPFC data (1.09 for STalign; 1.30 for SPACEL).

### Alignment accuracy on simulated datasets

While real datasets enabled us to assess alignment accuracy to some extent, they lacked precise spot-to-spot alignment ground truth. To comprehensively investigate alignment accuracy, we simulated datasets with the gold standard for different scenarios to demonstrate the robustness of all alignment and integration methods.

**Fig. 9:**
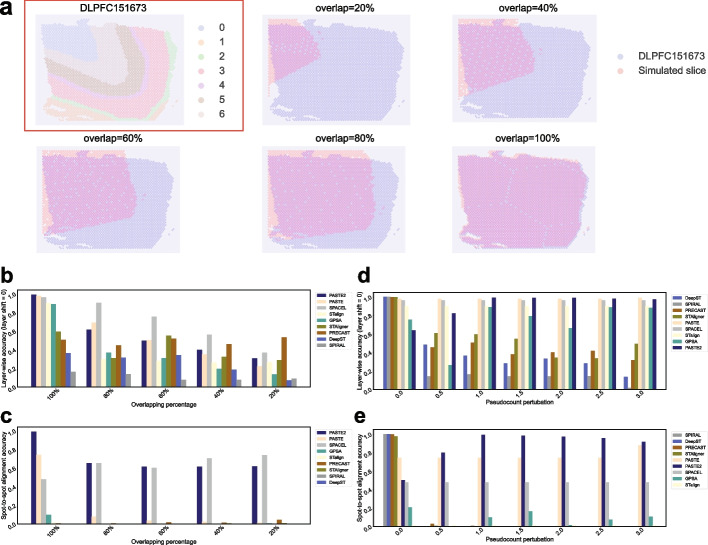
Alignment accuracy in simulation Data. **a** DLPFC 151673 slice, consisting of seven layers, along with its simulated consecutive slices featuring overlapping ratios of 20%, 40%, 60%, 80%, and 100% with respect to DLPFC 151673 slice. **b** Layer-wise alignment accuracy for a layer shift of 0 across different tools, as a function of increased overlapping ratio. Tools are sorted in descending order based on the layer-wise alignment accuracy for layer shift of 0 on the left dataset (with 100% overlapping percentage). **c** Spot-to-spot alignment accuracy across different tools as a function of increased overlapping ratio. Tools are sorted in descending order based on the spot-to-spot alignment accuracy on the left dataset (with 100% overlapping percentage). **d** Layer-wise alignment accuracy for a layer shift of 0 across different tools, as a function of increased pseudocount perturbation. Tools are sorted in descending order based on the layer-wise alignment accuracy for layer shift of 0 on the left dataset (with pseudocount perturbation = 0.0). **e** Spot-to-spot alignment accuracy across different tools as a function of increased pseudocount perturbation. Tools are sorted in descending order based on the spot-to-spot alignment accuracy on the left dataset (with pseudocount perturbation = 0.0)

We first used one DLPFC slice as the reference and simulated another slice with different overlap ratios (20%, 40%, 60%, 80%, and 100%) in comparison to the reference slice (Fig. 9a). In this simulation scenario, the pseudocount (gene expression) perturbation was fixed at 1.0 for all simulated slices. The detailed simulation method is outlined in the “Methods” section. In Fig. 9b, c, the layer-wise alignment accuracy for a layer shift of 0 and spot-to-spot alignment accuracy are shown in bar plots. We observed that all five alignment methods achieved superior layer-wise alignment accuracy for a layer shift of 0 in comparison to the four integration methods. Furthermore, for each tool, accuracy tended to decline as the overlapping ratio between two slices diminished. Nevertheless, in terms of spot-to-spot alignment accuracy, all four integration methods-STAligner, PRECAST, DeepST, and SPIRAL-failed to achieve even a marginal value, which was consistent with the earlier conclusion that these tools exhibit relatively high spot-to-spot mapping ratios. On the other hand, three alignment tools-SPACEL, PASTE2, and PASTE-achieved relatively better spot-to-spot alignment accuracy. Among them, PASTE2 achieved a near-perfect accuracy at the 100% overlapping ratio and consistently maintained approximately 60% accuracy at lower overlapping ratios. SPACEL exhibited slightly better accuracy than PASTE2 when the overlapping ratio was lower than 100%. However, its accuracy decreased to approximately 40% at the 100% overlapping ratio. PASTE, on the other hand, failed to achieve satisfactory accuracy when the overlapping ratio was lower than 100%. For the other two alignment tools, STalign and GPSA, the spot-to-spot alignment accuracy was unexpectedly low, comparable to that of the four integration tools.

In the second simulation scenario, we simulated the slice with different pseudocounts (0–3.0 with a step size of 0.5) to represent perturbation on gene expression while keeping the overlapping ratio fixed at 100%. In Fig. 9d, the bar plots demonstrated that the layer-wise alignment accuracy for a layer shift of 0 of four integration tools−DeepST, SPIRAL, PRECAST, and STAligner−decreased when pseudocount perturbation increased. This result suggested that all integration methods were sensitive to perturbation on the expression profiles, as they utilized gene expression profiles as spot (node) features when constructing a graph model for training. Conversely, five alignment tools-PASTE, SPACEL, STalign, GPSA, and PASTE2-exhibited significantly greater resilience to perturbations in gene expression. This resilience stems from their objective functions for alignment, which allowed for a more pronounced emphasis on spatial coordinates when gene expression varied across slices. Regarding spot-to-spot alignment accuracy in Fig. 9e, three alignment tools (PASTE, PASTE2, and SPACEL) consistently maintained similar accuracy across various pseudocount perturbations. PASTE2 demonstrated the highest accuracy when pseudocount perturbation ranged from 0.5 to 3.0. The other two alignment tools (STalign and GPSA) still demonstrated low spot-to-spot alignment accuracy across all scenarios. Notably, when pseudocount perturbation was set to 0, indicating identical gene expression levels for each spot across slices, all four integration tools achieved better accuracy.

### Integration methods improve integration of consecutive slices with batch correction

Once joint spot embeddings for each integration method were generated, we further visually evaluated the “batch-corrected” joint embeddings for the integration of consecutive slices using two components from uniform manifold approximation (UMAP). Alignment tools, PASTE, PASTE2, and SPACEL, were excluded from this analysis as they did not generate latent embeddings.

**Fig. 10:**
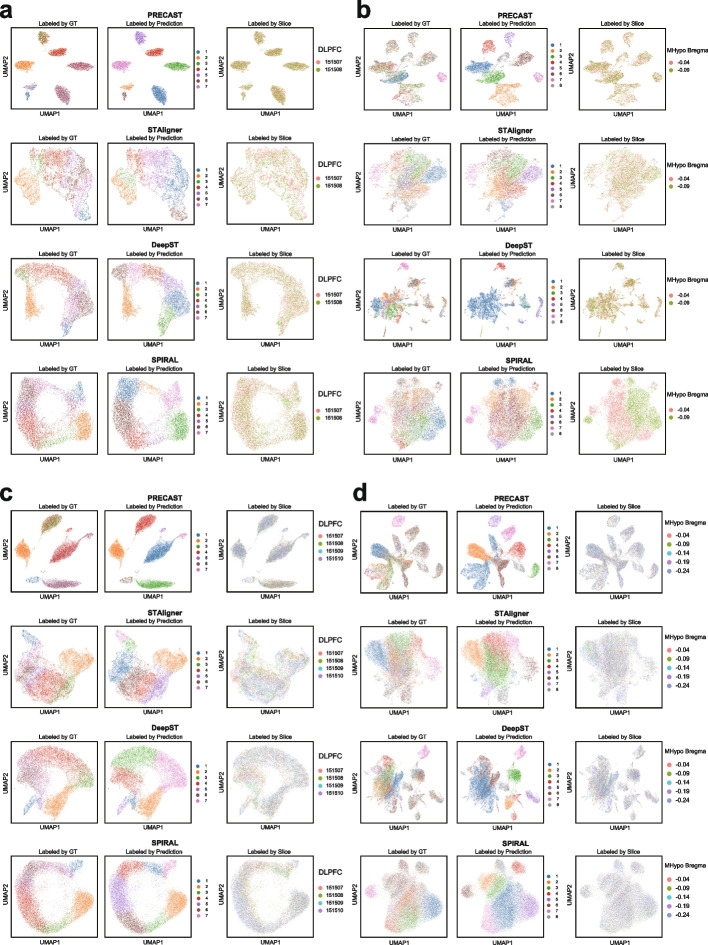
UMAP plots of low dimensional joint embedding distribution for batch correction. **a**–**d** These UMAP plots depicting the 2D distribution of latent joint embeddings after integration with batch correction by different methods on the DLPFC 151507-151508 pair (**a**), the MHypo Bregma -0.04 - -0.09 pair (**b**), the DLPFC 151507-151510 four consecutive slices (**c**), and the MHypo Bregma -0.04 - -0.24 five consecutive slices. Each UMAP contains colored spots labeled by three different setups: ground truth (GT), method prediction, and slice index

For the DLPFC 151507 and 151508 pair (Fig. 10a), the UMAP plots for PRECAST, STAligner, DeepST, and SPIRAL showed that spots from two different slices were evenly mixed to some extent (Fig. 10a, right panel), and their predicted domain clusters were well segregated (Fig. 10, middle panel). Specifically, PRECAST tended to generate embeddings in a pattern with separated clusters, with some predicted clusters encompassing spots from different domains, a pattern that did not entirely align with the ground truth (Fig. 10a, left panel). STAligner, DeepST, and SPIRAL maintained the hierarchical connections of the seven layers in the latent embedding space to some degree. However, there were instances where predicted spatial domains included spots from nearby domains, or one spatial domain was predicted to be two adjacent domains. STAligner achieved better UMAP visualization than DeepST and SPIRAL. Among all tools, PRECAST lost more geometry information than the other three tools since it prominently separated spatial domains in the latent space. We further demonstrated this UMAP analysis for all the rest DLPFC pairs and plotted annotations by ground truth, method prediction, and slice index (Additional file 2: Fig. S15-S16). All remaining UMAP results exhibited consistent patterns and further affirmed that all four methods were capable of generating “batch-corrected” joint embeddings for the integration of consecutive slices. However, the integrated spatial domains were not highly concordant with the ground truth.

We extended this analysis to four pairs of the MHypo data (Fig. 10b and Additional file 2: Fig. S17). The joint embeddings generated by PRECAST, STAligner, and DeepST somewhat facilitated integration across consecutive slices, although this effect was much inferior compared to the results of the DLPFC data. These three tools exhibited several connected small clusters or a single large cluster which were hard to differentiate based on the annotation by ground truth. The other tool, SPIRAL, experienced a significant batch effect as its joint embeddings across slices were unevenly mixed and experienced substantial separation. This result was in agreement with the least favorable spot-to-spot mapping ratio (4.01) by SPRIAL.

In addition to benchmarking on the integration of slice pairs, we further demonstrated the performance of each method on multi-slice ($>2$) integration. All UMAP plots for PRECAST, STAligner, DeepST, and SPIRAL indicated a relatively even mixture of spots from four distinct slices provided by three samples (DLPFC 151507-151510, 151669-151672, 151673-151676) (Fig. 10c and Additional file 2: Fig. S18). Consistent with observations in paired settings, the embeddings generated by PRECAST continued to exhibit a pattern characterized by separated clusters. On the other hand, STAligner, DeepST, and SPIRAL still maintained hierarchical connections across seven layers in the latent embedding space. STAligner demonstrated slightly better UMAP visualization than DeepST and SPIRAL. As for the integration of the five slices of the MHypo dataset (Fig. 10d), all tools still displayed several small connected clusters or a single large cluster that was challenging to differentiate based on the annotation by ground truth. However, SPIRAL mixed the spots across five slices evenly and did not display any batch effect, which indicated SPIRAL could use adequate data to remove the batch effect for its latent embeddings. In summary, there is still a need for an optimal and robust tool for integration. While existing tools have shown efficacy to some extent in well-studied datasets, their performance has not consistently generalized to diverse datasets.

### Integration methods enhance domain identification through joint embedding

Integrating data from multiple ST slices can allow us to estimate joint embeddings of expressions representing variations between cell or domain types across slices, which has the potential to better detect spatial domains or cell types, compared to single slice analysis [33]. To further quantitatively compare the effectiveness of these methods in capturing spatial domains via joint embeddings, we employed joint embeddings from each pair of slices in the MHypo and DLPFC datasets to perform clustering together using the clustering method mclust [56]. We then computed ARI as an evaluation metric to compare the clustering results of each tool with the ground truth in each slice, with higher ARI scores indicating better domain identification.

**Fig. 11:**
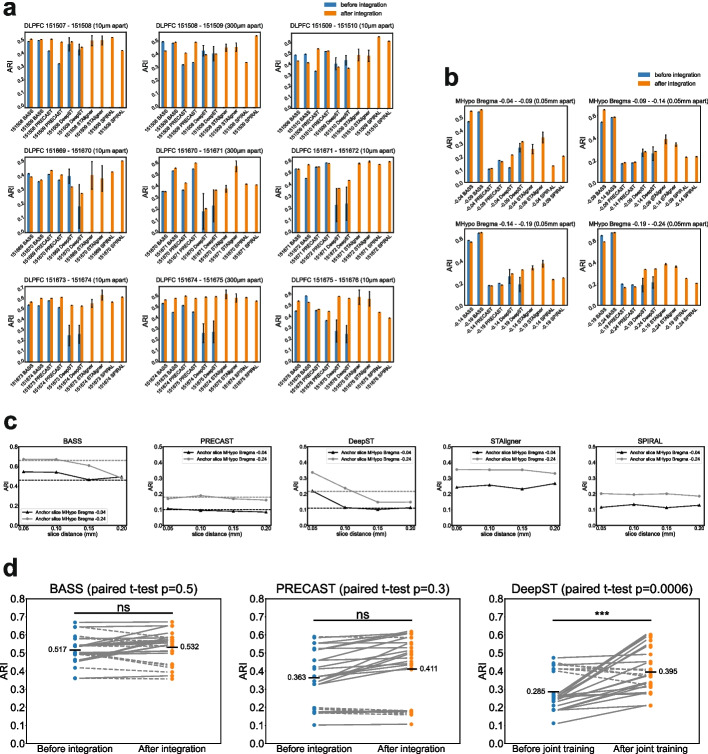
ARI plots before and after integration for domain identification in DLPFC and MHypo datasets. **a**, **b** ARI bar plots for nine DLPFC pairs (**a**) and four MHypo pairs (**b**) using different methods. Blue bars represent the average ARI values for 20 runs before integration (in single-slice mode), and orange bars represent the average ARI values for 20 runs after integration. Error bars represent standard deviations calculated from 20 runs. Note that the blue bars for STAligner and SPIRAL remain unpopulated since they do not support single-slice clustering. **c** ARI plots for anchor slices as a function of increased slice distance for different methods. Dashed lines indicate the ARI of anchor slices before integration (in single-slice mode). **d** Paired ARI plots comparing values before and after integration for three methods. Solid lines indicate that ARI after integration is higher than before integration. Dashed lines indicate that ARI after integration is lower than before integration. Statistical significance between the before and after integration values is assessed using a paired *t*-test and indicated as follows: $$^{ns}p \ge 0.05$$ and $$^{***}p<0.001$$. The average ARI across before and after integration conditions is marked with a bar and the respective value

In Fig. 11a, b, we plotted the average ARI results under two scenarios. BASS, PRECAST, and DeepST supported both single-slice and multi-slice joint (integration) analyses. Accordingly, we utilized blue bars to depict the results before integration (single-slice mode) and orange bars to represent the results after integration. However, since STAligner and SPIRAL only have a multi-slice joint analysis mode, the blue bars for these methods were left unpopulated. It was difficult to conclude which tool had the overall best performance in all pairs after integration. In nine pairs of DLPFC data (Fig. 11a), DeepST and STAligner exhibited the most variance across all runs. SPIRAL demonstrated the best performance on DLPFC 151509-151510 and 151669-151670 pairs. STAligner led the performance on DLPFC 151673-151674, 151674-151675, and 151675-151676 pairs, albeit marginally. Notably, the DLPFC 151670-151671 pair, characterized by a large spatial distance along the *z*-axis (300 µm apart) within the tissue between two slices, presented challenges for all methods. These tools either exhibited a significant performance discrepancy in two slices or failed to perform well in both slices. A similar observation has been spotted on the 151508-151509 distant pair as well. In the DLPFC 151671-151672 pair, SPIRAL and STAligner demonstrated better performance. Most methods performed similarly on the DLPFC 151507-151508 pair. Results were comparatively simpler on the four pairs of the MHypo dataset (Fig. 11b). BASS demonstrated superior performance in all four pairs, followed by STAligner. However, the remaining three methods failed to produce reasonable results. To compute an overall ranking based on ARI for each tool across all slice pairs from the DLPFC and MHypo datasets, we generated ARI value and rank heatmaps after integration. Our results demonstrated that BASS achieved the best average and sum rank after integration, followed by STAligner and SPIRAL (Additional file 2: Fig. S19).

We investigated every adjacent consecutive slice pair before and after integration analysis. The distant DLPFC slice pairs such as 151670-151671 and 151508-151509 posed challenges for all methods to improve clustering accuracy after integration. To explore how the physical distance between slices affects integration, we analyzed the ARI of all tools at four distances from the Bregma in the MHypo dataset. Specifically, we examined distances of 0.05 mm, 0.1 mm, 0.15 mm, and 0.2 mm, using slices at Bregma -0.04 and -0.24 as fixed anchor points. This analysis included comparisons across seven distinct pairings from Bregma -0.04 to -0.24, helping to discern the impact of slice distance on integration effectiveness. We plotted the ARI of two anchor slices against the increasing distance between slices, observing two different outcomes (Fig. 11c): (1) for BASS and DeepST, integration led to an improvement in the ARI of both anchor slices (surpassing the dashed line that represents the ARI for a single anchor slice before integration) when the distance between the slices was small. However, the ARI of the anchor slices declined as the distance between the slices increased. This indicated that integration could reduce the clustering accuracy of the anchor slice if the slice distance was sufficiently large (dropping below the corresponding dashed line). (2) For PRECAST, STAligner, and SPRIAL, integrating with slices that were either close or distant did not impact the clustering accuracy of the anchor slices. In conclusion, integration can enhance clustering for individual slices, but the effectiveness of this improvement depends on the distance between slices for each specific dataset.

Although no clear overall winner emerged after integration, integration analysis produced some improvement in clustering accuracy compared to single-slice analysis within certain tools. Specifically, both PRECAST and DeepST exhibited enhanced clustering accuracy after integration (Fig. 11d). Across a total of 26 before-and-after pair conditions for two datasets, PRECAST’s average ARI increased from 0.363 before integration to 0.411 after, though this change was not statistically significant (*p *= 0.3). In contrast, DeepST exhibited a notable increase in clustering accuracy, with the average ARI improving from 0.285 before integration to 0.395 after, which was statistically significant (*p *= 0.0006). BASS did not show any significant improvement in clustering accuracy through integration, with its average ARI slightly changing from 0.517 before to 0.532 after integration (*p *= 0.5).

### Integration methods align samples across different anatomical regions and development stages

**Fig. 12:**
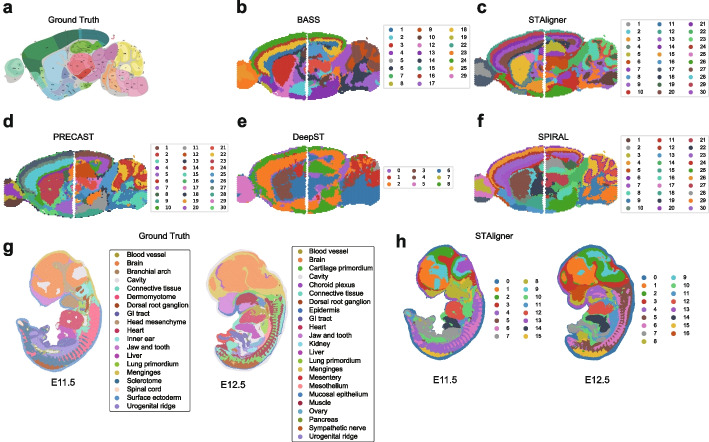
Visualization plots for integration with batch correction in MB2SA&P dataset and mouse Embryo dataset. **a** The Allen Brain atlas serving as the ground truth. **b**–**f** Domain identification by five methods in the MB2SA&P dataset. **g** Domain identification by the ground truth in the mouse Embryo dataset. **h** Domain identification by STAligner in the mouse Embryo dataset

So far, our benchmarking has focused on evaluating the integration capabilities of methods across adjacent consecutive sample slices. In this section, we delved deeper into its efficacy for integrating non-consecutive slices. We employed a 10x Visium dataset representing mouse brain sagittal sections, divided into posterior and anterior. We employed the Allen Brain Atlas as a reference (Fig. 12a) and visually compared the clustering results of all methods (Fig. 12b–f). Among all methods, PRECAST demonstrated the least effective performance and failed to detect and connect common spatial domains. In contrast, BASS, STAligner, DeepST, and SPIRAL were better able to identify and connect common spatial domains along this shared boundary. Specifically, only STAligner identified and aligned six distinct layers in the cerebral cortex (CTX) across the anterior and posterior sections. On the other hand, BASS and SPRIAL only managed to identify four distinct layers in CTX. Additionally, STAligner and SPRIAL performed well in distinguishing layers within the cerebellar cortex (CBX). However, none of them identified a coherent arc across two sections for CA1, CA2, and CA3. In summary, STAligner showed capacity in integration for adjacent slices across different anatomical regions.

Next, we investigated the ability of all methods to integrate two slices from different development stages, to study the spatiotemporal development in tissue structures during mouse organogenesis. Only STAligner has scalability in processing this big benchmarking dataset (over 50k spots for each slice), so other tools were excluded from this analysis. In Fig. 12g, the two mouse embryo slices were acquired at two different time points (E11.5 and E12.5) with region-based manual annotations for different organs and tissues. We observed that STAligner successfully retrieved several shared structures such as dorsal root ganglion, brain, heart, and liver in both slices (Fig. 12h). We also observed that at developmental stage E11.5, structures like the ovary and kidney were less developed compared to E12.5. These results facilitated the reconstruction of the developmental progression of each tissue structure throughout organogenesis.

### Reconstruction of 3D architecture from consecutive 2D slices

**Fig. 13:**
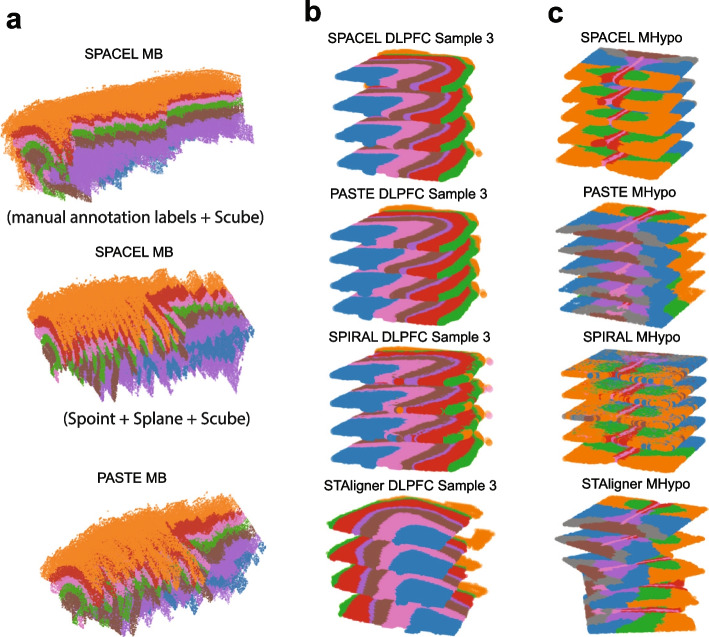
Reconstruction of 3D architecture of three different datasets. **a** 3D architecture reconstructed from 33 slices of MB data using SPACEL (with and without manual annotation labels) and PASTE. **b** 3D architecture reconstructed from four slices (DLPFC 151673-151676) of DLPFC Sample 3 using SPACEL, PASTE, SPIRAL, and STAligner. **c** 3D architecture reconstructed from five slices of MHypo data using SPACEL, PASTE, SPIRAL, and STAligner

Initially, 2D slices were produced from 3D tissue, and alignment or integration tools, specifically designed for pairwise or all-to-all alignments using multiple adjacent consecutive slices, can then reconstruct the 3D architecture. 3D architecture allows users to explore the dynamics of transcript distributions from any direction, so reconstructing an effective 3D architecture of complex tissues or organs is essential. In Fig. 13, we provided 3D reconstruction visualization results from three different samples using four methods, SPACEL, PASTE, SPIRAL, and STAligner. The methods are described in detail in the “Methods” section. All four tools achieved consistent and satisfactory 3D visualization results on DLPFC sample 3, encompassing four adjacent consecutive slices numbered 151673-151674-151675-151676 (Fig. 13b). For the MHypo sample which contains five consecutive slices, SPACEL and PASTE demonstrated comparable and effective 3D visualizations (Fig. 13c). In contrast, SPIRAL exhibited misaligned scatter spots beginning from the second slice, and the occurrence of these misalignments increased with the addition of more stacks of slices. Starting from the third slice, STAligner exhibited rotational distortions in the slices, leading to a discordant 3D architecture. The underlying reason could be that SPIRAL performed all-to-all alignments, whereas SPACEL and PASTE performed pairwise alignments between each pair of adjacent consecutive slices sequentially. All-to-all alignments have the potential to introduce more false alignment, particularly when two slices are not closely positioned along the *z*-axis. GPSA can reconstruct the 3D architecture using DLPFC slices; however, the original shape of the DLPFC slice is distorted after alignment (Additional file 2: Fig. S20).

In terms of the MB sample, which contains 33 adjacent consecutive mouse brain tissue (Fig. 13a), only SPACEL and PASTE proved suitable for reconstructing the 3D architecture with this substantial number of slices. We selected a similar orientation of the 3D architecture for comparison purposes. The final module, Scube in SPACEL, successfully generated an effective 3D visualization by incorporating manual annotation labels. However, both SPACEL (without manual annotation labels) and PASTE produced a discordant 3D architecture, particularly noticeable from the second half of the slices onward. Combining pairwise alignments from multiple adjacent slices into a stacked 3D alignment of tissue led to the propagation of errors, resulting in the observation of two disjointed 3D architectures.

### Runtime analysis for alignment and integration methods

**Fig. 14:**
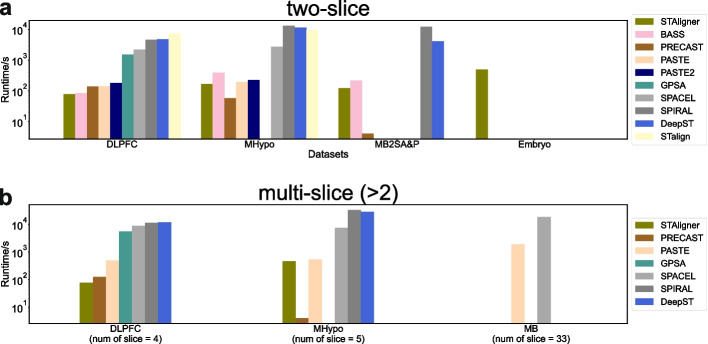
Comparison of runtime bar plots for different integration methods across five datasets. **a** Runtime for aligning or integrating two slices across four datasets. **b** Runtime for aligning or integrating multiple (> 2) slices across three datasets. Empty columns for specific tools indicate scenarios where either the tool is not optimized for such cases, or where the memory consumption is excessively high, resulting in the tool’s inability to complete execution

Finally, we benchmarked the average runtime of each alignment and integration method on five selected datasets (Fig. 14). The DLPFC and the MB2SA&P datasets were medium-sized, with approximately 3-4k spots and 30k genes. Though each slice of the MHypo dataset has approximately 5k spots, each spot only contains 155 genes. The Embryo dataset is the largest in terms of the number of spots and genes. Lastly, the MB dataset has 33 slices in total for alignment and 3D reconstruction. We plotted the runtime and sorted the tools in ascending order based on the runtime of the first DLPFC dataset. The plot of Fig. 14a illustrates the average runtime when aligning or integrating two slices. Empty columns indicate scenarios where either the algorithm is not optimized for such use cases, or where memory consumption is excessively high, leading to the tool’s inability to complete execution. Overall, methods such as STAligner, BASS, PRECAST, PASTE, and PASTE2 finished integration within 10 mins and exhibited reasonable scalability. Their time consumption was only marginally affected by increases in both the number of spots and genes. In contrast, scalability issues were more pronounced with methods like GPSA, SPACEL, SPIRAL, DeepST, and STalign, where integration tasks might take hours or even days to complete. STAligner stands out as the sole tool capable of completing analysis on the Embryo dataset without encountering any memory constraints thus far.

In Fig. 14b, we further compared the runtime of each tool when aligning or integrating multiple (> 2) slices. STAligner, PRECAST, and PASTE continued to exhibit promising scalability under these conditions. GPSA, SPACEL, SPIRAL, and DeepST showed significantly slower performance, typically being 100x to 1000x slower than the aforementioned methods when integrating more than two slices. PASTE and SPACEL took 32 mins and 5 h, respectively, to complete 3D alignment and reconstruction for the MB dataset.

## Discussion

In this study, we conducted comprehensive benchmark analyses covering different clustering, alignment, and integration tasks. We assessed 16 clustering methods, five alignment methods, and five integration methods across 68 slices of 10 publicly available ST datasets. We provide a user recommendation table (Table 2) for users to choose an optimal tool to conduct the corresponding analysis. For the majority of our recommendations, we based our conclusions on overall rankings derived from multiple metrics and various datasets. Our study revealed that BASS, GraphST, BANKSY, ADEPT, SpatialPCA, STAGATE, and CCST outperformed the other ten clustering methods in terms of overall clustering accuracy, robustness, and continuity, as evaluated by seven metrics: ARI, NMI, AMI, HOM, ASW, CHAOS, and PAS. Despite these findings, identifying a definitive best-performing tool was challenging. For example, while BASS achieved the best overall accuracy, it did not excel in clustering continuity. Additionally, certain other tools exhibited their peak performance within specific ST protocols or tissue types. Notably, the overall performance trend for all methods decreased as the data complexity increased. All methods potentially suffer from algorithm overfitting, as indicated by their performance exceeding expectations on well-studied datasets but underperforming on less-studied ones. In terms of runtime and scalability, STAGATE, BANKSY, DR.SC, SpatialPCA, SpaceFlow, and PRECAST demonstrated the best scalability across datasets of varying sizes.

**Table 2:** User recommendation table

Analysis	Top 1	Top 2	Top 3	Top 4	Top 5
Clustering accuracy (Generic)	BASS	GraphST	BANKSY/ADEPT	SpatialPCA	STAGATE/CCST
Clustering accuracy (Tumor tissue)	ConGI	DeepST	SpatialPCA	BASS	SpaceFlow
Clustering accuracy (Brain tissue)	BASS	BANKSY	STAGATE	ADEPT/GraphST/SpatialPCA	-
Clustering accuracy (10x Visium)	GraphST	ADEPT	STAGATE	BASS	-
Clustering accuracy (Spatial Transcriptomics)	ConGI	DeepST/SpatialPCA	BASS	SpaceFlow	-
Clustering accuracy (Stereo-seq)	STAGATE	SpatialPCA	BANKSY	SpaceFLow	PRECAST
Clustering accuracy (STARmap)	BASS	ADEPT	BANKSY	SpaceFlow	GraphST
Clustering accuracy (MERFISH)	BASS	BANKSY	SpaceFlow	STAGATE	GraphST
Clustering accuracy (Slide-seq v2)	ADEPT/STAGATE/BANKSY	SpaceFlow/GraphST	-	-	-
Clustering robustness (across various data)	BASS	BANKSY	GraphST/ADEPT	SpatialPCA	-
Clustering continuity	SpaceFlow	CCST	BANKSY	BASS	GraphST
Clustering runtime and scalability	STAGATE	BANKSY	DR.SC	SpatialPCA	SpaceFlow/PRECAST
Integration (layer-wise alignment accuracy)	SPACEL	PASTE	STAligner	DeepST	SPIRAL
Integration (spot-to-spot mapping ratio)	PASTE	STalign	SPACEL	PRECAST	-
Integration on simulated data (spot-to-spot alignment accuracy)	PASTE2	PASTE	SPACEL	-	-
Integration with batch correction (by joint embeddings)	STAligner	DeepST	PRECAST	-	-
Clustering accuracy (by joint embeddings)	BASS	STAligner	-	-	-
Integration (across conditions)	STAligner	BASS	-	-	-
3D reconstruction	SPACEL	PASTE	-	-	-
Integration runtime and scalability	STAligner	PRECAST	PASTE	-	-

This table ranks the top 5 most recommended tools for each analysis conducted. Clustering accuracy is evaluated using four metrics: Adjusted Rand Index (ARI), Normalized Mutual Information (NMI), Adjusted Mutual Information (AMI), and Homogeneity (HOM). Clustering continuity is assessed with three metrics: Average Silhouette Width (ASW), CHAOS, and Percentage of Abnormal Spots (PAS)

### Alignment vs. integration methods

While alignment and integration methods are capable of conducting multi-slice analysis, alignment methods such as PASTE, PASTE2, SPACEL, STalign, and GPSA typically produce spot-to-spot alignment matrices or transformed spot coordinates based on alignment. In contrast, integration methods using deep learning backbones often generate joint spot embeddings for subsequent integration analyses. Therefore, it was not surprising to see that SPACEL and PASTE exhibited higher accuracy in layer-wise alignment compared to all integration tools as the primary objective of alignment methods was the direct alignment of spots across slices, rather than relying on joint spot embeddings for integration analysis. Relying on the joint spot embeddings to align spots across slices, STAligner achieved the highest layer-wise alignment accuracy among all integration methods, followed by DeepST, while PRECAST performed the least accurately. These results highlighted, to some extent, the inherent qualities of their learned joint spot embeddings. Our additional visualization plots for alignment-misalignment-unalignment analysis and spot-to-spot mapping ratios revealed that integration tools such as STAligner, SPIRAL, DeepST, and PRECAST produced joint spot embeddings capable of capturing global features for coarse layer-wise alignment and integration. Nevertheless, they might not suffice for capturing the local geometry necessary for spot-to-spot alignment. Our simulation experiments provided further validation for this observation. Notably, among all tools, PASTE2 and SPACEL achieved better spot-to-spot alignment accuracy when slices partially overlapped. The performance of all integration methods was highly sensitive to perturbation on the expression profiles. Notably, PASTE2 exhibited the greatest robustness to these perturbations, followed by PASTE, SPACEL, and GPSA.

Most integration methods were initially designed to learn joint spot embeddings across multiple slices. UMAP plots, projecting embeddings into two components, can to some extent reflect integration performance. Among these methods, STAligner stood out with better UMAP visualization, demonstrating integration with batch correction. However, its performance degraded for the MHypo dataset compared to the DLPFC dataset. SPIRAL, on the other hand, suffered from a significant batch effect due to uneven mixing of joint embeddings across slices, leading to notable separation issues across slices for the MHypo dataset, consistent with its least favorable and super high spot-to-spot mapping ratio. PRECAST tended to lose substantial geometry information, resulting in a more noticeable segregation of spatial domains in the latent space compared to the other tools. Although joint spot embeddings learned by multi-slice analysis have the potential to provide us a way to better detect spatial domains or cell types compared to single-slice analysis, and certain tools demonstrated this potential improvement, it was difficult to conclude which tool had the overall best clustering performance in all pairs after integration. In summary, there is still a need for more robust integration tools. Integration methods could also align samples across different anatomic regions or development stages. We found STAligner outperformed other tools and had the scalability to process big datasets (over 50k spots).

As for the reconstruction of 3D architecture from multiple adjacent consecutive 2D slices, alignment tools such as PASTE and SPACEL outperformed integration tools like STAligner, SPIRAL, and GPSA. Specifically, when aligning a significant number of adjacent consecutive slices, SPACEL with manual annotation labels outperformed SPACEL without manual annotation labels and PASTE. This is because an erroneous alignment can trigger a cascade of errors in subsequent slices in SPACEL and PASTE. It is also worth noting that the 3D reconstruction by SPACEL is not deterministic and exhibits variance. Finally, in terms of runtime for alignment and integration, STAligner, PRECAST, and PASTE demonstrated good scalability for large datasets.

### Comparison with existing benchmarks

To date, two other benchmarking studies [57, 58] have been conducted for ST clustering methods. However, unlike the methods in these studies, which focused primarily on identifying spatial domains within a single slice, there is a growing recognition of the importance of integrative and comparative analyses across multiple ST slices. Integration analysis with adjacent slices also has the potential to enhance the detection of spatial domains compared to single-slice analysis. Therefore, in terms of the evaluation scope, our work provides a more comprehensive benchmarking study encompassing various types of methods, including clustering, alignment, and integration algorithms, evaluated on both real and simulated datasets. Our study includes the most extensive collection of clustering tools to date and also offers a pair-wise evaluation of clustering performance both before and after integration, with a focus on tools such as BASS, PRECAST, and DeepST. For alignment and integration analyses, we have designed several specific qualitative and quantitative metrics, including layer-wise and spot-to-spot alignment accuracy, visualization for alignment-misalignment-unalignment, and spot-to-spot mapping ratio. These metrics are designed to enhance our understanding of the joint embeddings generated by integration methods and to highlight the significant performance differences between alignment and integration methods.

While it is challenging to identify a single best tool, we have summarized results and offered a comprehensive recommendation based on a broad range of metrics and scenarios, enabling users to select the most suitable tools for their needs. Notably, there are common and important recommendations for clustering tools benchmarked in our work and others. For instance, BASS demonstrated the best clustering accuracy and generalizability across different datasets. While SpaceFlow and CCST did not achieve the highest overall clustering accuracy, they excelled in contiguity. Certain tools, like GraphST, exhibited technology-biased performance. While it performed well in 10x Visium datasets, its performance declined with STARmap and MERFISH datasets, which were not specialized data types for GraphST. STATAGE had the best runtime and scalability for big datasets. However, there are also some important recommendations for tools like ConGI, BANKSY, SpatialPCA, and ADEPT, which were never benchmarked in other work. ConGI is the most effective tool for tumor datasets, although its performance declines with non-tumor datasets. BANKSY, ADEPT, and SpatialPCA are top tools across most recommendation scenarios.

## Conclusions

As spatial transcriptomic data become more widely used in studying complex tissues, numerous methods for clustering, alignment, and integration are developed each year. In this benchmark study, we highlight several essential aspects to guide further method development. (1) Robust clustering methods: it is crucial to build robust clustering methods that excel in terms of both clustering accuracy and continuity and are capable of handling large-scale spatial omics datasets efficiently, thereby reducing analysis time and resources. (2) Avoid overfitting: minimize excessive parameter tuning on well-studied datasets to ensure that models generalize effectively across diverse datasets. (3) Joint embedding learning: developing methods to learn and utilize joint embedding for integration and spatial domain identification while capturing the data geometry for better alignment. (4) 3D visualization: creating tools for the 3D visualization of spatial omics data is necessary to better represent complex tissue architectures. (5) Incorporation of advanced spatial data types: many current methods primarily focus on transcriptomics data, often overlooking other advanced spatial data types like spatial proteomics and metabolomics, which could offer complementary insights. To address these limitations, future research should aim to incorporate spatial multi-omics data and design sophisticated computational methods, such as multi-model deep learning networks or multi-model statistical approaches for heterogeneous data integration and joint learning.

## Methods

### Clustering methods overview

#### BANKSY

BANKSY [21] utilizes a spatial feature augmentation strategy to cluster spatial omics data. It enhances each cell’s features with the average features of its neighboring cells and gradients of features across neighborhoods. By integrating neighborhood details into clustering, BANKSY can detect spatial domains that share similar microenvironments.

#### ADEPT

ADEPT [28] relies on a graph autoencoder backbone and performs an iterative clustering on imputed, differentially expressed genes-based matrices to minimize the variance of clustering results. The learned representations are suitable for subsequent clustering analyses.

#### GraphST

GraphST [4] enhances ST analysis in terms of spatial clustering, multisample integration, and cell-type deconvolution by combining graph neural networks with self-supervised contrastive learning. The learned spot representations are suitable for clustering analyses.

#### SpaceFlow

SpaceFlow [27] employs spatially regularized deep graph networks to combine gene expression similarities with spatial information. This process generates spatially-consistent low-dimensional embeddings that are suitable for subsequent clustering analyses.

#### conST

conST [25] is a versatile SRT data analysis framework employing contrastive learning techniques. conST integrates multi-modal ST data-gene expression, spatial information, and morphology (if applicable)-to learn low-dimensional embeddings. These embeddings are suitable for various downstream analyses.

#### ConGI

ConGI [26] detects spatial domains by integrating gene expression and histopathological images, adapting gene expression to image information via contrastive learning. The learned representations are valuable for various downstream analyses.

#### SpatialPCA

SpatialPCA [19], a spatially aware dimension reduction method for ST data, extracts a low-dimensional representation of gene expression. It enhances the probabilistic version of PCA with localization information, employing a kernel matrix to model spatial correlation across tissue locations. The resulting components are termed spatial principal components (PCs).

#### DR.SC

DR.SC [20] employs a two-layer hierarchical model that simultaneously performs dimension reduction via a probabilistic PCA model and enhances spatial clustering using an HMRF based on empirical Bayes. DR.SC is characterized by automatical determination of the optimal number of clusters.

#### STAGATE

STAGATE [3] leverages a graph attention auto-encoder architecture for spatial clustering by integrating spatial information and gene expression profiles to derive low-dimensional embeddings. The learned embeddings are suitable for subsequent clustering analyses.

#### CCST

CCST [24] utilizes an extended Deep Graph Informax (DGI) framework by incorporating a hybrid adjacent matrix for gene expression and spatial data. It encodes cell embeddings and then employs PCA for dimension reduction. k-means++ was applied for clustering to identify novel cell groups or subpopulations.

#### SEDR

SEDR [23] learns low-dimensional representations of gene expression data with spatial information. It uses deep autoencoder networks and variational graph encoders for spatial embeddings. SEDR is proficient in handling high-resolution ST data.

#### SpaGCN

SpaGCN [22] utilizes a graph convolutional network to unify gene expression, spatial location, and histology data to identify spatial domains with coherent expression and histology. Subsequently, SpaGCN conducts domain-guided differential expression analysis to detect genes exhibiting enriched expression within identified domains across various ST studies.

#### BayesSpace

BayesSpace [17], a fully Bayesian method, enhances resolution in ST data by integrating spatial neighborhood information for clustering analysis. It employs a t-distributed error model and Markov chain Monte Carlo (MCMC) for spot-level clustering, promoting neighboring cells to share clusters. It refines cell clustering by dividing spots into subspots with their neighbors.

### Alignment and integration methods overview

#### STalign

STalign [36] utilizes diffeomorphic metric mapping to align ST datasets, accommodating partially matched tissue sections and local non-linear distortions. It effectively aligns ST datasets within and across technologies, as well as to a 3D common coordinate framework.

#### GPSA

GPSA [37] employs a Bayesian model to align spatially-resolved samples to a common coordinate system (CCS) based on phenotypic readouts like gene expression. It involves a two-layer Gaussian process. The first layer maps the spatial locations of observed samples to the CCS, while the second layer maps from the CCS to the observed readouts.

#### SPIRAL

SPIRAL [42] performs the integration task and the alignment task through two consecutive modules: SPIRAL-integration, focusing on data integration using graph domain adaptation, and SPIRAL-alignment, centered around alignment using cluster-aware optimal transport coordination.

#### STAligner

STAligner [39] employs a graph attention auto-encoder neural network to extract spatially aware embeddings and constructs the spot triplets based on embeddings to guide different slices’ integration and alignment process.

#### PRECAST

PRECAST [41], an integration method, takes normalized gene expression matrices from multiple tissue slides as input. It factorizes each matrix into latent factors shared within cell/domain clusters, while performing spatial dimension reduction and clustering. It also aligns and estimates joint embeddings for biological effects between cell/domain types across the slides.

#### SPACEL

SPACEL [35] includes three modules: Spoint deconvolutes cell type composition per spot using a probabilistic multiple-layer perceptron in a single ST slice; Splane identifies coherent spatial domains across multiple slices via a graph convolutional network and adversarial learning; Scube constructs a 3D tissue architecture by transforming and stacking consecutive slices.

One important note for SPACEL in this benchmark work is that only the Scube module is utilized for alignment and 3D reconstruction for the MHypo and simulated datasets. This is achieved by incorporating manual annotation labels, as single-cell reference is not available for the initial Spoint module to perform deconvolution.

#### PASTE

PASTE [33] employs an fused Gromov-Wasserstein optimal transport formulation to compute pairwise alignments of slices, integrating both transcriptional similarity and physical distances between spots. Moreover, PASTE aggregates these pairwise alignments to create a stacked 3D alignment of a tissue.

#### PASTE2

PASTE2 [34] introduces a novel formulation of the partial fused Gromov-Wasserstein optimal transport problem to addresses partial alignment and 3D reconstruction of multi-slice ST datasets. It accommodates scenarios wit partial overlap between aligned slices and/or slice-specific cell types.

#### BASS

BASS [18] detects spatial domains and clusters cell types simultaneously using a hierarchical Bayesian model. BASS performs well in identifying rare cell types and spatial patterns, showing robustness in handling multiple dominant cell types within spatial domains.

#### DeepST

DeepST [40] uses neural networks, including a graph autoencoder and a denoising autoencoder, to jointly process the data and generate latent representations. Additionally, DeepST incorporates domain adversarial neural networks to integrate the ST data effectively.

### Quantitative analysis for clustering

#### Benchmark metrics

 

- Adjusted Rand Index (ARI) [59]: ARI is a measure of the similarity between two data clusterings. It is a correction of the Rand Index, which evaluates the concordance between pairs of data points, determining whether they are grouped together or separated in two different clusterings. The ARI value is calculated using Eqs. 1 and 2. *a* is the number of pairs of elements that are in the same cluster in both the ground true and predicted clusterings, *b* is the number of pairs of elements that are in different clusters in both the ground true and predicted clusterings, *c* is the number of pairs of elements that are in the same cluster in the true clustering but in different clusters in the predicted clustering, and *d* is the number of pairs of elements that are in different clusters in the true clustering but in the same cluster in the predicted clustering. *E*(*RI*) is the expected value of the Rand Index under the assumption of independence between the true and predicted clusterings. *max*(*RI*) is the maximum possible Rand Index. The ARI value ranges from − 1 to 1, where 1 indicates perfect agreement between the clusterings, 0 indicates random clustering and negative values indicate clustering that is worse than random.
- Normalized Mutual Information (NMI) [59]: NMI is another measure commonly used to evaluate the similarity between two clusterings. It normalizes the Mutual Information (MI) score, evaluating the agreement between ground truth and predicted clusterings while considering both intra-cluster homogeneity and inter-cluster completeness. It ranges from 0 to 1: 0 signifies no mutual information (random clustering), while 1 indicates perfect agreement. The NMI value is calculated using Eqs. 3 and 4. *H*(*U*) and *H*(*V*) represent the entropy of the clustering *U* and *V*, respectively, while *MI*(*U*, *V*) denotes the MI between *U* and *V*.
- Adjusted Mutual Information (AMI) [59]: AMI is a measure commonly used to evaluate the similarity between two clusterings as NMI. It adjusts for chance agreement by considering the expected mutual information under random clustering. The AMI value ranges from − 1 to 1, where 1 signifies a perfect agreement between the clusterings, 0 indicates agreement expected purely by chance, and negative values indicate worse than chance agreement. To calculate AMI, Eqs. 5 and 4 are used.
- Homogeneity (HOM) [59]: HOM is a metric commonly used in clustering analysis to evaluate the quality of clusters produced by a clustering algorithm. Homogeneity score measures the purity of clusters (Eq. 6), indicating whether each cluster contains predominantly data points from a single group or if it contains a mixture of different groups. A high homogeneity score suggests that the clustering algorithm has successfully identified distinct and homogeneous clusters, while a low score indicates that the clusters are more heterogeneous and less well-defined.
- Average Silhouette Width (ASW) [55]: The ASW score is utilized to evaluate the spatial coherence of predicted domains concerning physical space in the ST field. ASW values range from − 1 to 1 (rescaled from 0 to 1), with higher values indicating better performance. To compute ASW, the silhouette width (SW) must first be defined, followed by averaging SWs across all cells. SW for a cell, described in Eq. 7, is calculated based on the mean distance to all other cells in the same spatial domain *a* and the mean distance to all other cells in the next nearest cluster *b*.
- CHAOS [19]: The CHAOS score is used to measure the spatial continuity of the detected spatial domains in the ST field, as described in Eqs. 8 and 9. CHAO values range from 0 to N/A. Lower CHAOS value indicates higher spatial continuity and better performance.
- Percentage of Abnormal Spots (PAS) [19]: The PAS score assesses the spatial homogeneity of spatial domain identification algorithms in the ST field. It is computed by determining the proportion of spots with a cluster label different from at least six out of their neighboring ten spots. A low PAS score suggests homogeneity of spots within spatial clusters. PAS values range from 0 to 1.
- Spatial Coherence Score (SCS): A spatial coherence score of the cluster labels is computed based on O’Neill’s spatial entropy. A high spatial (more negative from the entropy) coherence score indicates that the cluster labels of adjacent spots are frequently identical, while a low spatial coherence score (less negative for the entropy) suggests that cluster labels of adjacent spots are more chaotic and less coherent. This score serves as an indicator of data quality. Specifically, let $G = (V, E)$ be a graph where $V$ is the set of spots, and edges $(i, j) \in E$ connect every pair $(i, j)$ of adjacent spots. Let $K = \{1, 2, \ldots , k\}$ be a set of $k$ cluster labels, and let $L = [l(i)]$ be a set of labelings of spots, where $l(i) \in K$ is the cluster label of spot $i$. The spatial entropy $H(G, L)$ is defined in Eq. 10, where $P(\{a, b\} | E) = \frac{n_{a, b}}{|E|}$, and $n_{a, b}$ is the number of edges $(i, j) \in E$ such that $l(i) = a$ and $l(j) = b$. The spatial coherence score is defined as a normalized form of spatial entropy, using the value of the Z score of spatial entropy over random permutations of the labels of spots in a slice [33].
- Runtime: We collected the average runtimes from 20 iterations for each clustering method across all benchmarking datasets to assess their scalability.
  1 $$\begin{aligned} ARI = \frac{\text {RI} - \text {E(RI)}}{max(\text {RI}) - \text {E(RI)}} \end{aligned}$$
  2 $$\begin{aligned} RI = \frac{a+b}{a+b+c+d} \end{aligned}$$
  3 $$\begin{aligned} NMI(U, V) = \frac{MI(U, V)}{\sqrt{H(U) \times H(V)}} \end{aligned}$$
  4 $$\begin{aligned} MI(U,V)=\sum _{i=1}^{|U|} \sum _{j=1}^{|V|} \frac{|U_i\cap V_j|}{N} \log \frac{N|U_i \cap V_j|}{|U_i||V_j|} \end{aligned}$$
  5 $$\begin{aligned} AMI(U, V) = \frac{MI(U, V) - E(MI(U, V))}{avg(H(U), H(V)) - E(MI(U, V))} \end{aligned}$$
  6 $$\begin{aligned} HOM(U,V) = \frac{MI(U, V)}{H(U)} \end{aligned}$$
  7 $$\begin{aligned} SW = \frac{b-a}{max(a, b)} \end{aligned}$$
  8 $$\begin{aligned} CHAOS = \frac{\sum ^{K}_{k=1}\sum ^{n_k}_{i,j}w_{kij}}{N} \end{aligned}$$
  9 $$\begin{aligned} w_{kij} = \left\{ \begin{array}{ll} d_{ij} & \text {if connected in the}\ k^{th}\ \text {1NN graph} \\ 0 & \text {otherwise} \end{array}\right. \end{aligned}$$
  10 $$\begin{aligned} H(G, L) = -\sum\nolimits _{a, b \in K} P(\{a, b\} | E) \log (P(\{a, b\} | E)) \end{aligned}$$

#### Domain identification performance across 33 ST slices

Given that spatial domain or cell type identification is the primary objective of clustering methods, we aim to conduct a thorough performance comparison using ARI when manual annotation serving as ground truth is available. Some deep learning-based methods and all statistical methods fix the seed to produce deterministic output, some deep learning-based methods do not fix the seed in the practice. To address the variances in performance, we computed the average ARI from 20 runs on each dataset and displayed these results using box plots and a heatmap plot to enhance comparison and visualization. Additionally, since there are 33 ST slices across eight different datasets, it is challenging to rank the overall performance solely based on the average ARI heatmap plot. Therefore, we also provided another heatmap for the overall ranking. This ranking heatmap was generated by normalizing all results within the same slice by dividing them by the maximum ARI value (representing the best performance) among all methods, thereby standardizing all ARI values to 1. With 33 data slices in total, for each method, the best ranking for the sum result is 33, while the best ranking for the average result is 1. To ensure fairness, the rank scores were averaged exclusively over feasible ST data, excluding instances with NaN values. We performed the same analysis based on the NMI, AMI, and HOM metrics.

#### Overall robustness across seven ST datasets

To assess the robustness of methods on each dataset, the clustering results across different ST slices within the same dataset were averaged. A robust method is expected to demonstrate the highest overall ARI, NMI, AMI, or HOM value across all datasets, even if it may encounter challenges in predicting a few individual slices.

#### Data complexity effect on method performance

Data complexity is recognized to have an impact on method performance. Although different methods are often fine-tuned on different datasets to demonstrate superiority in specific contexts, our objective is to identify a general trend wherein methods exhibit diminished performance as data complexity increases. In this context, the Average Silhouette Width (ASW), CHAOS, Percentage of Abnormal Spots (PAS), and Spatial Coherence Score (SCS) are introduced as metrics for quantifying data complexity. The underlying assumption is that data with more coherent regions, indicated by a higher ASW/SCS (or lower CHAOS/PAS), are easier for domain identification.

### Qualitative analysis for clustering

#### Clustering evaluation by visualization

For MHPC data without region-based annotation, the evaluation is constrained to comparing the clustering results with the cell type annotation through visualization, supplemented by reference to the Mouse Allen Brain atlas.

### Quantitative analysis for alignment and integration

#### Benchmark metrics

 

- Adjusted Rand Index: As illustrated in the clustering metrics section.
- Layer-wise alignment accuracy: This metric relies on an important hypothesis that aligned spots from adjacent consecutive slices within a dataset are more likely to pertain to the same spatial domain or cell type. Joint spot embeddings learned from each method are utilized to align (anchor) spots from the first slice to (aligned) spots on the second slice for each slice pair. This alignment accuracy is defined as the ratio of the number of anchor spots to the total number of spots within the first slice when anchor spots and aligned spots belong to the same spatial domain or cell type. Euclidean distance is employed to define the closeness of spots to be aligned. A good integration tool is expected to demonstrate high accuracy for anchor and aligned spots belonging to the same spatial domain or cell type. For DLPFC data which has a unique layered structure, this metric is also meticulously designed to demonstrate whether anchor and aligned spots belong to the same layer (layer shift = 0) or they belong to different layers (layer shift = 1 to 6).
- Spot-to-spot matching ratio: This metric further evaluates whether joint embeddings’ quality captures the data geometry. The ratio is defined as the ratio of the total number of anchor spots from the first slice to the number of aligned spots from the second slice. For two adjacent consecutive slices, a nearly 1:1 ratio is expected for an optimal tool.
- Spot-to-spot alignment accuracy: This metric is used to evaluate joint embeddings for simulated datasets since the ground truth for spot-to-spot alignment relationship is available. This spot-wise alignment accuracy is defined as the percentage of anchor spots from the first slice that match correctly to aligned spots on the second slice.

#### Comparison of clustering performance before and after integration

A good practice that connects integration and clustering tasks is multi-slice joint clustering. To determine if incorporating information from adjacent consecutive slices enhances domain or cell type identification, we used batch-corrected joint embeddings to evaluate clustering results on each single slice based on ARI values. We plotted ARI for clustering results before and after the integration. However, some integration methods do not support single-slice clustering. We thus only plotted ARI after the integration of these methods.

#### Simulated data for alignment and integration

Given the scarcity of benchmark datasets available for integration tasks to evaluate spot-to-spot alignment accuracy, we modified the simulation method proposed in PASTE [33] and generated 11 simulated 10x Visium datasets for this evaluation. We first used one DLPFC slice (151673) as the reference and simulated additional slices with different overlapping ratios (20%, 40%, 60%, 80%, and 100%) in comparison to the reference slice. In this simulation scenario, the pseudocount perturbation was fixed at 1.0 for all simulated slices. Next, we simulated additional slices with different pseudocounts (0–3.0 with a step size of 0.5) to represent perturbation on gene expression while keeping the overlapping ratio fixed at 100%. Specifically, by taking the DLPFC 151673 slice as the reference, we altered the spatial coordinates in the new slice by rotating this reference slice, perturbed the gene expression by adding pseudocounts, and adjusted the number of spots by removing some spots that did not align with the grid coordinates following the rotation. To keep fidelity with the real 10x Visium data, the spots within the tissue in our simulation are arranged in a hexagonal grid rather than in a rectangular grid pattern. Additionally, we utilized the minimal distance between adjacent spots on the DLPFC 151673 slice as the distance between any two adjacent simulated spots on the grid, rather than arbitrarily setting it to 1.

More detailed procedures to generate simulated datasets are described as follows.

- Create a hexagonal grid *G*. Let $g_{.i}$ and $z_{.k}$ denote the 2D coordinates of spot *i* on grid *G* and spot *k* on the reference slice DLPFC 151673, respectively. $d_{ij}=||g_{.i}-g_{.j}||=\min _{kl}||z_{.k}-z_{.l}||$ for any two adjacent simulated spots *i* and *j* on grid that $i, j \in G$.
- Let *R* be a rotation matrix with an angle $\theta$. After spot *k* is rotated with an angle $\theta$, the rotated coordinates of spot *k*, $r_{.k}=Rz_{.k}$, is used to mapped the spot *k* to the closest grid spot $\hat{i}$ by $\hat{i}=\arg \min _{i}||g_{.i}-r_{.k}||$. Then, the simulated coordinates of tissue spot *k*, $z'_{k}$ is given by $z'_{k}=g_{.\hat{i}}$. Spot *k* is dropped if the grid spot $g_{.\hat{i}}$ was already used by a previous tissue spot.
- Let $X=[x_{ij}] \in \mathbb {N}^{m\times n}$ represent the *m* genes by *n* spots expression profile matrix of DLPFC slice 151673, where $x_{ij}$ is the read count of gene *i* in tissue spot *j*. We can calculate the mean of the total transcript count of the tissue spots, $\mu = \frac{1}{n}\sum _{ij}x_{ij}$, and the variance of the total read count, $\sigma ^2=\frac{1}{n}\sum _{j}(\mu -\sum _{i}x_{ij})^2$. Total read counts of spot *j*, $k_j$, are generated according to $k_j \sim$NegativeBinomial(*r*, *p*). Here, $r = \frac{\mu ^2}{\sigma ^2-\mu }$ and $p = \frac{\mu }{\sigma ^2}$ such that $E(k_j)=\mu$ and var$(k_j)=\sigma ^2$.
- Generate simulated gene *i* read count for spot *j* according to $x'_{ij} \sim$Multinomial$(k_j, \frac{x_{ij}+\delta }{\sum _ix_{ij+\delta m}})$, where $\delta \in \{0, 0.5, \dots , 3\}$ is a pseudocount.

### Qualitative analysis for alignment and integration

#### Visualization of aligned, misaligned and unaligned spots from pairwise alignment

To assess the joint spot embeddings by integration tools and the alignment matrices by alignment tools, we quantified the alignment accuracy based on aligned, misaligned, and unaligned spots across two consecutive slices. For integration tools such as STAligner, PRECAST, DeepST, and SPIRAL, we aligned the spot (referred to as the “anchor” spot) on the first slice with the spot (referred to as the “aligned” spot) on the second slice based on their joint latent embeddings using Euclidean distance. If the aligned spot belonged to the same spatial domain or cell type as the anchor spot according to ground truth labels, we classified both spots as “aligned” spots (denoted as “orange” color in Fig. 8a, b). If the aligned spot did not belong to the same spatial domain or cell type as the anchor spot, we classified both spots as “misaligned” spots (denoted as “blue” color in Fig. 8a, b). In the last scenario, if spots on the second slice were not used to match any spot on the first slice, these spots on the second slice were classified as “unaligned” spots (denoted as “green” color in Fig. 8a, b). For alignment tools like PASTE, PASTE2, SPACEL, STalign, and GPSA, we directly used their alignment matrices or refined coordinates to perform this analysis.

#### Reconstruction of three-dimensional (3D) architecture of the tissue

Among all alignment and integration methods, Tools such as PASTE, PASTE2, SPACEL, SPIRAL, STalign, and GPSA have an output for a transformed coordinate system for all slices. Tools like STAligner use an embedded algorithm like ICP to align different slices based on an anchor cluster. Consequently, they can combine pairwise alignments from multiple adjacent consecutive slices into a stacked 3D alignment of a tissue. These three tools were benchmarked in three datasets by comparing their 3D architecture of the tissue.

One important note for SPACEL is that the 3D architecture for the MB dataset was reconstructed in two scenarios: (1) using the Scube module with manual annotation labels and (2) using both the Splane and Scube modules, incorporating the cell-type decomposition results provided by the authors.

#### Visualization of UMAP plot for joint embeddings

Most integration methods primarily concentrate on embedding the spots within a high-dimensional latent space, which often proves challenging to interpret intuitively. To enhance comprehension of the distribution in the latent space, we performed dimension reduction for spot embeddings to two dimensions using UMAP. A quality UMAP plot of latent embeddings should exhibit structures resembling those of the real data while also demonstrating spatial domain or cell types in a separable manner.

#### Visualization of clustering results after integration

For the MB2SA&P dataset, we compared the identified domains after integration with the Allen Brain atlas through visualization. Furthermore, we examined the consistency of regions across the fissure between the anterior and posterior sections. Higher similarity to the atlas, along with the region coherence, serve as indicators of superior integration performance.

For the mouse Embryo data, we compared the clustering result after integrating two slices for developmental stages E11.5 and E12.5 with the manual annotation defined by different organs and tissues.

### Computation platform

We conducted all benchmarking experiments on our computer server equipped with one Intel Xeon W-2195 CPUs, running at 2.3 GHz, featuring a total of 25 MB L3 cache, and comprising 36 CPU cores. The cluster also boasted 256 GB of DDR4 memory operating at 2666 MHz.

For the GPU configurations, we utilized the same computer with four Quadro RTX A6000 cards, each having 48 GB of memory and a total of 4608 CUDA cores.

### Supplementary information

Additional file 1: Ground truth annotation and parameter settings for benchmark tools. Table S1 provides a comprehensive overview of the ground truth for each dataset, detailing the specific ground truth labels and the information utilized in deriving them. Table S2 outlines the parameter settings and descriptions for each tool benchmarked.

Additional file 2: Supplementary results and Figures S1-S20.

Additional file 3. Review history.

## Data Availability

All code, tutorials, and related data files are freely available on GitHub [60] https://github.com/maiziezhoulab/BenchmarkST and on Zenodo [61] with DOI: https://doi.org/10.5281/zenodo.13128213 under MIT licenses. All data and the corresponding annotation can be downloaded from https://benchmarkst-reproducibility.readthedocs.io/en/latest/Data%20availability.html and are described in Table 1 with their sources. Dataset 1 consists of 12 human DLPFC sections, available at http://research.libd.org/spatialLIBD/ with manual annotation [43]. Dataset 2 [44] includes a single slice of human breast cancer, which is open-sourced from 10x genomics at https://www.10xgenomics.com/ with annotation [23]. Dataset 3 [45] includes two slices of anterior and posterior mouse brain available at https://www.10xgenomics.com/ with annotation [26]. Dataset 4 contains HER2-positive tumors from eight individuals at https://github.com/almaan/her2st with annotation [46]. Dataset 5, including anatomical regions of the mouse hippocampus, is acquired through the Broad Institute, available at https://singlecell.broadinstitute.org/ with annotation [47]. Dataset 6 is the Embryo dataset sequenced by Stereo-seq from the MOSTA project at https://db.cngb.org/stomics/mosta/resource/ with annotation [48]. Dataset 7 contains one slice from the mouse visual cortex and is available at https://www.starmapresources.org/data [9]. Dataset 8 contains three slices of the mouse prefrontal cortex and is available at https://www.starmapresources.org/data [9]. Dataset 9 [49] includes five slices from the mouse hypothalamus available at https://datadryad.org/stash/dataset/doi:10.5061/dryad.8t8s248 with annotation [18]. Dataset 10 [50] contains 33 consecutive mouse cerebral cortex tissue slices with similar shapes at https://zenodo.org/records/8167488 with annotation [35]. The simulation data is deposited in Zenodo https://zenodo.org/records/10800745 [62].
