# Supplementary material for: Benchmarking clustering, alignment, and integration methods for spatial transcriptomics
Source: Genome Biol. 2024 Aug 9;25:212. doi: 10.1186/s13059-024-03361-0 (PMC11312151; doi:10.1186/s13059-024-03361-0)
Supplement: Supplementary file 2 — Additional file 2: Supplementary results and Figures S1-S20. [file 13059_2024_3361_MOESM2_ESM.pdf]

Benchmarking clustering, alignment and integration methods for spatial transcriptomics

Hu et al.

Contents

1 Supplementary Results **3**

1.1 **The impact of GLM-PCA versus PCA on clustering performance** . . . 3

2 Supplementary Figures **4**

S1 ARI Bar plots for preprocessing or postprocessing with PCA versus GLM-PCA . . . 4

S2 The ground truth visualization plots and ARI box plots for the DLPFC dataset. . . . 5

S3 The ground truth visualization plots and ARI box plots for the HER2BT, MHypo, and MPFC datasets. . . . 6

S4 The plots of ARI versus loss value, ARI versus seed, and loss value versus seed by CCST. . . . 7

S5 The plots of ARI versus loss value, ARI versus seed, and loss value versus seed by ADEPT. . . . 8

S6 The plots of ARI versus loss value, ARI versus seed, and loss value versus seed by STAGATE. . . . 9

S7 The plots of ARI versus objective function value, ARI versus seed, and objective function value versus seed by BayesSpace. . . 10

S8 The plots of ARI versus seed by BASS. . . . 10

S9 Visualization plots for alignment-misalignment-unalignment for the DLPFC dataset. . . . 11

S10 Visualization plots for alignment-misalignment-unalignment for the DLPFC dataset. . . . 12

S11 Visualization plots for alignment-misalignment-unalignment for the DLPFC dataset. . . . 13

S12 Visualization plots for alignment-misalignment-unalignment for the DLPFC dataset. . . . 14

S13 Visualization plots for alignment-misalignment-unalignment for the MHypo dataset. . . . 15

S14 Visualization plots for alignment-misalignment-unalignment for the MHypo dataset. . . . 16

S15 UMAP plots of low dimensional joint embedding distribution for batch correction for the DLPFC dataset. 17
S16 UMAP plots of low dimensional joint embedding distribution for batch correction for the DLPFC dataset. 18
S17 UMAP plots of low dimensional joint embedding distribution for batch correction for the MHypo dataset. 19
S18 UMAP plots of low dimensional joint embedding distribution for batch correction. 20
S19 ARI Heatmap after integration. 21
S20 Reconstruction of 3D architecture of DLPFC by GPSA. 22

References **23**

1 Supplementary Results

1.1 The impact of GLM-PCA versus PCA on clustering performance

GLM-PCA is considered superior to conventional PCA for enhancing the low-dimensional representation of single-cell RNA sequencing (scRNA-Seq) data [1]. This is attributed to the fact that scRNA-Seq datasets with Unique Molecular Identifiers (UMIs) adhere to multinomial sampling without zero inflation. Additionally, standard normalization procedures, such as the logarithm of counts per million and feature selection based on highly variable genes (HVGs), introduce false variability in dimension reduction. To determine whether this phenomenon is also observed in spatial transcriptomics (ST) data, we selected four representative methods - SpaGCN, CCST, BASS, and BayesSpace - that utilize PCA in their preprocessing or postprocessing steps. In the preprocessing steps for BASS and BayesSpace, we substituted the standard normalization (logarithm of counts per million, spatially/highly variable genes selection, and PCA) with GLM-PCA. In the preprocessing steps for SpaGCN, we replaced the standard normalization (logarithm of counts per million and PCA) with GLM-PCA. For CCST, we substituted principal components (PCs) with GLM-PCs for learned latent embeddings in the postprocessing steps. We then analyzed whether this substitution enhances clustering performance in the DLPFC dataset. Our results, as depicted in Figure S1, indicate that SpaGCN, CCST, and BASS did not exhibit a significant performance difference when using GLM-PCA compared to PCA. In contrast, the performance of BayesSpace improved in 10 out of 12 slices for the DLPFC dataset. While it is challenging to conclusively state that GLM-PCA is superior to PCA in the ST field, it has the potential to enhance the performance of certain tools compared to conventional PCA.

2 Supplementary Figures

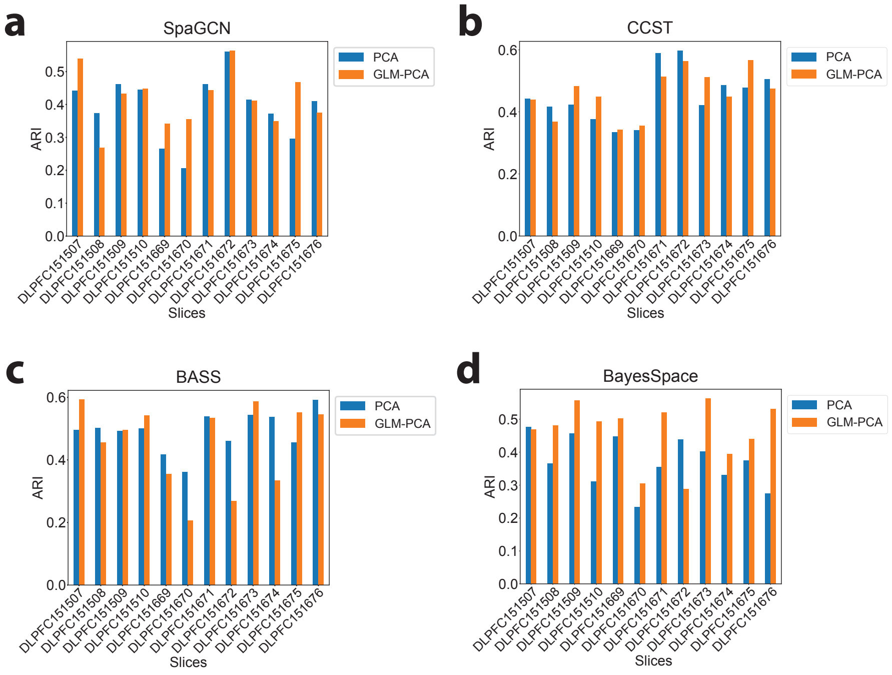

Figure S1: **ARI Bar plots for preprocessing or postprocessing with PCA versus GLM-PCA.** **a** ARI bar plots for SpaGCN using PCA versus GLM-PCA during the preprocessing step. **b** ARI bar plots for CCST using PCA versus GLM-PCA during the postprocessing step. **c** ARI bar plots for BASS using PCA versus GLM-PCA during the preprocessing step. **d** ARI bar plots for BayesSpace using PCA versus GLM-PCA during the preprocessing step.

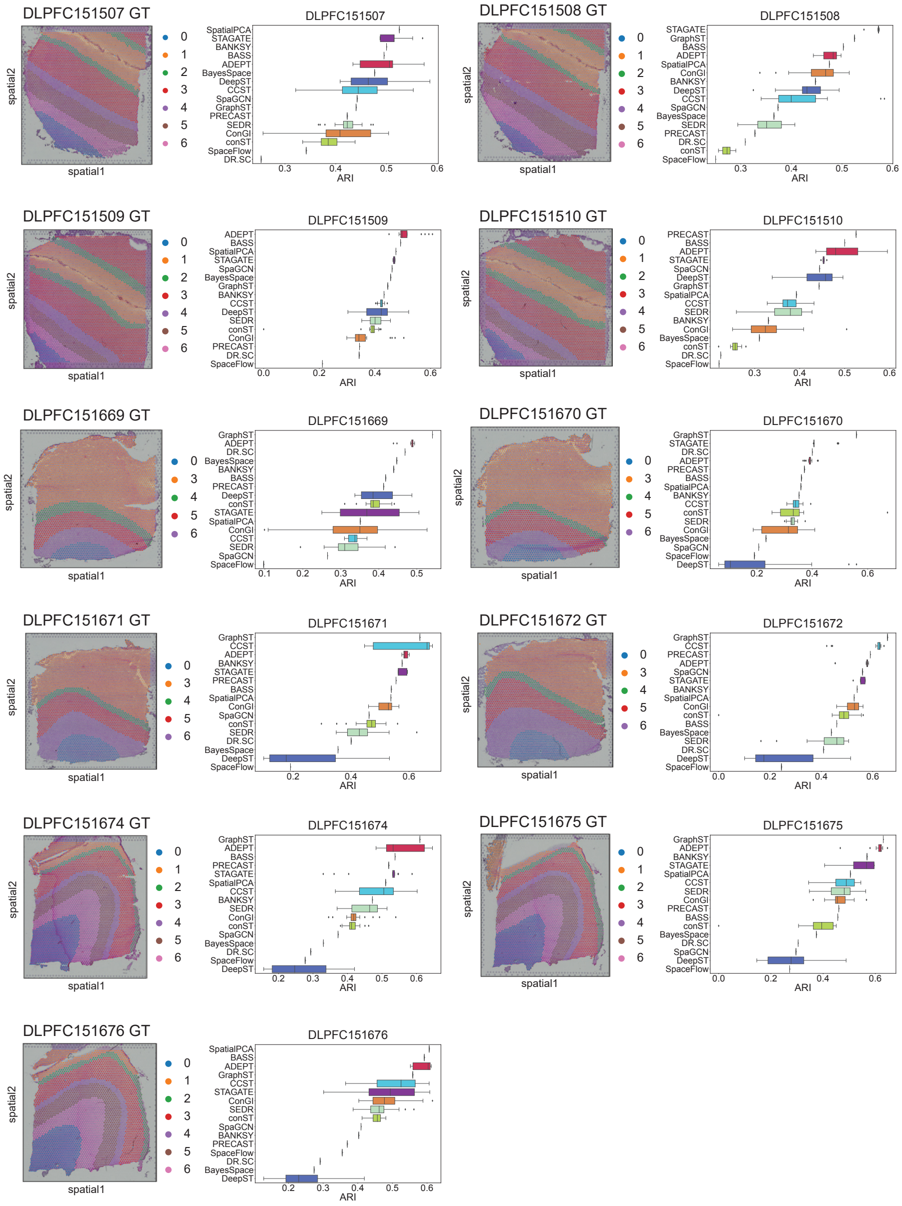

Figure S2: **The ground truth visualization plots and ARI box plots.** The ground truth visualization plots and box plots depicting ARI values of all tools on 11 DLPFC slices.

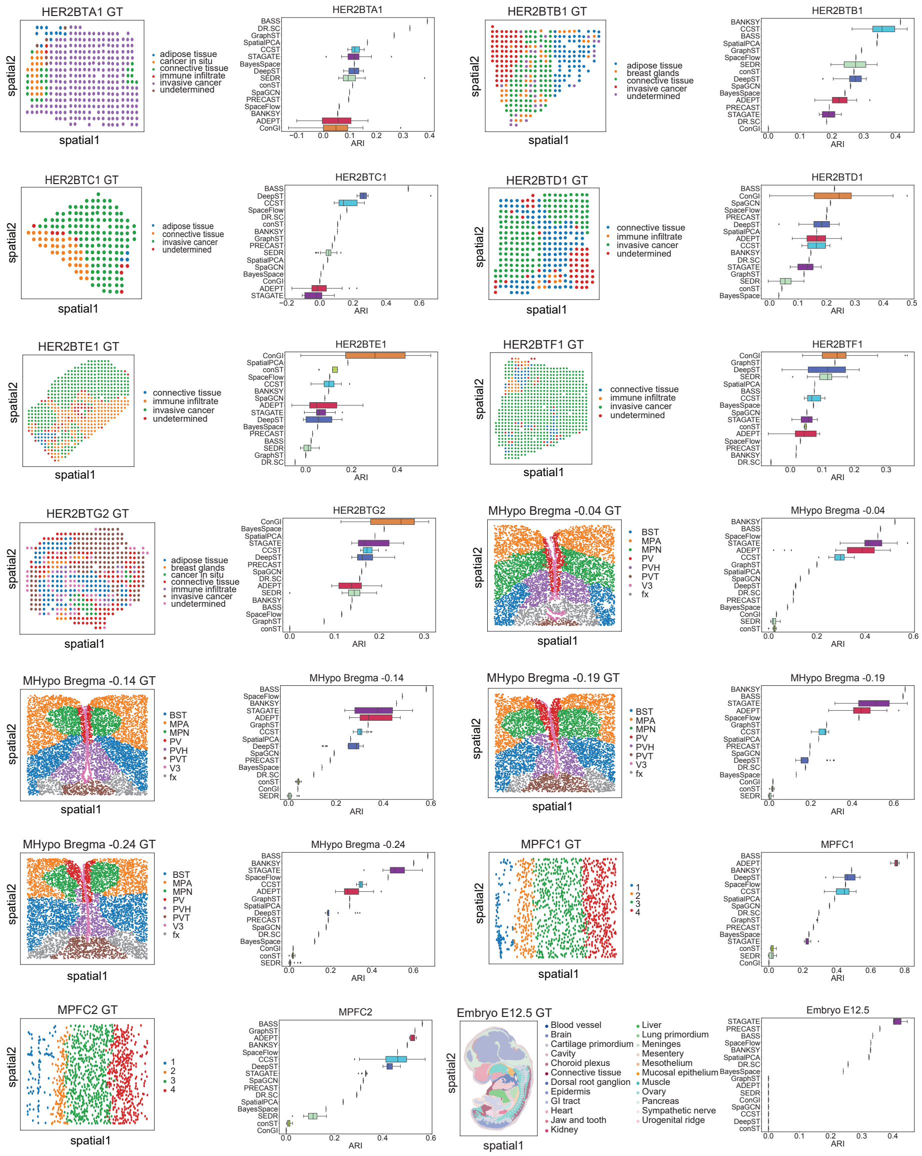

Figure S3: **The ground truth visualization plots and ARI box plots.** The ground truth visualization plots and box plots depicting ARI values of all tools on seven HER2BT slices, four MHypo slices, two MPFC slices, and one Embryo slice.

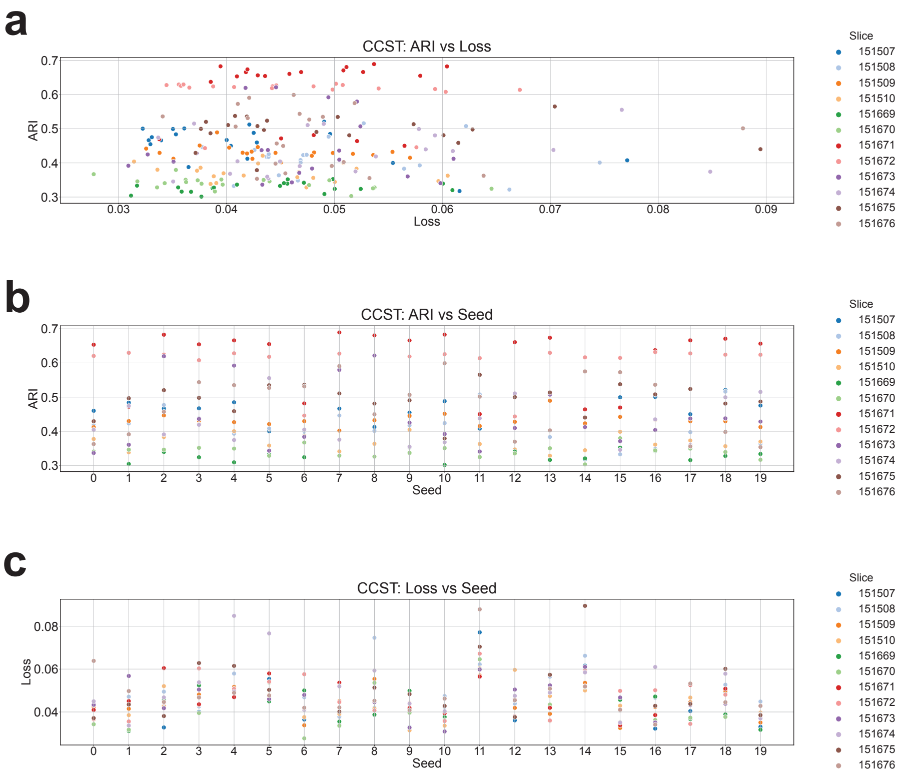

Figure S4: **The plots of ARI versus loss value, ARI versus seed, and loss value versus seed by CCST. a** ARI versus loss value. **b** ARI versus seed. **c** loss value versus seed.

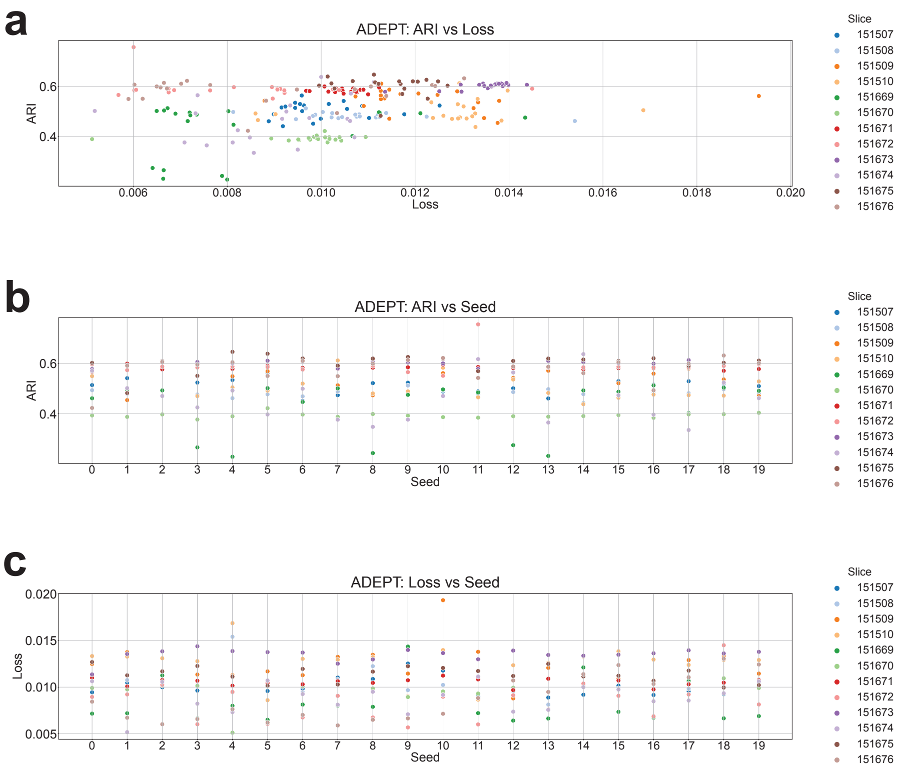

Figure S5: **The plots of ARI versus loss value, ARI versus seed, and loss value versus seed by ADEPT. a** ARI versus loss value. **b** ARI versus seed. **c** loss value versus seed.

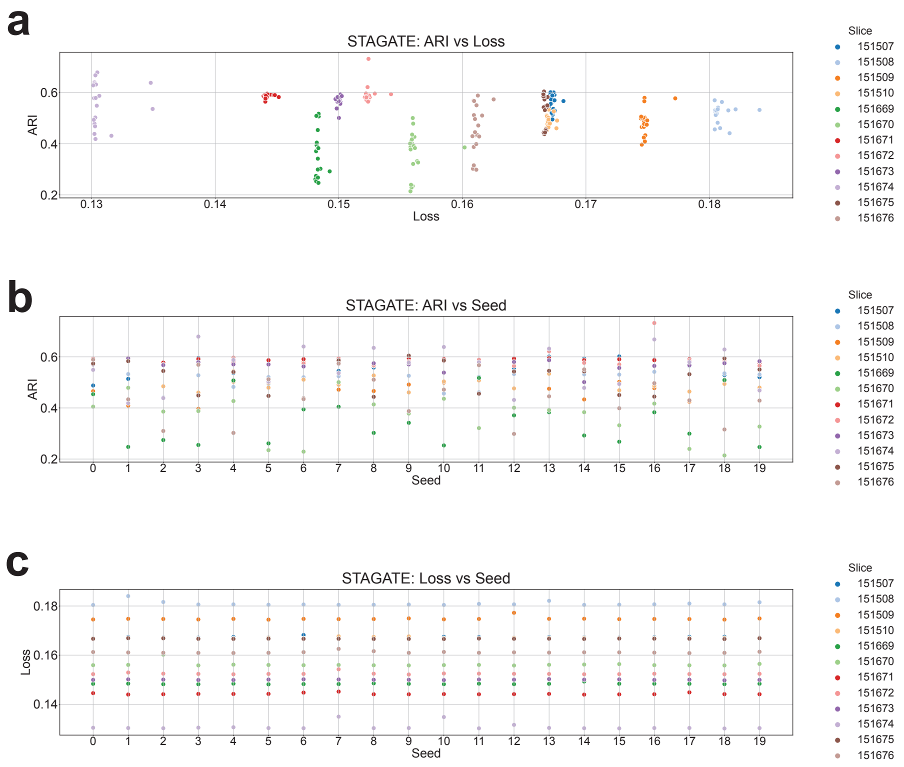

Figure S6: **The plots of ARI versus loss value, ARI versus seed, and loss value versus seed by STAGATE. a** ARI versus loss value. **b** ARI versus seed. **c** loss value versus seed.

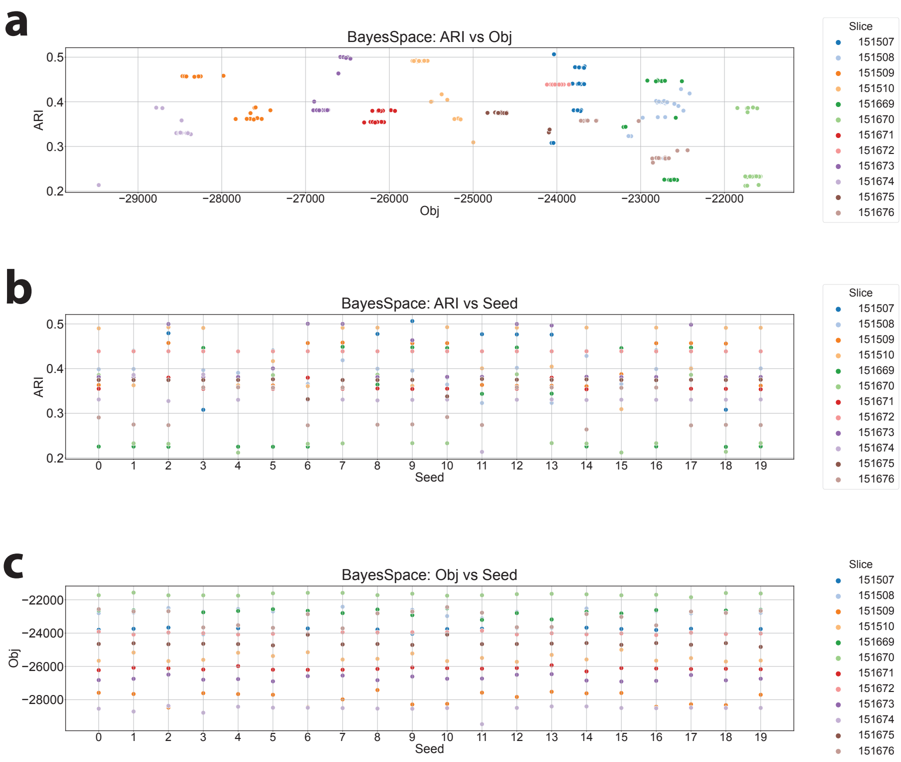

Figure S7: **The plots of ARI versus objective function value, ARI versus seed, and objective function value versus seed by BayesSpace. a** ARI versus objective function value. **b** ARI versus seed. **c** objective function value versus seed. BayesSpace has an objective function that we used, "NumericVector plogLik," to output its value.

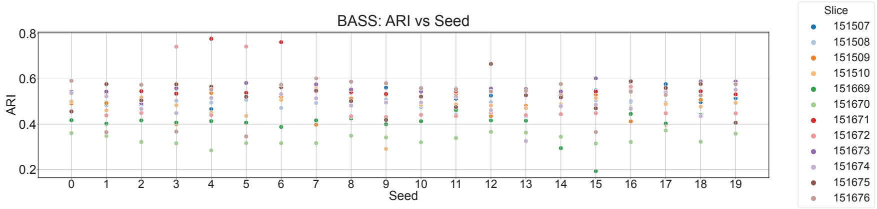

Figure S8: **The plots of ARI versus seed by BASS.** BASS does not include an objective function for performance optimization.

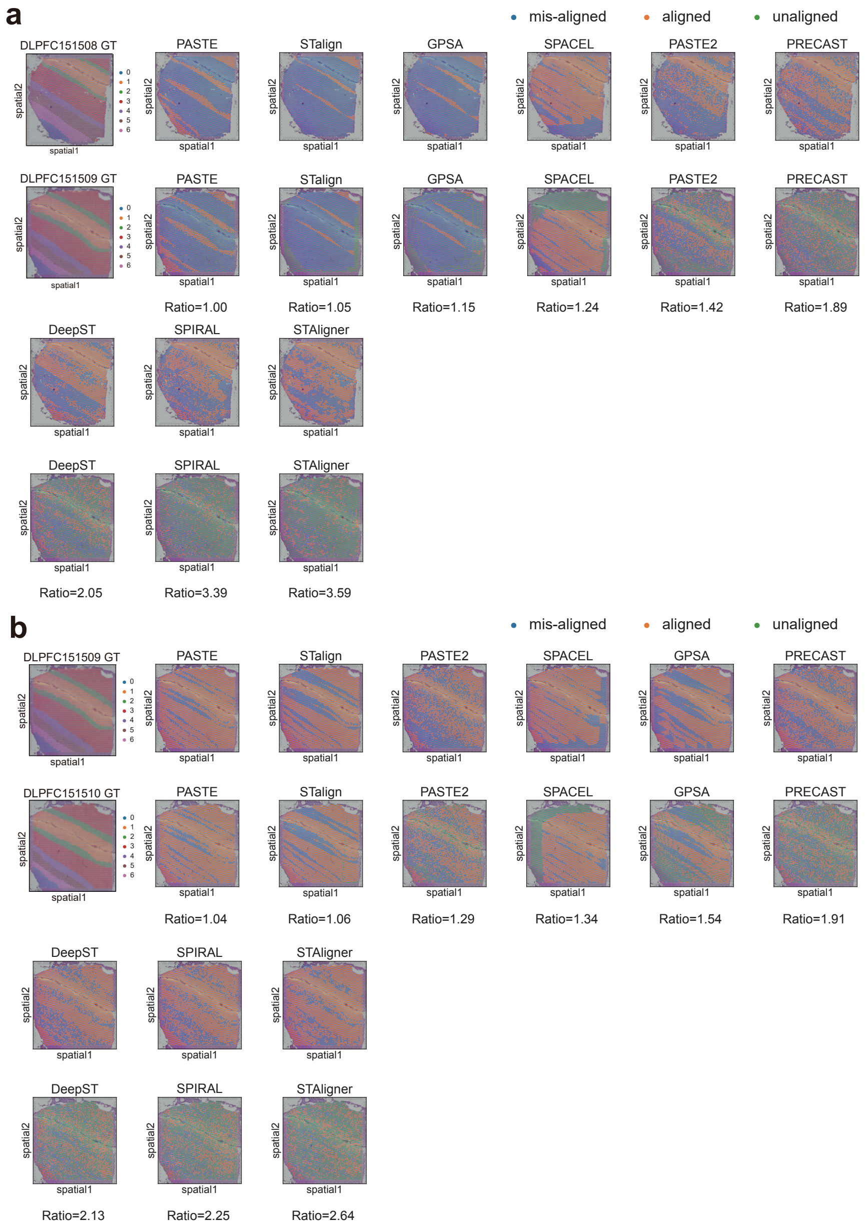

Figure S9: **Visualization plots for alignment-misalignment-unalignment for the DLPFC dataset. a-b** These visualization plots show aligned spots, misaligned spots, and unaligned spots when aligning the anchor spot from the first (top) slice to the aligned spots on the second (bottom) slice on DLPFC 151508-151509 (a) and 151509-151510 (b). Values below each plot represent the spot-to-spot matching ratio.

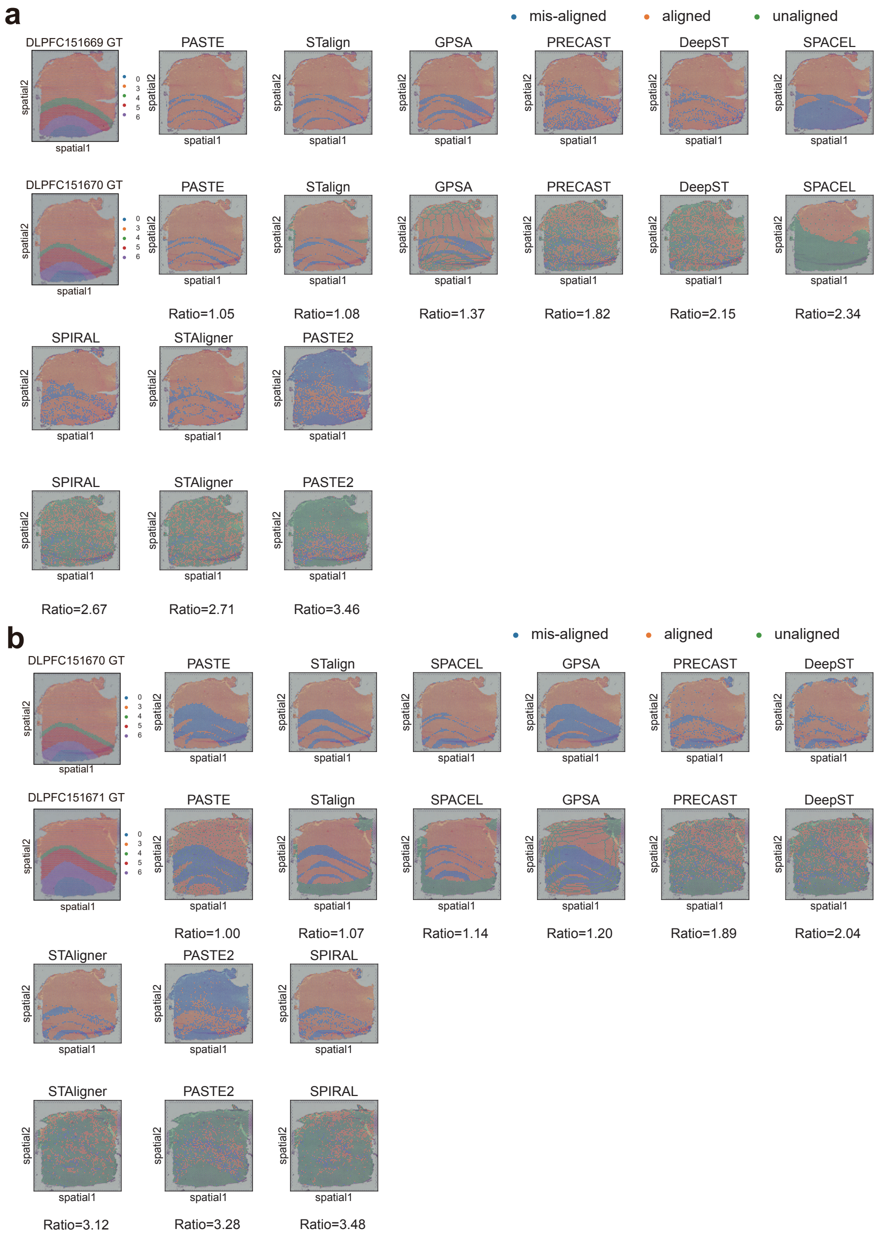

Figure S10: **Visualization plots for alignment-misalignment-unalignment for the DLPFC dataset. a-b** These visualization plots show aligned spots, misaligned spots, and unaligned spots when aligning the anchor spot from the first (top) slice to the aligned spots on the second (bottom) slice on DLPFC 151669-151670 (a) and 151670-151671 (b) pairs. Values below each plot represent the spot-to-spot matching ratio.

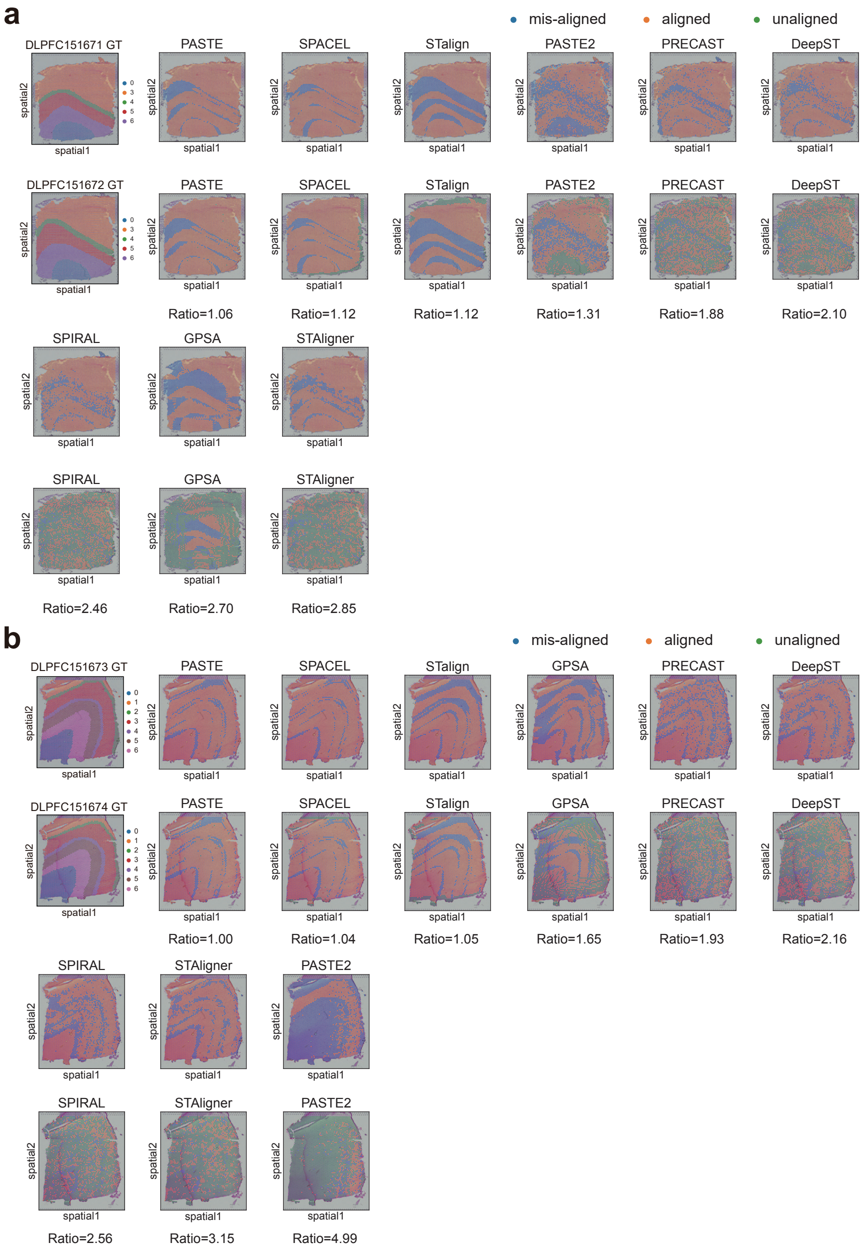

Figure S11: **Visualization plots for alignment-misalignment-unalignment for the DLPFC dataset.** **a-b** These visualization plots show aligned spots, misaligned spots, and unaligned spots when aligning the anchor spot from the first (top) slice to the aligned spots on the second (bottom) slice on DLPFC 151671-151672 (a) and 151673-151674 (b) pairs. Values below each plot represent the spot-to-spot matching ratio.

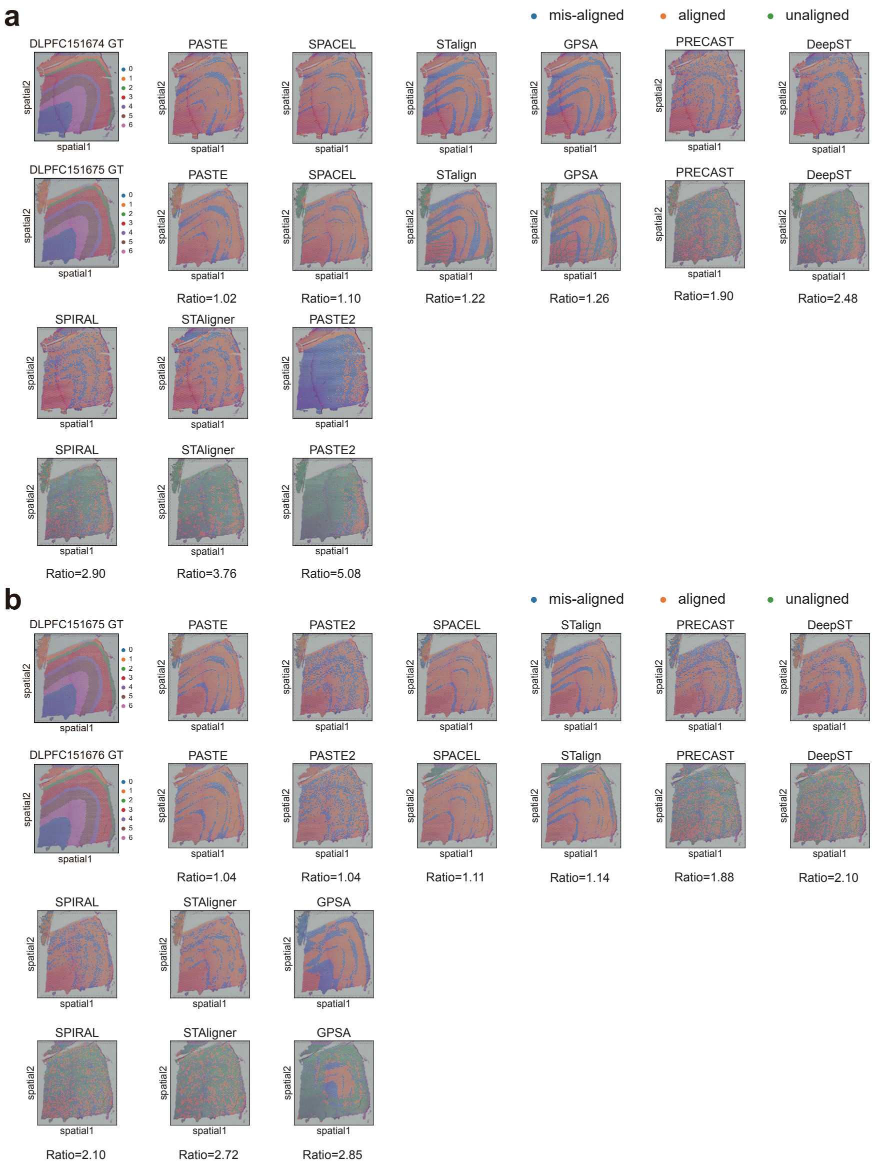

Figure S12: **Visualization plots for alignment-misalignment-unalignment for the DLPFC dataset. a-b** These visualization plots show aligned spots, misaligned spots, and unaligned spots when aligning the anchor spot from the first (top) slice to the aligned spots on the second (bottom) slice on DLPFC 151674-151675 (a) and 151675-151676 (b) pairs. Values below each plot represent the spot-to-spot matching ratio.

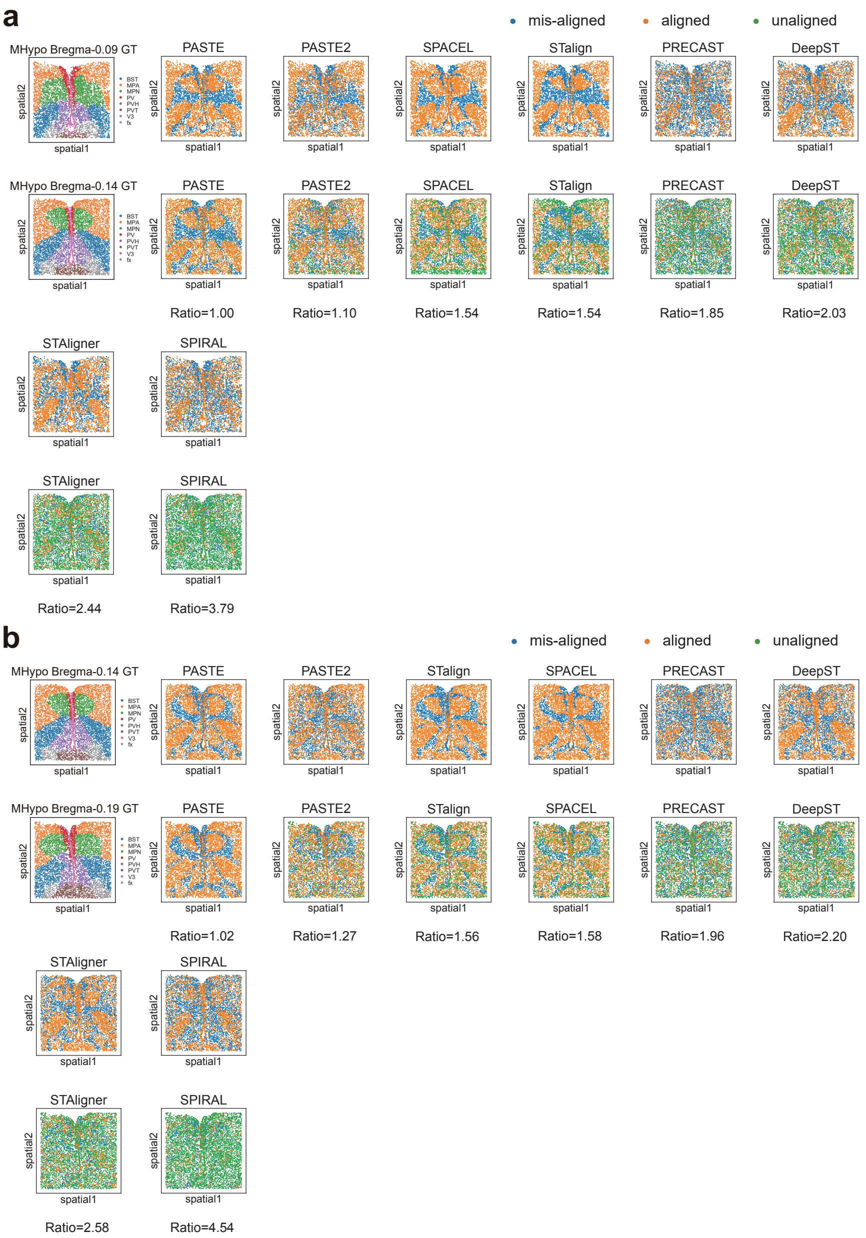

Figure S13: **Visualization plots for alignment-misalignment-unalignment for the MHypo dataset.** **a-b** These visualization plots show aligned spots, misaligned spots, and unaligned spots when aligning the anchor spot from the first (top) slice to the aligned spots on the second (bottom) slice on MHypo Bregma -0.09 - -0.14 (a) and MHypo Bregma -0.14 - -0.19 (b) pairs. Values below each plot represent the spot-to-spot matching ratio.

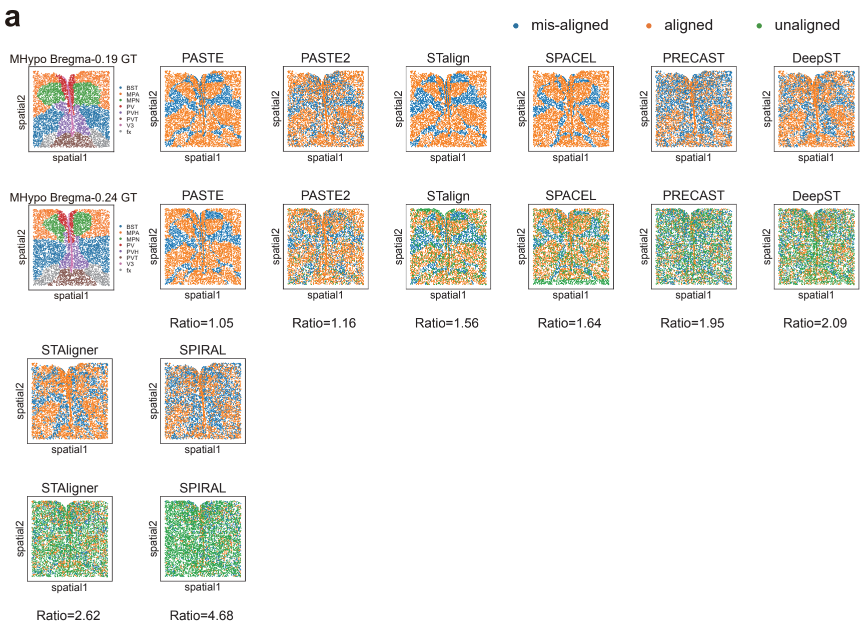

Figure S14: **Visualization plots for alignment-misalignment-unalignment for the MHypo dataset.** **a** These visualization plots show aligned spots, misaligned spots, and unaligned spots when aligning the anchor spot from the first (top) slice to the aligned spots on the second (bottom) slice on MHypo Bregma -0.19 - -0.24 pair. Values below each plot represent the spot-to-spot matching ratio.

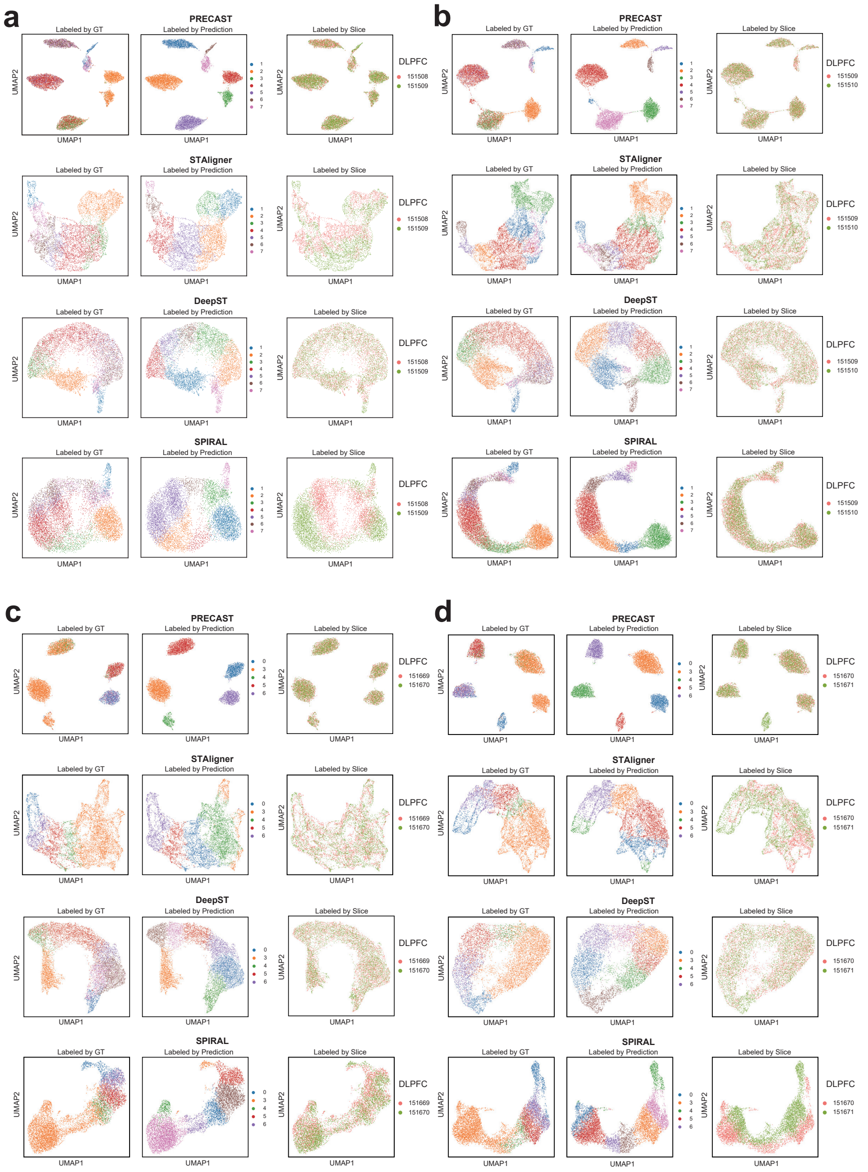

Figure S15: **UMAP plots of low dimensional joint embedding distribution for batch correction for the DLPFC dataset. a-d** These UMAP plots depict the 2D distribution of latent joint embeddings after integration with batch correction by different integration methods on the DLPFC 151508-151509 pair (a), the DLPFC 151509-151510 pair (b), the DLPFC 151669-151670 pair (c), and the DLPFC 151670-151671 pair (d). Each UMAP contains colored spots labeled by three different setups: ground truth (GT), method prediction, and slice index.

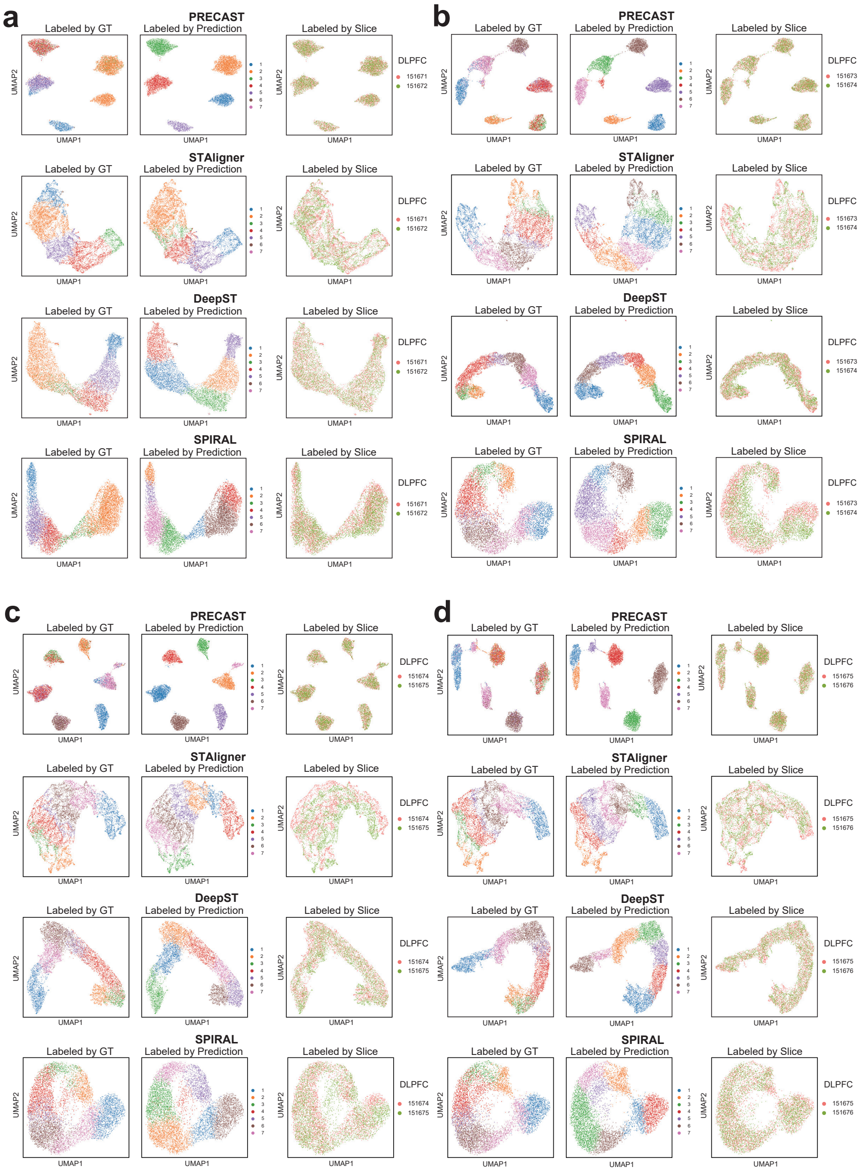

Figure S16: **UMAP plots of low dimensional joint embedding distribution for batch correction for the DLPFC dataset. a-d** These UMAP plots depict the 2D distribution of latent joint embeddings after integration with batch correction by different integration methods on the DLPFC 151671-151672 pair (a), the DLPFC 151673-151674 pair (b), the DLPFC 151674-151675 (c), and the DLPFC 151675-151676 pair (d). Each UMAP contains colored spots labeled by three different setups: ground truth (GT), method prediction, and slice index.

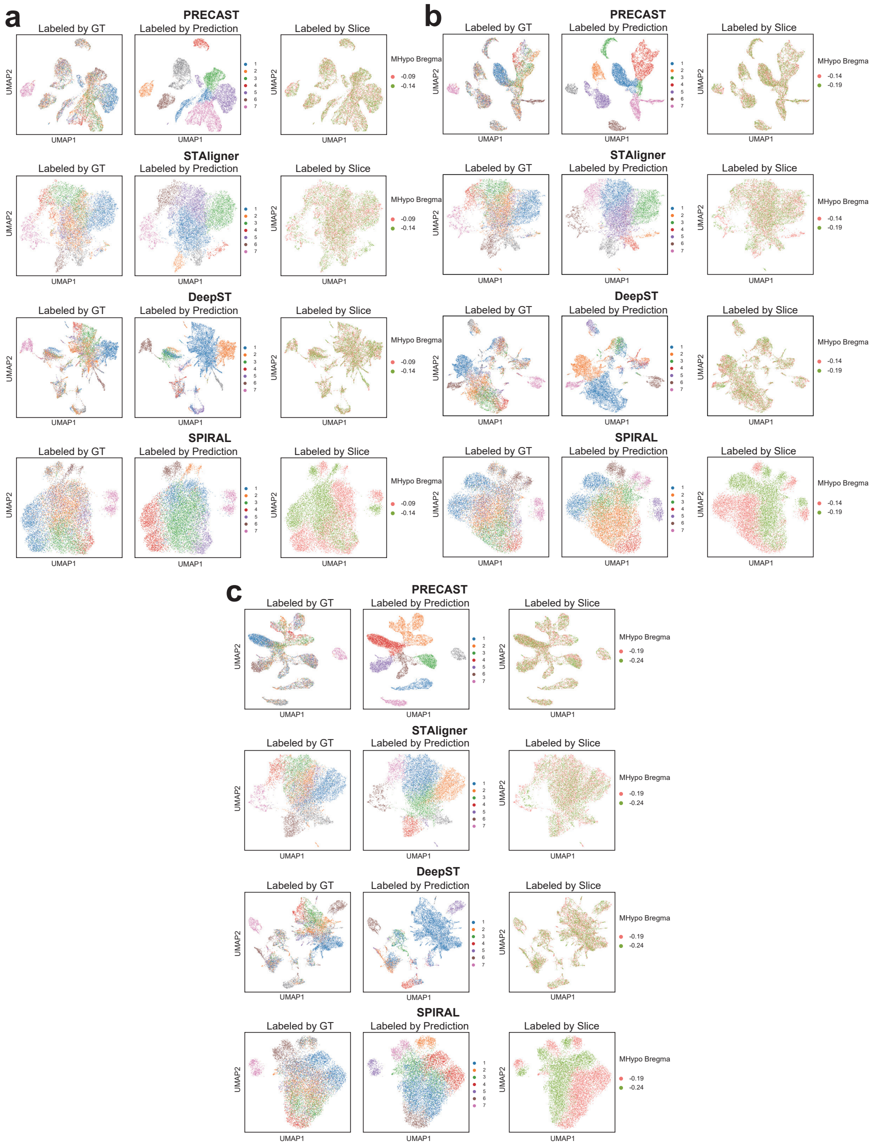

Figure S17: **UMAP plots of low dimensional joint embedding distribution for batch correction for the MHypo dataset. a-c** These UMAP plots depict the 2D distribution of latent joint embeddings after integration with batch correction by different integration methods on the MHypo Bregma -0.09 - -0.14 pair (a), the MHypo Bregma -0.14 - -0.19 pair (b), and the MHypo Bregma -0.19 - -0.24 pair (c). Each UMAP contains colored spots labeled by three different setups: ground truth (GT), method prediction, and slice index.

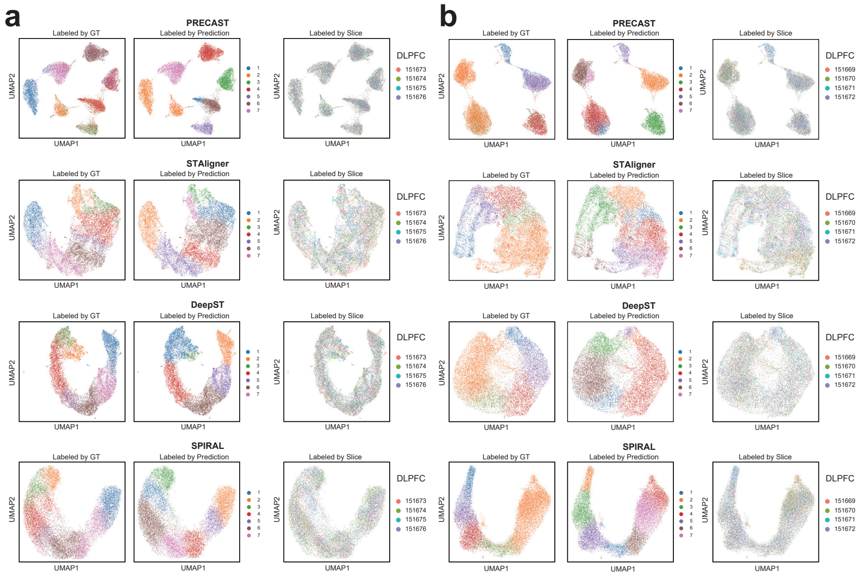

Figure S18: **UMAP plots of low dimensional joint embedding distribution for batch correction. a-b** These UMAP plots depict the 2D distribution of latent joint embeddings after integration with batch correction by different methods on the DLPFC 151673-151676 four slices (a), and the DLPFC 151669-151672 four slices (b). Each UMAP contains colored spots labeled by three different setups: ground truth (GT), method prediction, and slice index.

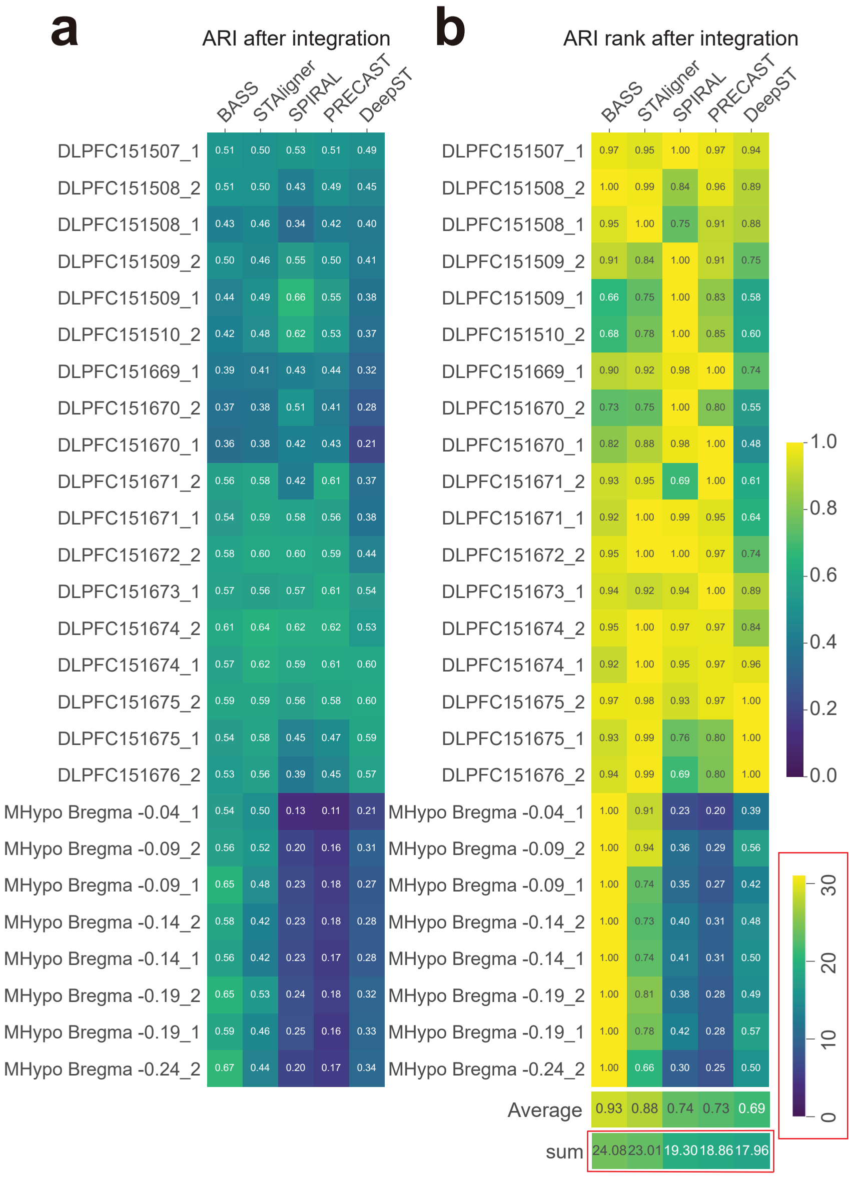

Figure S19: ARI Heatmap after integration. **a** ARI heatmap after integration. Each average ARI value is based on 20 runs. It includes nine DLPFC and four MHypo pairs. **b** Ranking heatmap. This ranking heatmap is created by normalizing all results within the same slice by dividing them by the maximum ARI value (representing the best performance) among all methods, thus standardizing all ARI values to 1. For each method, the best ranking for the sum result is 26, and the best ranking for the average result is 1. The two heatmaps in (a-b) share a color bar ranging from 0 to 1.

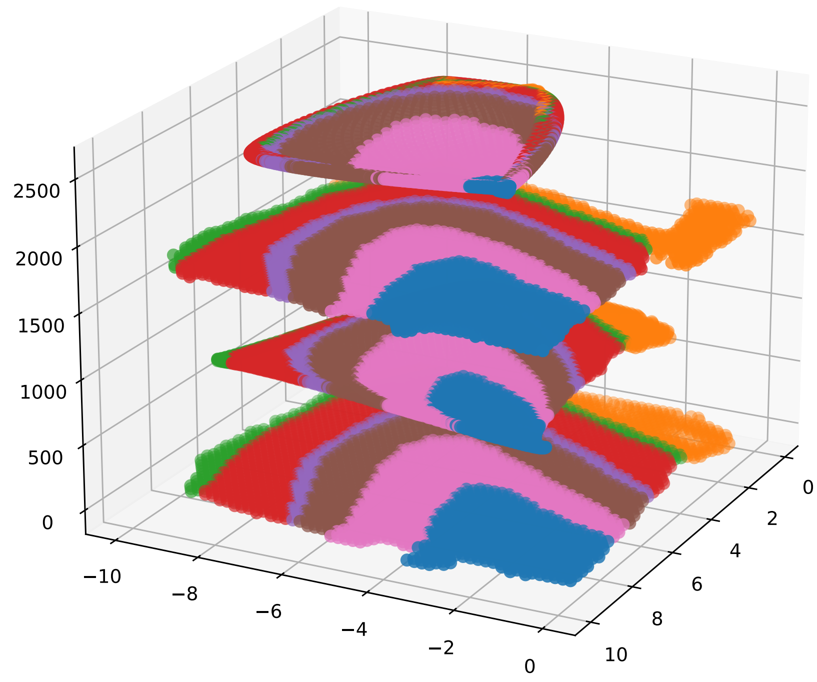

Figure S20: 3D architecture reconstructed from four slices (DLPFC 151673-151676) of DLPFC Sample 3 using GPSA.

References

[1] Townes, F.W., Hicks, S.C., Aryee, M.J., Irizarry, R.A.: Feature selection and dimension reduction for single-cell rna-seq based on a multinomial model. Genome Biology **20**, 1–16 (2019)
