# Supplementary material for: Benchmarking clustering, alignment, and integration methods for spatial transcriptomics
Source: Genome Biol. 2024 Aug 9;25:212. doi: 10.1186/s13059-024-03361-0 (PMC11312151; doi:10.1186/s13059-024-03361-0)
Supplement: Supplementary file 3 — Additional file 3. Review history. [file 13059_2024_3361_MOESM3_ESM.docx]

Review History

**First round of review**

**Reviewer 1**

**Were you able to assess all statistics in the manuscript, including the appropriateness of statistical tests used?**

There are no statistics in the manuscript.

**Comments to author:**

In this manuscript, a benchmark study for spatial clustering, alignment, and integration methods is presented for spatial transcriptomics (ST) data. The study systematically benchmarked 14 state-of-the-art clustering methods, 3 alignment methods, and 5 integration methods on a multitude of simulated and real ST datasets. Metrics such as ARI, layer-wise and spot-to-spot alignment accuracy, and spatial coherence score (SCS) were used for evaluating method performance. The strengths and weakness of each method were analyzed with quantitative and qualitative metrics in details. Following the benchmarking process, the authors provide a comprehensive recommendation for method selection. The codes and tutorials for reproducing the results have been made available on Github.

Overall, this benchmark study is timely and benefit the community by guiding the selection of methods and promoting the development of new methods. While the benchmarking is comprehensive and provides some recommendations, there is still a need for further in-depth analysis and insights into these methods. The main differences between this study and several similar benchmarking studies need to be clarified. There are some major concerns below.

Major comments:

1. While 31 slices of seven datasets are used in the benchmarking of spatial clustering, the dataset size relatively is small as the largest dataset is about 4k spots. Moreover, these data share common characteristics, i.e., the layers in the sample are continuous and have clear boundaries, which may be not challenging enough for these clustering methods. For a more comprehensive benchmarking, larger dataset with more complex tissue structure should be used in the experiment, such as Stereo-seq mouse brain embryo.

2. In the experiment, the authors leverage ARI and spatial coherence score for evaluating the performance of different methods in spatial clustering. However, only using these two metrics might be insufficient to determine whether a method is good or not in certain task. More metrics, such as Normalized Mutual Information (NMI), Adjusted Mutual Information (AMI), homogeneity, etc. can be considered for comprehensive evaluation.

3. In the benchmarking of spatial clustering, datasets from various platforms are used, including 10x Visium, ST, Slide-seq V2. However, in general, applicable data type for each method is specified, and a general method that is suitable for all data types is underexplored. For example, GraphST (Long et al., 2023) is specified for data from platforms 10x Visium, Stereo-seq, Slide-seq. This might be an explanation for vast differences in performance between conST, GraphST, and BASS on some datasets (e.g., Figure 2 c-i).

4. Data preprocessing is a critical step in single-cell or spatial omics analysis. While the authors introduce the common overlapping steps and claimed they provided the specific pipeline of data preprocessing for each method in the GitHub, it is not clear whether different methods share the same data preprocessing. If not, the details of preprocessing for each method can be provided. In addition, it seems that a simple introduction of each method is missing in the manuscript.

5. The authors claim that 'The absence of benchmark studies complicates the section…'. The description may be not accurate as there are at least two similar benchmarking papers. They are "Benchmarking Spatial Clustering Methods with Spatially Resolved Transcriptomics Data (Yuan et al., Nature Methods 2024)" and "Benchmarking cell-type clustering methods for spatially resolved transcriptomics data (Cheng et al., 2023)". The main differences between this benchmarking paper and existing benchmarking papers mentioned above should be discussed in the manuscript.

6. For the methods in the benchmarking, most of them are deep learning models and are sensitive to random seed. Some methods do not fix the seed, such as STAGATE and PRECAST. How do you select their results? In addition, it would be very interesting to explore how these methods are influenced by random seed.

7. It is highly recommended that more further discussions can be conducted on potential directions for the development of analysis methods for spatial omics data, which would be of great benefit the community.

Minor comments:

1. Some methods recently developed for spatial clustering or alignment are missing, such as "Identifying multicellular spatiotemporal organization of cells with SpaceFlow (Ren et al., Nature Communications 2022)", "STalign: Alignment of spatial transcriptomics data using diffeomorphic metric mapping (Clifton et al., Nature Communications 2023)". It would be interesting to discuss them in the paper.

2. Github links for some methods are missing, such as PASTE and BASS.

3. In Figure 2, some fonts are not clear. The color bar for Figure 2 should be added.

4. In Figure 2 c-j, ground truth is for single sample. It is not clear how the boxplot is drawn?

5. In Figure 4 a, the width of the bars in the boxplot is too narrow.

6. In Figure 4 c, it is recommended to remove sample name and retain the method name in the title of spatial clustering for each method.

**Reviewer 2**

**Were you able to assess all statistics in the manuscript, including the appropriateness of statistical tests used?**

Yes, and I have assessed the statistics in my report.

**Comments to author:**

As spatial transcriptomics technologies are increasingly applied to various tissues, and many computational methods have been developed for analyzing spatial transcriptomics data, evaluating the performances of these methods will help biologists to choose the most suitable method to use. This manuscript benchmarked 14 spatial clustering methods and 8 spatial alignment/integration method across ten datasets from both sequencing-based and imaging-based platforms. This benchmark work contains the largest number of computational methods and datasets compared to previous benchmarking work, which I believe provides a more comprehensive insight for choosing computational tools.

However, I have the following concerns.

1. The manuscript lack a description of previous benchmarking work and a comparison whether the findings are consistent with previous conclusions over the shared computational tools. For example: https://doi.org/10.1038/s41592-024-02215-8, https://doi.org/10.1093/bib/bbac475
2. There are additionally two alignment methods that may be included in the benchmarking at least mentioned, GPSA (https://doi.org/10.1038/s41592-023-01972-2) and STalign (https://doi.org/10.1038/s41467-023-43915-7).
3. There are different categories of technologies (e.g. sequencing vs imaging) and different types of tissue (e.g. tumor vs non tumor), which generate data of different properties. It would be of interest to summarize the accuracy according to technologies and tissue types besides the current summary of overall performance.
4. Regarding the stochasticity of the methods.
- Statistical methods (BayesSpace, BASS) were claimed to be deterministic in the manuscript while they are not. They optimize a non-convex function and the solutions highly depend on the random initialization of parameters. Their tutorials (https://www.ezstatconsulting.com/BayesSpace/articles/BayesSpace.html and https://zhengli09.github.io/BASS-Analysis/DLPFC.html) include a step to set a random seed.
- For the stochastic methods, 20 random initializations are used and the average performances are considered in the comparison. I recommend to include the following two summary statistics of the 20 runs: (1) the median, (2) the objective function of each method provides a natural way for model selection, i.e., to select the random seed with the best objective; and thus if the method outputs an objective value, it's worth to compare the random seed associated with the best objective value across methods.
5. It's confusing what preprocessing step is used for each method, whether it is specific to and follow the documentation of each method or only extracting the common steps. This paragraph needs a better description. Additionally, glmPCA is considered to greatly improve the low-dimensional representation than PCA. It worth evaluating whether replacing PCs with glmPCs also lead to a performance change of the clustering/alignment/integration methods.
6. The ground truth associated with each dataset is complicated. It would be clearer if there is a table describing (1) what the ground truth is, (2) what information is used in deriving the ground truth, e.g. marker gene expression or H&E or other expertise annotation, (3) whether the ground truth enables a quantitative comparison or a visual one.
7. The idea of evaluating the spatial clustering accuracy with integrated slices is insightful. However, Fig 7 does not effectively highlight the comparison results.
- Can Fig 2a-b be produced for the accuracy after integration?
- Even though there may not be an overall winner in clustering accuracy, evaluating the degree of performance improvement via integration would still be of interest.
- Currently, the integration only considers closest pairs of slices. What are the z-axis distances between slices? I suggest include the integration between distant slices to provide an expectation of performance for new data with more variable z-axis distances.


Here are my minor comments.
1. The description for distinguishing between alignment methods and integration methods is confusing. What's the criteria for categorizing methods into the two categories?
2. It is hard for the reader to build in their mind a visual comparison in Fig 3a. Is it possible to group cell types such that the grouped ones occupy more contiguous spatial domains and quantitatively compute the ARI?
3. Spatial entropy is used as The Spatial Coherence Score (SCS) to evaluate the coherence in space. In my past experience with it, it may not actually indicate the coherence of clusters: for example, it cannot distinguish between a checkerboard structure of two clusters (not coherent at all) and a contiguous partition in space (very coherent). I don't know of a standard statistics and widely accepted for spatial coherence, but I'm experimenting with the join count statistics (BOUAYAD AGHA Salima and MD Bellefon.Spatial autocorrelation indices, pages 60-62. 2018.). I recommend to explore and include other statistics relevant to spatial coherence.
4. The regression line in Fig 3b is confusing and the linear pattern is not visually apparent. The x-axis of the regression is indicated to be SCS according to Section "Spatial coherence score and runtime analysis for clustering methods", but the x-axis in the plot is equally spaced and seems to be the ranking of SCS among the datasets rather than SCS itself.
5. When evaluating the accuracy of alignment methods, the layer shift evaluation metric makes sense, but Fig 4a is hard to parse. The y-axis label is "layer-wise alignment accuracy", however, it is not the higher the better, but instead the more concentrated leftward the better. Additionally, Fig 5b also has the same y-axis label, but doesn't seem to be the more concentrated leftward the better. Please clarify the y-axis label and consistency between he two plots.

**Authors Response**

**Point-by-point responses to the reviewers’ comments:**

***Reviewer #1:*** *In this manuscript, a benchmark study for spatial clustering, alignment, and integration methods is presented for spatial transcriptomics (ST) data. The study systematically benchmarked 14 state-of-the-art clustering methods, 3 alignment methods, and 5 integration methods on a multitude of simulated and real ST datasets. Metrics such as ARI, layer-wise and spot-to-spot alignment accuracy, and spatial coherence score (SCS) were used for evaluating method performance. The strengths and weakness of each method were analyzed with quantitative and qualitative metrics in details. Following the benchmarking process, the authors provide a comprehensive recommendation for method selection. The codes and tutorials for reproducing the results have been made available on Github.*

*Overall, this benchmark study is timely and benefit the community by guiding the selection of methods and promoting the development of new methods. While the benchmarking is comprehensive and provides some recommendations, there is still a need for further in-depth analysis and insights into these methods. The main differences between this study and several similar benchmarking studies need to be clarified. There are some major concerns below.*

We thank Reviewer 1 for the positive evaluation towards the potential of this benchmarking work and for the insightful comments on this study. We have performed additional analyses to further improve the rigor of this paper. All the changes are highlighted in red in the main manuscript and supplementary information.

*Major comments:*

1. *While 31 slices of seven datasets are used in the benchmarking of spatial clustering, the dataset size relatively is small as the largest dataset is about 4k spots. Moreover, these data share common characteristics, i.e., the layers in the sample are continuous and have clear boundaries, which may be not challenging enough for these clustering methods. For a more comprehensive benchmarking, larger dataset with more complex tissue structure should be used in the experiment, such as Stereo-seq mouse brain embryo.*

Response: The reviewer’s point is well taken. We have added a benchmark analysis on the Stereo-seq mouse embryo for all clustering tools. Figure 2, 3, 5, 6 and Figure S3 have been updated to reflect the clustering performance analysis, and the corresponding text in the results sections has been revised accordingly.

2. *In the experiment, the authors leverage ARI and spatial coherence score for evaluating the performance of different methods in spatial clustering. However, only using these two metrics might be insufficient to determine whether a method is good or not in certain task. More metrics, such as Normalized Mutual Information (NMI), Adjusted Mutual Information (AMI), homogeneity, etc. can be considered for comprehensive evaluation.*

Response: The reviewer’s point is well taken. We have conducted a benchmark analysis in terms of Normalized Mutual Information (NMI), Adjusted Mutual Information (AMI), and homogeneity (HOM) for all clustering tools. Figure 3 and a corresponding Results section titled “Clustering Performance Comparison using NMI, AMI, and HOM” has been added accordingly.

3. *In the benchmarking of spatial clustering, datasets from various platforms are used, including 10x Visium, ST, Slide-seq V2. However, in general, applicable data type for each method is specified, and a general method that is suitable for all data types is underexplored. For example, GraphST (Long et al., 2023) is specified for data from platforms 10x Visium, Stereo-seq, Slide-seq. This might be an explanation for vast differences in performance between conST, GraphST, and BASS on some datasets (e.g., Figure 2 c-i).*

Response: The reviewer raises a valid point. We have added a discussion at the end of the corresponding Results section titled “Performance Comparison of 16 Clustering

Methods” to address the differences in tool performance concerning different ST protocols or tissue types. An additional file (Additional File 1: Table S2) has been included to summarize the parameter settings and descriptions used in the preprocessing steps when benchmarking each method and clarify the applicable ST protocols from each paper for each method.

Since this point also aligns with major point 3 raised by Reviewer 2, we have extended our recommendation table (Table 2) to summarize the accuracy according to ST technologies and tissue types, in addition to the current summary of overall performance. For better readability, we have updated all heatmaps and line plots in Figure 2-3 and 5 by ordering the ST slices or datasets according to various ST protocols.

4. *Data preprocessing is a critical step in single-cell or spatial omics analysis. While the authors introduce the common overlapping steps and claimed they provided the specific pipeline of data preprocessing for each method in the GitHub, it is not clear whether different methods share the same data preprocessing. If not, the details of preprocessing for each method can be provided. In addition, it seems that a simple introduction of each method is missing in the manuscript.*

Response: The reviewer’s point is well taken. We have added an additional file (Additional File 1: Table S2) to summarize the parameter settings and descriptions used in the preprocessing steps when benchmarking each method and to clarify the applicable ST protocols from each paper for each method. In the first Results section titled “ST Datasets Examined and Data Preprocessing,” we have revised the corresponding text for data processing.

We have also added two Methods sections titled “Clustering Methods Overview” and “Alignment and Integration Methods Overview” to provide a brief introduction for each method.

5. *The authors claim that 'The absence of benchmark studies complicates the section...'. The description may be not accurate as there are at least two similar benchmarking papers. They are "Benchmarking Spatial Clustering Methods with Spatially Resolved Transcriptomics Data (Yuan et al., Nature Methods 2024)" and "Benchmarking cell-type clustering methods for spatially resolved transcriptomics data (Cheng et al., 2023)". The main differences between this benchmarking paper and existing benchmarking papers mentioned above should be discussed in the manuscript.*

Response: The reviewer’s point is well taken. This point also aligns with major point 1 raised by Reviewer 2. We now cite these two papers and expanded the Discussion section to address the difference between ours and these benchmark work, while also examining the findings over shared tools.

6. *For the methods in the benchmarking, most of them are deep learning models and are sensitive to random seed. Some methods do not fix the seed, such as STAGATE and PRECAST. How do you select their results? In addition, it would be very interesting to explore how these methods are influenced by random seed.*

Response: The reviewer raises a valid point. Since deep learning-based methods such as STAGATE, ADEPT, and CCST do not fix the randomization seed, we ran 20 iterations and took the average of each metric for comparison instead of selecting a specific result. We performed an analysis to investigate the relationship between loss value, random seed, and performance (in terms of ARI) on STAGATE, ADEPT, and CCST. Our results indicated that clustering performance, measured by ARI, was randomly associated with both the loss value and the selected seed for each deep learning method, making it challenging to select a particular result. We have added Figure S4-6 and a corresponding Results section titled “Random Seed Analysis” for this analysis.

7. *It is highly recommended that more further discussions can be conducted on potential directions for the development of analysis methods for spatial omics data, which would be of great benefit the community.*

Response: The reviewer’s point is well taken. We have added further discussion on potential directions for the development of analysis methods for spatial omics data at the end of the Discussion section.

*Minor comments:*

*1.	Some methods recently developed for spatial clustering or alignment are missing, such as "Identifying multicellular spatiotemporal organization of cells with SpaceFlow (Ren et al., Nature Communications 2022)", "STalign: Alignment of spatial transcriptomics data using diffeomorphic metric mapping (Clifton et al., Nature Communications 2023)". It would be interesting to discuss them in the paper.*

Response: The reviewer’s point is well taken. Since this point also aligns with major point 2 raised by Reviewer 2, we have added two more clustering tools, BANKSY and SpaceFlow, and two more alignment tools, STalign and GPSA, into our analyses. We have updated the corresponding Figures 1-9, 14, Table 1-2, Figures S2-3, S9-14, and revised the corresponding main text.

2. *Github links for some methods are missing, such as PASTE and BASS.* Response: Thanks for pointing out this. We fixed this issue.

*3.	In Figure 2, some fonts are not clear. The color bar for Figure 2 should be added.* Response: We have updated Figure 2 and used the Arial font for it and other figures. To save space, The two heatmaps in (a-b) share a color bar ranging from 0 to 1. This has

been clarified in the figure legend.

4. *In Figure 2 c-j, ground truth is for single sample. It is not clear how the boxplot is drawn?*

Response: Thank you for pointing this out. Since some methods do not set a fixed seed, they can produce varying clustering and evaluation results. To address this, we conducted 20 iterations for each sample to generate the boxplot. This has been clarified in the Figure 2 legend: The box plots illustrate the variability in the ARI on individual slices for certain tools since they do not use a fixed seed.

5. *In Figure 4 a, the width of the bars in the boxplot is too narrow.*

Response: The reviewer’s point is well taken. Figure 4a has been updated to Figure 7 in the revised version. We have adjusted the bar width for better visualization.

6. *In Figure 4 c, it is recommended to remove sample name and retain the method name in the title of spatial clustering for each method.*

Response: The reviewer’s point is well taken. Figure 4c-d has been updated to Figure 8a-b in the revised version. We revised all figure titles in Figure 8a-b and the corresponding Figure S9-14.

***Reviewer #2:*** *As spatial transcriptomics technologies are increasingly applied to various tissues, and many computational methods have been developed for analyzing spatial transcriptomics data, evaluating the performances of these methods will help biologists to choose the most suitable method to use. This manuscript benchmarked 14 spatial clustering methods and 8 spatial alignment/integration method across ten datasets from both sequencing-based and imaging-based platforms. This benchmark work contains the largest number of computational methods and datasets compared to previous benchmarking work, which I believe provides a more comprehensive insight for choosing computational tools.*

We thank Reviewer 2 for the positive evaluation towards the potential of this benchmarking work and for the insightful comments on this study. We have performed additional analyses to further improve the rigor of this paper. All the changes are highlighted in red in the main manuscript and supplementary information.

*However, I have the following concerns.*

1. *The manuscript lack a description of previous benchmarking work and a comparison whether the findings are consistent with previous conclusions over the shared computational tools. For example:*

[*https://www.nature.com/articles/s41592-024-02215-8*](https://www.nature.com/articles/s41592-024-02215-8)

Response: The reviewer’s point is well taken. This point also aligns with major point 5 raised by Reviewer 1. we cited these papers and added discussion at the Discussion section to address the difference between ours and these benchmark papers, while also examining the findings over shared tools.

2. *There are additionally two alignment methods that may be included in the benchmarking at least mentioned, GPSA* [*(*https://www.nature.com/articles/s41592-023-01972-2](https://www.nature.com/articles/s41592-023-01972-2) *)and STalign* [*(*https://www.nature.com/articles/s41467-023-43915-7](https://www.nature.com/articles/s41467-023-43915-7) *).*

Response: The reviewer’s point is well taken. Since this point also aligns with minor point 1 raised by Reviewer 1, we have added two more clustering tools, BANKSY and SpaceFlow, and two more alignment tools, STalign and GPSA, into our analyses. We have updated the corresponding Figures 1-9, 14, Tables 1-2, Figures S2-3, S9-14, and revised the corresponding main text.

3. *There are different categories of technologies (e.g. sequencing vs imaging) and different types of tissue (e.g. tumor vs non tumor), which generate data of different properties. It would be of interest to summarize the accuracy according to technologies and tissue types besides the current summary of overall performance.*

Response: The reviewer raises a valid point. We have extended our recommendation table (Table 2) to summarize the accuracy according to ST technologies and tissue types, in addition to the current summary of overall performance. For better readability, we have updated all heatmaps and line plots in Figure 2-3 and 5 by ordering the ST slices or datasets according to various ST protocols.

Since this point also aligns with major point 3 raised by Reviewer 1, we have also expanded the text at the end of the corresponding Results section titled “Performance Comparison of 16 Clustering Methods” to address the differences in tool performance concerning different ST protocols or tissue types. An additional file (Additional File 1: Table S2) has been included to summarize the parameter settings and descriptions used in the preprocessing steps when benchmarking each method and clarify the applicable ST protocols from each paper for each method.

4. *Regarding the stochasticity of the methods.*

*- Statistical methods (BayesSpace, BASS) were claimed to be deterministic in the manuscript while they are not. They optimize a non-convex function and the solutions highly depend on the random initialization of parameters. Their tutorials* [*(*](https://www.ezstatconsulting.com/BayesSpace/articles/BayesSpace.html)[https://www.ezstatconsulting.com/BayesSpace/articles/BayesSpace.html](https://and) *and* [https://zhengli09.github.io/BASS-Analysis/DLPFC.htm](https://zhengli09.github.io/BASS-Analysis/DLPFC.html)l)*include a step to set a random seed.*

*- For the stochastic methods, 20 random initializations are used and the average performances are considered in the comparison. I recommend to include the following two summary statistics of the 20 runs: (1) the median, (2) the objective function of each method provides a natural way for model selection, i.e., to select the random seed with the best objective; and thus if the method outputs an objective value, it's worth to compare the random seed associated with the best objective value across methods.*

Response: These points are well taken. We have revised the corresponding text.

We have double-checked the statistical methods and contacted their authors for clarification of the objective function. Most statistical methods do not include an objective function for performance optimization, and all statistical methods fix the seed to allow users to produce deterministic output. For example, BASS has no objective

function, and its value cannot be calculated. BayesSpace has an objective function that we can use, “NumericVector plogLik,” to output its value.

We performed an analysis to investigate the relationship between objective function value, random seed, and performance (in terms of ARI) for BayesSpace and BASS. For BASS, we did not use objective function value, but only the random seed and performance. Our results indicated that clustering performance, measured by ARI, was randomly associated with both the objective function value and the selected seed, making it challenging to select a particular result based on seed or the objective function value. We have added Figure S7-8 and a corresponding Results section titled “Random Seed Analysis” for this analysis.

*5. It's confusing what preprocessing step is used for each method, whether it is specific to and follow the documentation of each method or only extracting the common steps. This paragraph needs a better description. Additionally, glmPCA is considered to greatly improve the low-dimensional representation than PCA. It worth evaluating whether replacing PCs with glmPCs also lead to a performance change of the clustering/alignment/integration methods.*

Response: The reviewer’s points are well taken. This point also aligns with major point 4 raised by Reviewer 1. We followed the documentation of each method for the preprocessing steps and running each tool. We have added an additional file (Additional File 1: Table S2) to summarize the parameter settings and descriptions used in the preprocessing steps when benchmarking each method and to clarify the applicable ST protocols from each paper for each method. In the first Results section titled “ST Datasets Examined and Data Preprocessing,” we have revised the corresponding text for data processing.

Regarding glmPCA, because all alignment and integration tools do not use PCA for dimension reduction. We selected four representative clustering tools - SpaGCN, CCST, BASS, and BayesSpace - that utilize PCA in their preprocessing or postprocessing steps, to analyze whether replacing principal components (PCs) with GLM-PCs enhances performance. In the preprocessing steps for BASS and BayesSpace, we substituted the standard normalization (logarithm of counts per million, spatially/highly variable genes selection, and PCA) with GLM-PCA. In the preprocessing steps for SpaGCN, we replaced the standard normalization (logarithm of counts per million and PCA) with GLM-PCA. For CCST, we substituted principal PCs with GLM-PCs for learned latent embeddings in the postprocessing steps. We then analyzed whether this substitution enhances clustering performance in the DLPFC dataset. Our results, as depicted in Figure S1, indicate that SpaGCN, CCST, and BASS did not exhibit significant performance difference when using GLM-PCA compared to PCA. In

contrast, the performance of BayesSpace improved in 10 out of 12 slices for the DLPFC dataset. While it is challenging to conclusively state that GLM-PCA is superior to PCA in the ST field, it has the potential to enhance the performance of certain tools compared to conventional PCA. More details are outlined in Supplementary Results section titled “The impact of GLM-PCA versus PCA on clustering performance”.

Figure S1. ARI Bar Plots for preprocessing or postprocessing with PCA versus GLM-PCA. **a** ARI bar plots for SpaGCN using PCA versus GLM-PCA during the preprocessing step. **b** ARI bar plots for CCST using PCA versus GLM-PCA during the postprocessing step. **c** ARI bar plots for BASS using PCA versus GLM-PCA during the preprocessing step. **d** ARI bar plots for BayesSpace using PCA versus GLM-PCA during the preprocessing step.

*6. The ground truth associated with each dataset is complicated. It would be clearer if there is a table describing (1) what the ground truth is, (2) what information is used in deriving the ground truth, e.g. marker gene expression or H&E or other expertise annotation, (3) whether the ground truth enables a quantitative comparison or a visual one.*

Response: The reviewer raises a valid point. We have added an additional file (Additional File 1: Table S1) to describe details about the ground truth, the information used to derive the ground truth, and whether the ground truth enables a quantitative comparison or a visual one. This table corresponds to the first result section titled “ST datasets examined and data preprocessing”.

*7. The idea of evaluating the spatial clustering accuracy with integrated slices is insightful. However, Fig 7 does not effectively highlight the comparison results.*

*- Can Fig 2a-b be produced for the accuracy after integration?*

*- Even though there may not be an overall winner in clustering accuracy, evaluating the degree of performance improvement via integration would still be of interest.*

*- Currently, the integration only considers closest pairs of slices. What are the z-axis distances between slices? I suggest include the integration between distant slices to provide an expectation of performance for new data with more variable z-axis distances.*

Response: The reviewer’s points are well taken. Figure 7 has been updated to Figure 11a-b in the revised version.

1) We have included Figure S19 for ARI Heatmap after integration. Our results demonstrated that BASS achieved the best average and sum rank after integration, followed by STAligner and SPIRAL. These three tools are the ones we highlighted in this section for having relatively better performance compared to the rest.
2) We have added Figure 11d and updated Results section titled “Integration methods enhance domain identification through joint embedding” to discuss the degree of performance improvement via integration. The updated main text is provided below:

“*Although no clear overall winner emerged after integration, integration analysis showed some improvement in clustering accuracy compared to single-slice analysis within certain tools. Specifically, both PRECAST and DeepST exhibited enhanced clustering accuracy after integration (Fig. 11d). Across a total of 26 before-and-after pair conditions for two datasets, PRECAST's average ARI increased from 0.363 before integration to 0.411 after, though this change was not statistically significant (p=0.3). In contrast, DeepST exhibited a notable increase in clustering accuracy, with the average ARI improving from 0.285 before integration to 0.395 after, which was statistically significant (p=0.0006). Meanwhile, BASS did not show any significant improvement in clustering accuracy through integration, with its average ARI slightly changing from 0.517 before to 0.532 after integration (p=0.5)*.”

**3)** We have clarified the z-distance in the title of each subplot of Figure 11a-b and the corresponding main text. We have added Figure 11c and updated Results section titled “Integration methods enhance domain identification through joint embedding” to explore how physical distance between slices affects integration. The updated main text is provided below.

*“We investigated every adjacent consecutive slice pair in the above before and after integration analysis. The distant DLPFC slice pairs such as 151670 - 151671 and 151508 - 151509 posed challenges for all methods to improve clustering accuracy after integration. To explore how the physical distance between slices affects integration, we analyzed the ARI of all tools at four distances from the Bregma in the MHypo dataset. Specifically, we examined distances of 0.05mm, 0.1mm, 0.15mm, and 0.2mm, using slices at Bregma -0.04 and -0.24 as fixed anchor points. This analysis included comparisons across seven distinct pairings from Bregma -0.04 to -0.24, helping to discern the impact of slice distance on integration effectiveness. We plotted the ARI of two anchor slices against the increasing distance between slices, observing two different outcomes (Fig. 11c): 1) For BASS and DeepST, integration led to an improvement in the ARI of both anchor slices (surpassing the dashed line that represents the ARI for a single anchor slice before integration) when the distance between the slices was small. However, the ARI of the anchor slices declined as the distance between the slices increased. This indicated that integration could reduce the clustering accuracy of the anchor slice if the slice distance was sufficiently large (dropping below the corresponding dashed line). 2) For PRECAST, STAligner, and SPIRAL, integrating with slices that were either close or distant did not impact the clustering accuracy of the anchor slices. In conclusion, integration can enhance clustering for individual slices, but the effectiveness of this improvement depends on the distance between slices for each specific dataset.”*

*Here are my minor comments.*

*1. The description for distinguishing between alignment methods and integration methods is confusing. What's the criteria for categorizing methods into the two categories?*

Response: The reviewer’s point is well taken. We clarified the statement below in the Introduction section. *“Alignment methods are designed to align or match spots or cells from different ST sections or datasets to a common spatial or anatomical reference. These methods are critical for correcting distortions or differences in tissue sections,*

*ensuring consistency across samples. Integration methods primarily merge data from various sources or conditions to create a comprehensive dataset, enhancing data robustness and revealing broader patterns not apparent in individual datasets. These techniques excel at adjusting for batch effects and normalizing data. Some tools can perform both alignment and integration tasks.”*

2. *It is hard for the reader to build in their mind a visual comparison in Fig 3a. Is it possible to group cell types such that the grouped ones occupy more contiguous spatial domains and quantitatively compute the ARI?*

Response: The reviewer’s points are well taken. Figure 3a has been updated to Figure 4c in the revised version. For better visualization, we have extracted clusters from each method to match these seven key distinguished anatomical regions, CA1, CA2, CA3, dentate gyrus (DG), third ventricle (V3), medial habenula (MH), and lateral habenula (LH).

To quantitatively evaluate all methods for these regions, we conducted a manual region-based annotation of “CA1_CA2_CA3” and DG regions based on existing cell type annotations. This manual annotation (shown in Fig. 4d) served as the ground truth for calculating clustering performance, measured by ARI, NMI, AMI, and HOM. Our results indicated that PRECAST exhibited the highest overall performance across all four metrics, followed sequentially by GraphST, SpaceFlow, ADEPT, BANKY, and STAGATE (Fig. 4e). We have updated this analysis in the Results section titled “Qualitative and quantitative benchmarking of a Slide-seq v2 dataset”.

Unfortunately, it was not possible to perform manual region-based annotation for V3, MH, and LH regions based on the cell type annotations.

3. *Spatial entropy is used as The Spatial Coherence Score (SCS) to evaluate the coherence in space. In my past experience with it, it may not actually indicate the coherence of clusters: for example, it cannot distinguish between a checkerboard structure of two clusters (not coherent at all) and a contiguous partition in space (very coherent). I don't know of a standard statistics and widely accepted for spatial coherence, but I'm experimenting with the join count statistics (BOUAYAD AGHA Salima and MD Bellefon.Spatial autocorrelation indices, pages 60-62. 2018.). I recommend to explore and include other statistics relevant to spatial coherence.*

Response: The reviewer’s points are well taken. We have added three additional widely recognized metrics - average silhouette width (ASW), CHAOS, and percentage of abnormal spots (PAS) – to quantify data complexity in terms of spatial continuity using

ground truth labels for each dataset. We have updated Figure 5g-j and revised main text in the Result section titled “Spatial continuity analysis for clustering methods” for this analysis.

We have also used these metrics to assess the spatial continuity of identified domain by different methods. We have updated Figure 5a-f and main text in the Result section titled “Spatial continuity analysis for clustering methods” for this analysis.

4. *The regression line in Fig 3b is confusing and the linear pattern is not visually apparent. The x-axis of the regression is indicated to be SCS according to Section "Spatial coherence score and runtime analysis for clustering methods", but the x-axis in the plot is equally spaced and seems to be the ranking of SCS among the datasets rather than SCS itself.*

Response: The reviewer raises a valid point. Figure 3b has been updated to Figure 5j in the revised version. We have used SCS value as the x-axis label and replotted this figure with a regression line. We have also reported the Pearson correlation coefficient and p value, and revised the main text in the corresponding Result section titled “Spatial continuity analysis for clustering methods” for this analysis.

5. *When evaluating the accuracy of alignment methods, the layer shift evaluation metric makes sense, but Fig 4a is hard to parse. The y-axis label is "layer-wise alignment accuracy", however, it is not the higher the better, but instead the more concentrated leftward the better. Additionally, Fig 5b also has the same y-axis label, but doesn't seem to be the more concentrated leftward the better. Please clarify the y-axis label and consistency between the two plots.*

Response: Thanks for pointing out this. Figure 4a has been updated to Figure 7a in the revised version. Yes, the more concentrated leftward, the better. For better visualization in Figure 7a-b, we have plotted the layer-wise alignment accuracy and sorted the tools in descending order based on the accuracy for layer shift of 0.

Figure 5b has been updated to Figure 9b-e in the revised version. We have revised the y axis label to “layer-wise alignment accuracy (layer shift = 0)”. For this plot, we have only included the layer-wise alignment accuracy for layer shift of 0 across different scenarios (different overlapping percentage or pseudocount perturbation). We have clarified the corresponding main text.

**Second round of review**

**Reviewer 2**

The authors have satisfactorily addressed my previous comments.
